# *Burkholderia cenocepacia* transcriptome during the early contacts with giant plasma membrane vesicles derived from live bronchial epithelial cells

**DOI:** 10.1038/s41598-021-85222-5

**Published:** 2021-03-11

**Authors:** Andreia I. Pimenta, Nuno Bernardes, Marta M. Alves, Dalila Mil-Homens, Arsenio M. Fialho

**Affiliations:** 1 iBB-Institute for Bioengineering and Biosciences, Biological Sciences Research Group, Av. Rovisco Pais 1, 1049-001 Lisbon, Portugal; 2 CQE Instituto Superior Técnico, Departamento de Engenharia Química, Universidade de Lisboa, Av. Rovisco Pais, 1049-001 Lisbon, Portugal; 3 Department of Bioengineering, Instituto Superior Técnico, University of Lisbon, Lisbon, Portugal

**Keywords:** Cell biology, Microbiology, Molecular biology

## Abstract

*Burkholderia cenocepacia* is known for its capacity of adherence and interaction with the host, causing severe opportunistic lung infections in cystic fibrosis patients. In this work we produced Giant Plasma Membrane Vesicles (GPMVs) from a bronchial epithelial cell line and validated their use as a cell-like alternative to investigate the steps involved in the adhesion process of *B. cenocepacia*. RNA-sequencing was performed and the analysis of the *B. cenocepacia* K56-2 transcriptome after the first contacts with the surface of host cells allowed the recognition of genes implicated in bacterial adaptation and virulence-associated functions. The sensing of host membranes led to a transcriptional shift that caused a cascade of metabolic and physiological adaptations to the host specific environment. Many of the differentially expressed genes encode proteins related with central metabolic pathways, transport systems, cellular processes, and virulence traits. The understanding of the changes in gene expression that occur in the early steps of infection can uncover new proteins implicated in *B. cenocepacia-*host cell adhesion, against which new blocking agents could be designed to control the progression of the infectious process.

## Introduction

*Burkholderia cenocepacia* is an opportunistic pathogen known for its capacity of adherence and ability to establish infection and cause disease in cystic fibrosis (CF) patients and other immunocompromised individuals^1,2^. *B. cenocepacia* has many virulence factors that include a broad variety of adhesins, invasins, secretion systems, extracellular enzymes and toxins, and quorum sensing systems^3,4^. Regarding bacterial adhesion, the machinery used is wide and largely complex and several surface molecules like adhesins, flagella, outer membrane proteins (Omp) or lipoproteins have been reported to mediate *B. cenocepacia* attachment towards the host cell^5–9^. To date, the cable pili (Cbl), their associated adhesin (BapA) and trimeric autotransporter adhesins are the only well-documented adhesins in Bcc species^9–13^.

Bacterial contact to host cells has been known as a pivotal step in the host–pathogen interaction^14,15^. The capacity to sense environmental changes and physical barriers of the host makes the pathogen able to alter and adapt its metabolism, regulation, and virulence^16–21^. *Haemophilus influenzae* was reported to modulate the transcription of virulence-associated genes, like adhesins, and genes involved in central metabolism and stress-induced defense mechanisms during adherence to epithelial cells^21^. The study of the complete bacterial transcriptome upon adhesion to host cells could provide significant information to study pathogens’ behavior. In past years, several transcriptional profiling studies have been performed during bacterial infection^20–22^. Simultaneous transcriptomic analysis of bacterial and their host cells are currently conducted to understand the overall alterations that occur in the context of host–pathogen interactions^21^. Nonetheless, the isolation and further analysis of RNA from adherent bacteria could be demanding and hard to achieve concerning the limiting number of available organisms per cell. For that reason, the adhesion to an in vitro membrane system free from contaminant host genetic material could be an easy alternative to extract bacterial RNA with appropriate quantity and quality and to perform further transcriptomic studies.

Giant plasma membrane vesicles (GPMVs) are a model to study the cellular membrane structure and functional interactions. Contrary to other systems in use, like GUVs (giant unilamellar vesicles), GPMVs are more stable, and mimic the constitution of the plasma membrane as they are formed by a chemically induced plasma membrane vesiculation^23^. On contrast, GUVs are self-assembled structures that can be formed from defined lipid mixtures whose composition simulate the natural cell membranes and could contain cholesterol or selected proteins depending on the pretended application or study^24^. GPMV research has advanced through the years and lead to break-through studies of the biophysical properties of the cellular membranes. The similarities to the host cell membranes make giant plasma vesicles a new potential model to study host–pathogen interactions and fulfil a gap of knowledge.

With this work we aim to develop a new technique that allows an easy and efficient recovery of *B. cenocepacia* RNA after contact with host cell membranes. For that, GPMVs were produced from a bronchial epithelial cell line and adhesion assays with *B. cenocepacia* K56-2 were performed. The optimizations applied for both the GPMVs production and bacterial adhesion procedures lead to a final recovery of *B. cenocepacia* RNA samples with high levels of quality and purity to perform RNA-sequencing analysis. The analysis of the *B. cenocepacia* transcriptome after the first contact with the surface of host cells opened new insights regarding the bacterial adaptation and virulence-associated alterations that take place in the early stages of infection. Furthermore, to the best of our knowledge this is the first methodological approach using GPMVs as a tool to study the mechanisms of pathogens interaction with host cells.

## Results

### Production and characterization of GPMVs derived from bronchial epithelial cells

**Figure 1:**
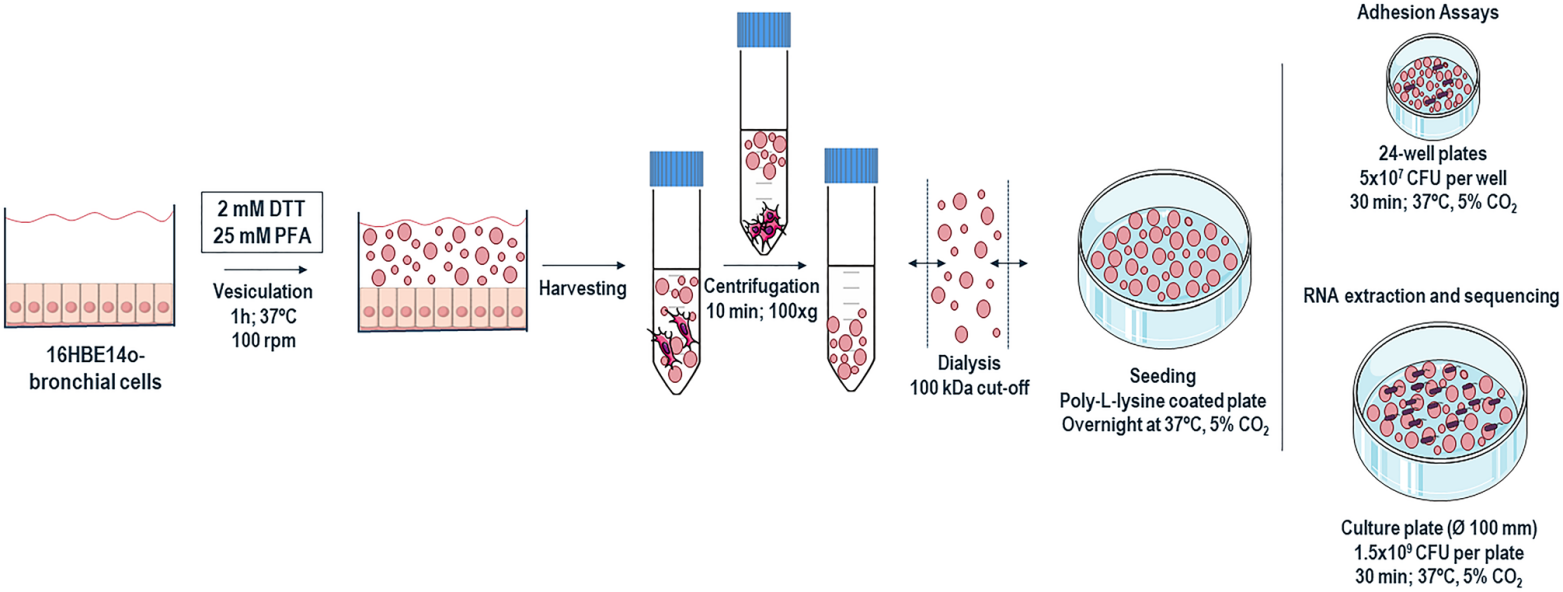
Schematic representation of Giant Plasma Membrane Vesicles production and purification. Briefly, the cells were treated with GPMV-reagent containing PFA and DTT during 1 h to allow vesiculation. The starting cell culture should be prepared in a culture area 8 × higher than the surface where the purified GPMVs will be applied, in order to achieve a confluent layer of vesicles. After incubation, GPMV-enriched supernatant was collected and centrifuged to sediment cellular debris. The clear GPMVs suspension was dialyzed to remove the PFA and DTT. The GPMV suspension was also concentrated and seeded in a poly-L-lysine coated plate, overnight at 37 °C, 5% CO_2_ for further assays. For adhesion assays, 24-well plates were used, and adhesion was performed with 5 × 10^7^ *B. cenocepacia* K56-2 CFU per well. For total RNA extraction and further RNA sequencing analysis, adhesion was performed in a culture plate of Ø 100 mm using 1.5 × 10^9^ *B. cenocepacia* K56-2 CFU per plate. In both cases, bacteria were allowed to adhere for 30 min, at 37 °C, 5% CO_2_. The figure was created using materials from SMART- Servier medical art (https://smart.servier.com/).

**Figure 2:**
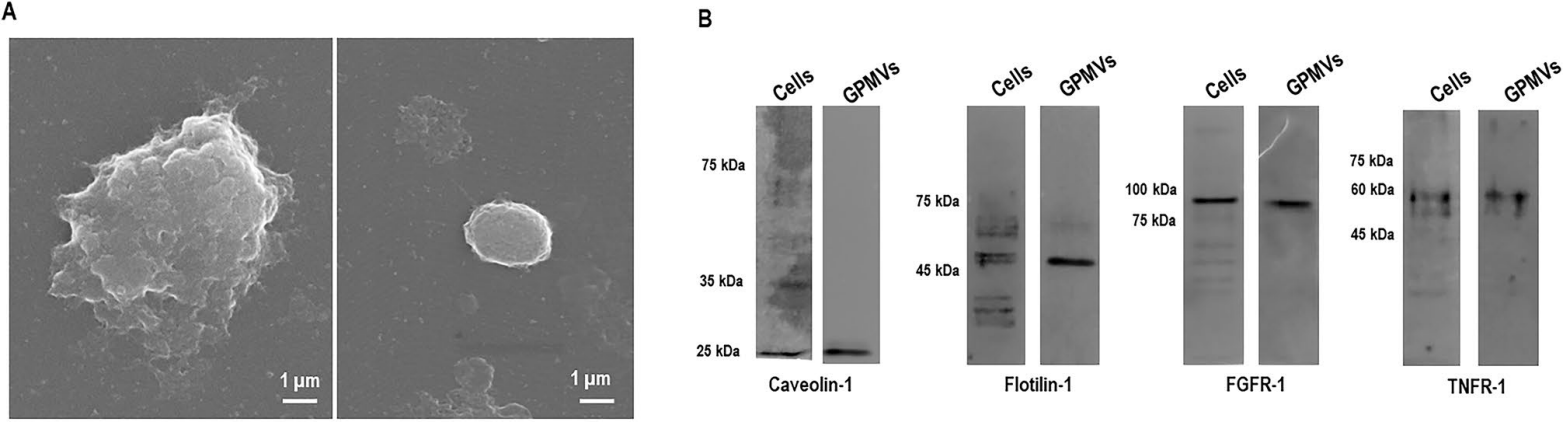
Scanning electron microscopy (SEM) images of 16HBE14o- produced GPMVs (**A**). A scale bar is presented in every SEM image. Western Blot analysis of 16HBE14o- cellular and vesicular protein extracts. The presence of Caveolin-1, Flotillin-1, FGFR-1 and TNFR-1 was analyzed using specific antibodies. The exposure time was optimized for each target, ranging from 1 to 10 min. NZYColour protein marker II was used. The presented WB images were obtained by merging the capture of the protein marker with the chemiluminescence acquisition of the target proteins using the merge tool of Fusion Solo (Viber Lourmat) equipment. Original data are presented as Supplementary Material (**B**).

The first step of this work involved the production and characterization of GPMVs from a bronchial epithelial cell line (16HBE14o-). The optimized protocol is represented in Fig. 1. The morphology of the produced GPMVs was assessed by SEM (Scanning Electron Microscopy) and the images are presented in Fig. 2A. The visualized GPMVs have a spherical-like shape with a size range of 3–5 μm in diameter; they present a rough surface with several structures that seem to stretch over their exterior. (Fig. 2A). Western blot analysis was also performed to compare the protein compositions of GPMVs and cell membranes. TNFR-1 (tumor necrosis factor receptor-1), flotillin-1, caveolin-1 and FGFR-1 (fibroblast growth factor receptor-1) were selected as target proteins. The obtained results show the presence of all tested proteins in both samples (plasma vesicles and cells) (Fig. 2B).

### *B. cenocepacia* K56-2 efficiently adheres to 16HBE14o- derived GPMVs

**Figure 3:**
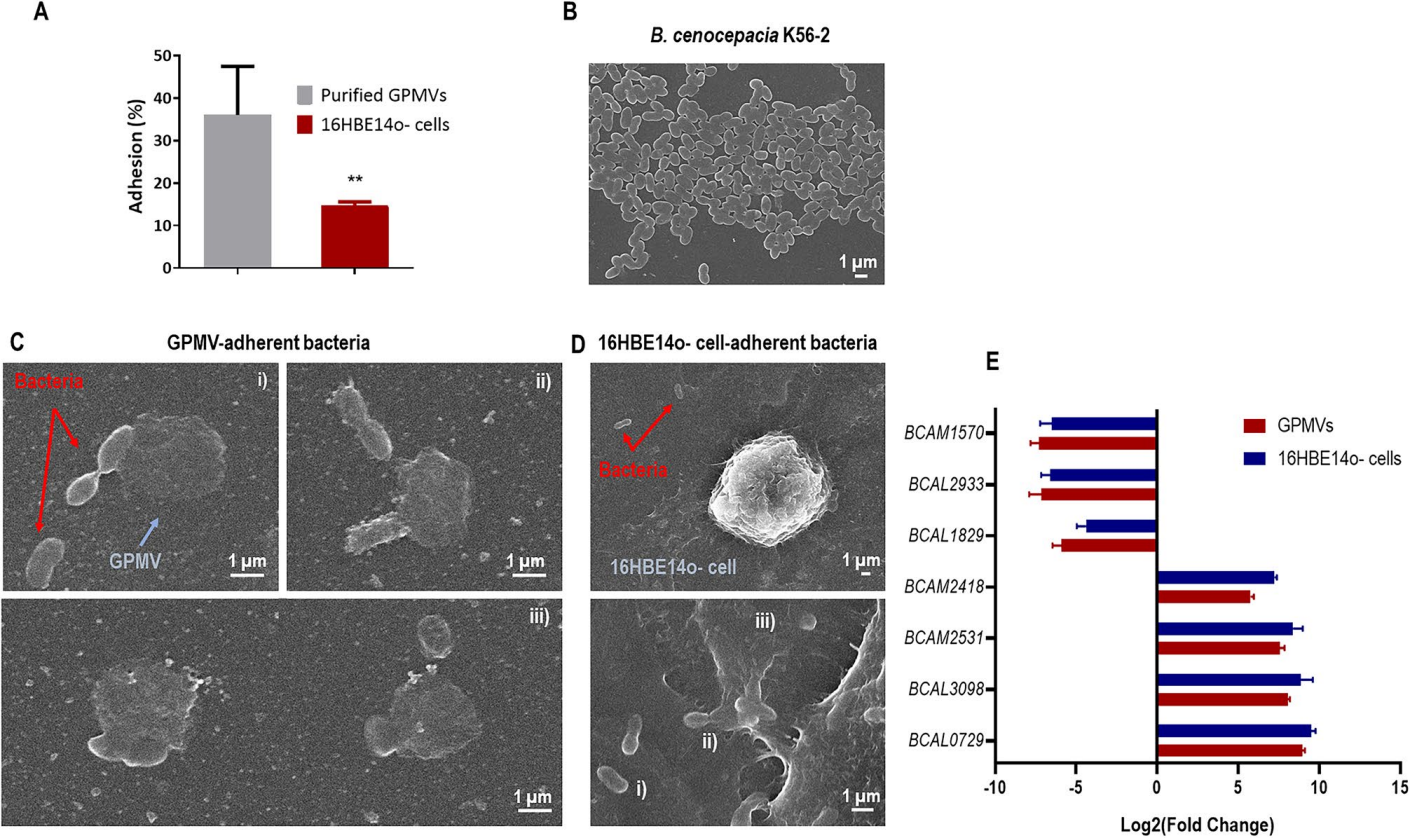
*B. cenocepacia* K56-2 interaction with 16HBE14o- derived GPMVs. *B. cenocepacia* K56-2 adhesion to 16HBE14o- cells and GPMVs (**A**). Adhesion assays were performed for 30 min against 16HBE14o- GPMVs and against cells as a comparison method. *B. cenocepacia* adhere more efficiently to purified GPMVs than to cells. Also, adhesion percentage to GPMVs is higher than the adhesion to the original cells. (****P < 0.0001; **P < 0.01). Error bars indicate the standard deviation. Scanning electron microscopy (SEM) images of *B. cenocepacia* K56-2 cells (**B**), bacteria-GPMV adhesion (**C**), and bacteria-16HBE14o—cell adhesion (**D**). Bacteria are highlighted by red arrows and GPMVs by blue ones. A scale bar is presented in every SEM image. Expression of *B. cenocepacia* K56-2 genes (**E**). Transcription levels of *B. cenocepacia* K56-2 *BCAM02418*, *BCAM0729*, *BCAL1829*, *BCAL293*, *BCAL3098*, *BCAM1570* and *BCAM2531* were obtained by qRT-PCR from bacteria adherent to 16HBE14o- cells and GPMVs (30 min of contact). Results were normalized to expression of the housekeeping *sigA* gene. Expression levels are represented as relative values in comparison to the expression levels in standard LB growth. All the results are from three independent experiments. Differences between both groups of genes were found to be non-significant (*P* > 0.05).

We next optimize a *B. cenocepacia* adhesion assay using a monolayer of 16HBE14o- derived GPMVs. As a comparative assay, adhesion to 16HBE14o- cells was also performed with the same bacterial inoculum. The obtained results are represented in Fig. 3A. When compared to the adhesion values obtained after contact with 16HBE14o- cells, the percentage of *B. cenocepacia* adhesion to GPMVs was considerable superior. This increase could be related to biophysical alterations on the plasma membrane during the vesiculation, which could make GPMVs more prone to interact with pathogenic bacteria. The results suggest that GPMVs, as cell derived-membrane systems, mimic cell membranes of 16HBE14o- cells and thereby may serve as an alternative to living systems.

To confirm *B. cenocepacia* K56-2 interaction with 16HBE14o- GPMVs, SEM images were obtained. The results are presented in Fig. 3 and show images of *B. cenocepacia* K56-2 (B), and the bacterial adherence to vesicles (C) and cells (D). Concerning the interaction between GPMVs and *B. cenocepacia* cells (Fig. 3C), bacterial cells with rod-like shape are clearly in contact with the GPMVs. This contact can be observed to occur in different stages. In an initial stage, bacteria are in contact with GPMV surface (1), in a following step, bacteria look to be embedded inside GPMV (2) and later a fusion-like process seems to occur (3). When comparing with Fig. 3D, similar events can be seen between the bacteria and the cell, where an initial contact phase can be observed (1), followed by an imbibition of the bacterial cells into the cell (2) and fusion (3).

To verify if the adhesion to GPMVs conferred the same *stimuli* to *B. cenocepacia* as the adhesion to host cells, qRT-PCR was performed using both samples—RNA extracted from bacteria adherent to GPMVs and epithelial cells (Fig. 3E). The results in Fig. 3E indicate that after adhesion to GPMVs, *B. cenocepacia* gene transcription seems to be altered in the same pattern that after adhesion to epithelial cells. In both cases, *BCAM2418*, *BCAM2531*, *BCAL3098* and *BCAL0729* genes are induced while *BCAM1570*, *BCAL2933* and *BCAL1829* genes were found to be repressed. These results support that GPMVs may be an alternative model to study the early stages of host-bacteria interactions.

### Adherence to 16HBE14o-GPMVs alter the transcriptomic profile of *B. cenocepacia* K56-2

**Figure 4:**
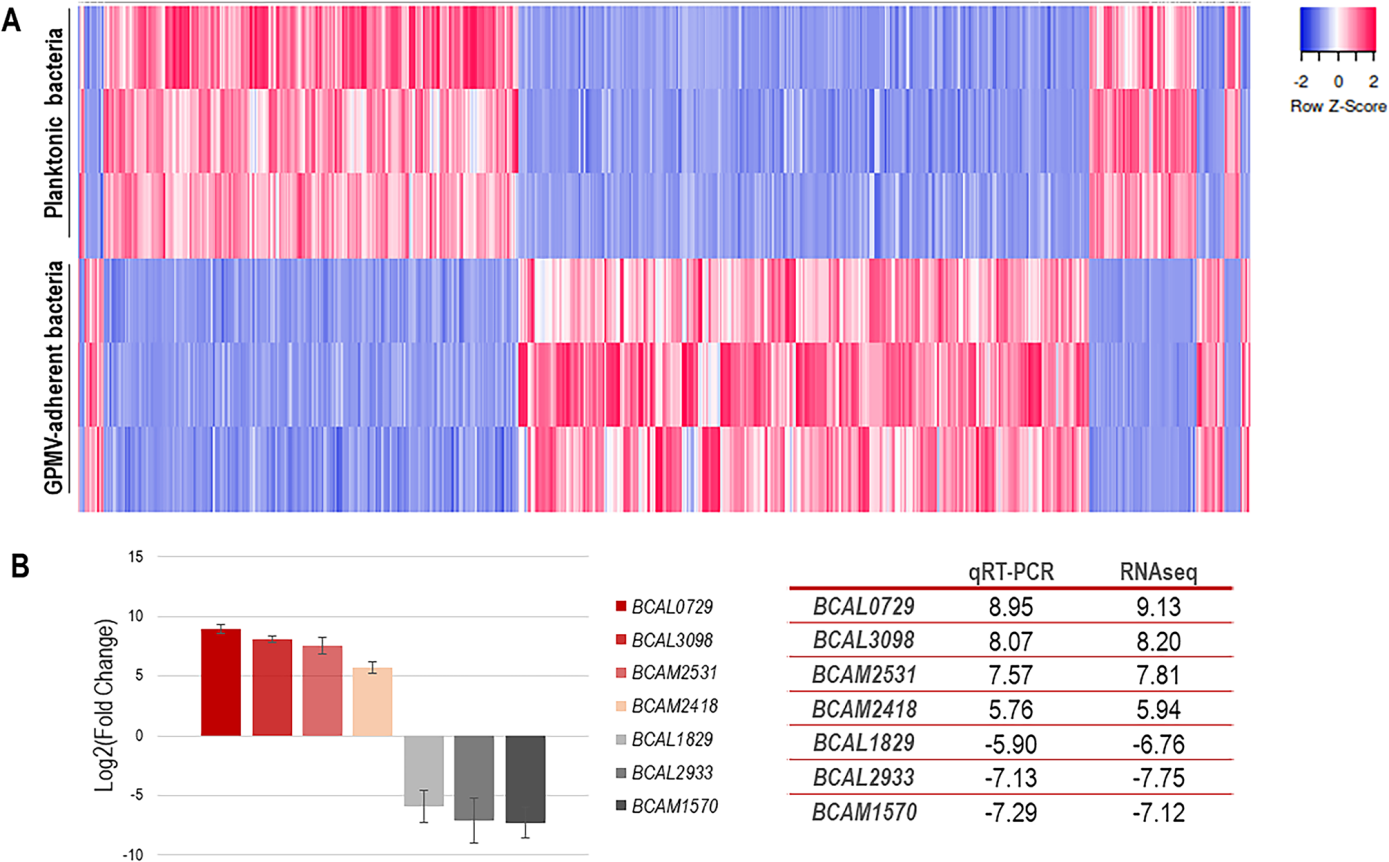
Heat map of *B. cenocepacia* K56-2 genes expression during adhesion to 16HBE14o- GPMVs (**A**). Colors from white to pink indicate upregulated cellular genes; colors from white to blue indicate downregulated cellular genes. Data from three replicas are represented. Heat maps were created using Heatmapper online platform (http://www.heatmapper.ca)^70^. Dataset validation by qRT-PCR (**B**). Transcription levels of *B. cenocepacia* K56-2 *BCAL0729*, *BCAL3098*, *BCAM2531*, *BCAM2418*, *BCAL1829*, *BCAL2933* and *BCAM1570* genes were obtained by qRT-PCR from bacteria adherent to 16HBE14o- GPMVs (30 min of contact). Expression levels are represented as Log2(Fold change) relative values in comparison to the expression levels of non-adherent bacteria. All the results are from three independent experiments, bars indicate SD (**P < 0.01; ****P < 0.0001).

To monitor alterations in the transcriptional profile of *B. cenocepacia* K56-2 after adhesion to bronchial cells-derived GPMVs (30 min), RNAseq was performed and the expression of the adherent (GPMV-attached) and non-adherent (planktonic control) bacteria were compared. Using a fold change cut off of ≥ 1.5 (adjusted *P*-value < 0.01), the obtained RNAseq dataset indicates a total of 926 genes which expression was altered upon bacteria-GPMV contact, that represents 12.7% of *B. cenocepacia* K56-2 coding genes. From those, 496 genes (53.6%) were up-regulated and 430 (46.4%) were down-regulated (Fig. 4A), indicating that the contact with GPMV had an impact in *B. cenocepacia* K56-2 gene expression. The obtained fold change values range from 560.205 (*BCAL0729*) to − 215.050 (*BCAL2933*).

To validate the dataset, qRT-PCR was performed using primers for seven genes, both up- and down-regulated (Fig. 4B). The high expression of *BCAL0729* (nitrogen regulatory protein P-II 1), *BCAL3098* (putative ABC transporter substrate-binding protein), *BCAM2531* (putative ABC transporter solute-binding protein) and *BCAM2418* (trimeric autotransporter adhesin); and the low expression levels of *BCAL1829* (putative outer membrane protein), *BCAL2933* (D-amino acid dehydrogenase small subunit) and *BCAM1570* (alcohol dehydrogenase) genes were confirmed using qPCR and the fold change values were comparable to the ones obtained for RNAseq (Fig. 4B).

### Genes involved in metabolic pathways and cellular information processing are highly altered upon *B. cenocepacia* K56-2 adhesion to 16HBE14o- GPMVs

**Figure 5:**
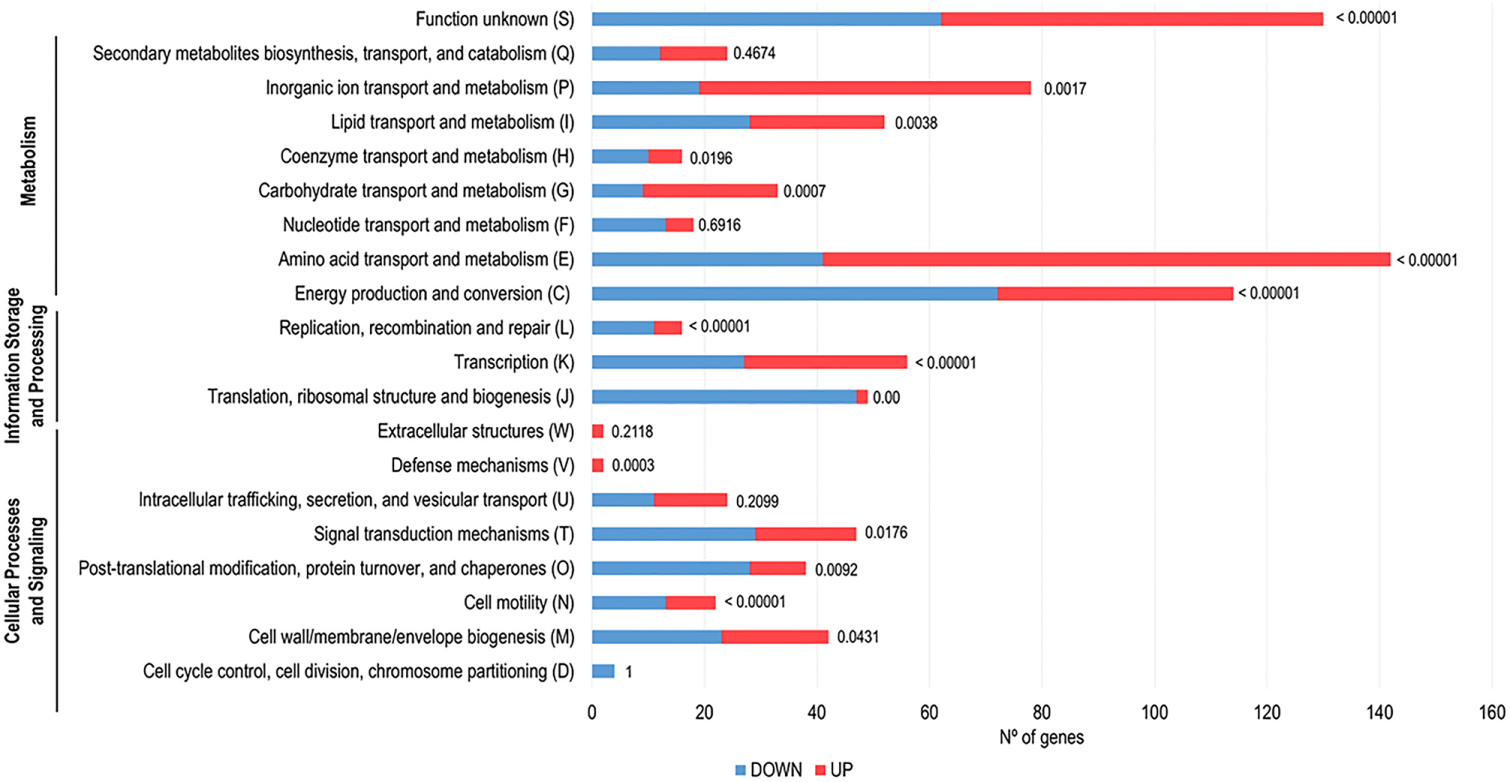
Clustering, based on biological function, of the genes found to be differently expressed upon *B. cenocepacia* K56-2 adhesion to 16HBE14o- GPMVs (30 min of contact). Down-regulated genes are represented by blue bars and up-regulated genes by pink bars. Biological function information was based on the information available in the Burkholderia Genome database and in the COG Database. The *P*-value of each cluster category is represented near the respective bar.

To evaluate functions of the differentially expressed transcripts the identified genes were analyzed using COG database (Fig. 5). The majority of the up-regulated genes (55.0%) were predicted to be involved in metabolism (transport and metabolism of amino acids (n = 101), inorganic ions (n = 59), carbohydrates (n = 24), lipids (n = 24), transport and metabolism of coenzymes (n = 6) and energy production (n = 42)). Around 14.3% of the highly expressed genes were involved in cellular processing and signaling (cell wall/membrane/envelope biogenesis (n = 19), signal transduction mechanisms (n = 18), post-translational modification, protein turnover and chaperones (n = 10), cell motility (n = 9) and defense mechanisms (n = 2)). 7.3% of the genes were corresponding to information storage and processing (transcription (n = 29) and replication, recombination, and repair (n = 5)).

Regarding down-regulated genes, 47.4% were predicted to be implicated in metabolism [energy production (n = 72), transport and metabolism of amino acids (n = 41), lipids (n = 28), inorganic ions (n = 19), nucleotides (n = 13), transport and metabolism of coenzymes (n = 10) and carbohydrates (n = 9)]. About 25.1% of the transcripts were predicted to participate in cellular processing and signaling (signal transduction mechanisms (n = 29), post-translational modification, protein turnover and chaperones (n = 28), cell wall/membrane/envelope biogenesis (n = 23) and cell motility (n = 13)). Approximately 19.8% of the genes were corresponding to information storage and processing [translation and ribosomal biogenesis (n = 47), transcription (n = 27) and replication, recombination, and repair (n = 11)]. Considering both up- and down-regulated group of genes, 23.4% and 23.7%, respectively, were grouped as poorly characterized.

When compared both datasets, it is notable that up-regulated genes are more likely to be involved in metabolic pathways, namely amino acid and inorganic ions transport and metabolism and energy production and conversion. The down-regulated genes seem to play an important role in, translation and ribosome biogenesis, transcription, post-translational modifications, and signal transduction mechanisms (Fig. 5).

### Pathway analysis of the differentially expressed genes revealed distinct putative functions

**Figure 6:**
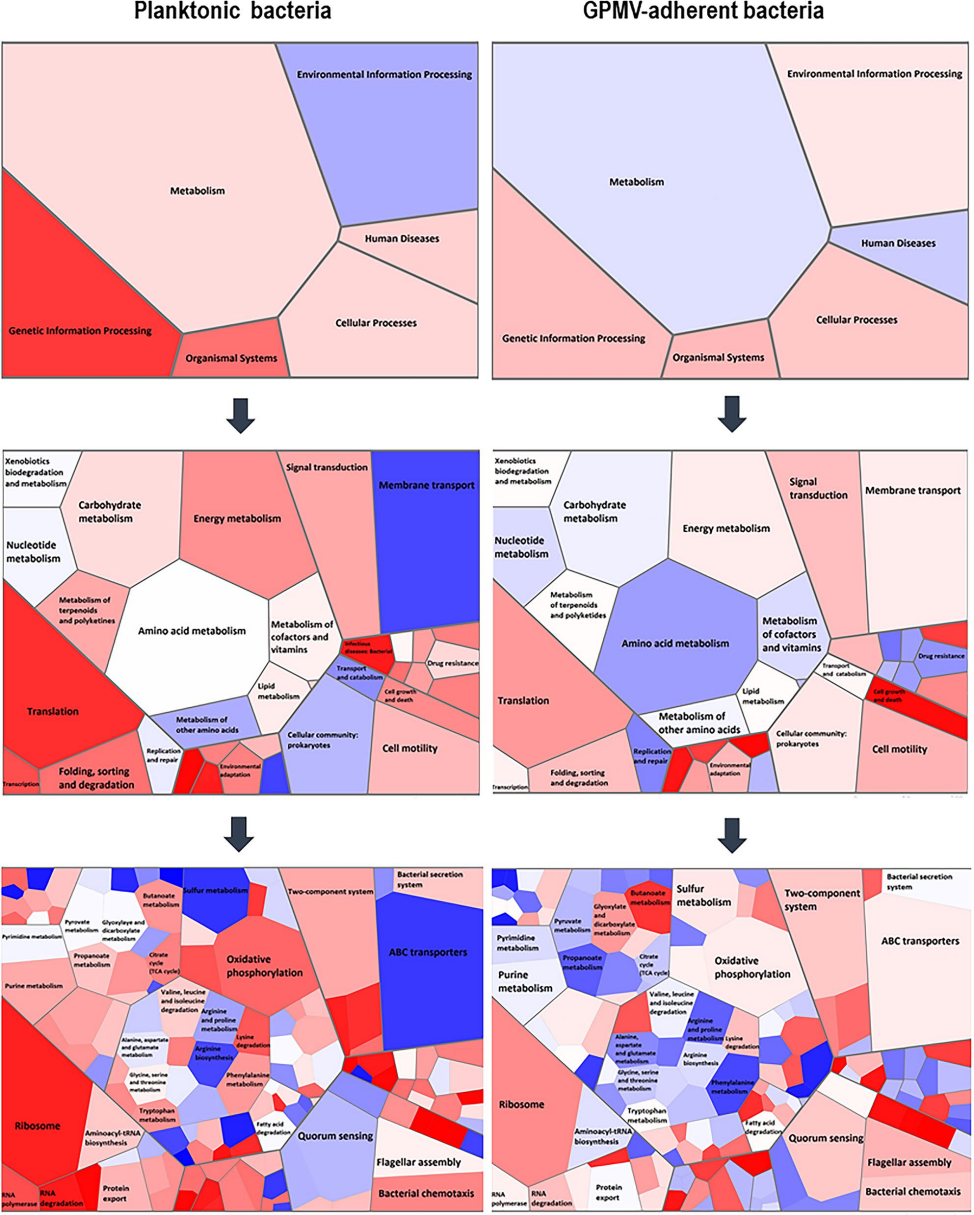
Evaluation of functional roles of the differentially expressed genes (*P* < 0.01; fold change greater or equal to 1.5 or less or equal to − 1.5), a KEGG pathway analysis was performed^25,26^. Statistically significant alterations are illustrated as Voronoi tessellations, created using Voronto mapper web service^27^. Voronoi maps integrate expression data and biological ontologies, allowing the evaluation the whole ontology and detection of changes on expression patterns inside the ontology. Each section represents a hierarchy level of ontology. The size of each cell represents the number of annotated genes identified for each ontology term. The final visualization represents three ontology levels for each dataset. Each term is represented by a polygon colored with its expression. Terms with a common ancestor are represented by adjacent polygon and surrounded by a wider line. Colors from white to red indicate overexpression; colors from white to blue indicate underexpression.

To go further in the evaluation of the functional roles of the differentially expressed genes, a KEGG pathway analysis was performed^25,26^. The statistically significant changes are illustrated as Voronoi tree maps in Fig. 6. To illustrate the statistically significant alterations (*P* < 0.01; fold change ≥ 1.5), Vononoi tessellations were created using Voronto mapper web service^27^. From the totality of the genes with altered expression, only 47.8% were KEGG annotated genes. It is noteworthy that most transcriptomic changes are concentrated on metabolism, environmental information processing, organismal systems, and genetic information processing pathways. Relating to GPMV-adherent bacteria, genes involved in processes like oxidative phosphorylation (energy metabolism), amino acid metabolism, TCA cycle and propanoate metabolism (carbohydrate metabolism) are down-regulated alongside with genes participating in translation, transcription, RNA-degradation and replication mechanisms. On contrary, genes that encode for membrane transport structures, like ABC transporters, are up regulated. The same seems to be the case for genes involved in sulfur metabolism (energy metabolism), quorum sensing and cell growth and death (Fig. 6).

The entries for enrichment analysis of KEGG pathways are resumed in Tables S1 and S2 (Supplementary data). Datasets for up- and down-regulated genes were analyzed using ShinyGO v0.61: Gene Ontology Enrichment Analysis web service^28^, and the most significant enriched pathways were selected and seem to confirm the results obtained in Vononoi tessellations. The majority of the up-regulated pathways were found to be involved in ABC transporters and metabolic functions, in particular sulfur, taurine and hypotaurine metabolism, nitrogen metabolism, glyoxylate and dicarboxylate metabolism and fatty acid degradation and metabolism as possible alternative sources of energy. Concerning ABC transporters, 81 genes (30.7%) were found to be upregulated, including genes involved in transport of potassium, glutamate/aspartate, amino acids, sugars, like ribose and maltose, sulfate, and nitrogen. In some cases, the transcription of an entire gene cluster is observed, such as the Kdp system (*BCAL2379-2383*—*kdpA-E*), responsible for potassium transport, Glt genes (*BCAL3356-3358*—*gltI-K*) (glutamate/aspartate transport), genes involved in branched-chain amino acids import (*BCAL0015-0019*) and genes involved in sulfate uptake (*BCAL1652-1657*—*sbp, cysT, cysW, cysA, ssuR*). Also, genes involved in two-component systems (signal transduction) and in the biosynthesis of amino acids are also up-regulated. Additionally, several genes implicated in flagellar assembly (17.5%) were induced during *B. cenocepacia* adhesion alongside with 6 genes involved in bacterial chemotaxis, namely flagellar motor switch protein coding genes—*fliG* and *fliM*; and *BCAL1657* (putative ribose transport system) and *BCAM0766* (D-ribose-binding periplasmic protein precursor) which appear to be associated with ribose-related pathways (Table S1).

In contrast, several metabolic pathways are down-regulated, namely oxidative phosphorylation, carbon metabolism, citrate cycle (TCA cycle), purine metabolism, biosynthesis of secondary metabolites, pyruvate metabolism and glycolysis and gluconeogenesis. Regarding oxidative phosphorylation, 60.8% of the genes were repressed, including genes that encode F0F1 ATP synthase subunits (*BCAL0032-0037*—*atpACDFGH*), cytochrome C oxidase proteins (*BCAL0750, 0752, 0754*), cytochrome D (*BCAL0784-0785, cydAB*) and O (*BCAL2141-2144, cyoA-D*) ubiquinol oxidase subunits and succinate dehydrogenase subunits (*BCAM0969-0970*, *sdhAB*). Moreover, type I NADH dehydrogenase 14 subunit genes, that are organized in the *nuo* locus appear to be down regulated as well (*BCAL2331-2344*, *nuoA-N*). Interestingly, *ndh* gene (*BCAM0166*) that encodes for type II NADH dehydrogenase was found to be up regulated. Cellular processes like transcription and translation seem to be affected upon bacterial-GPMV adhesion too. The expression of three (*rpoA*, *rpoB* and *rpoC*) out of the four RNA-polymerase subunit genes appear to be down-regulated as well as genes involved in aminoacyl-tRNA biosynthesis, namely aspartyl-, glutaminyl-, valyl-, phenylalanyl-, histidyl- and tryptophanyl-tRNA synthetases (*aspS*, *glnS*, *valS*, *pheT*, *hisS* and *trpS*). Moreover, 49.1% of ribosomal proteins were found to be downregulated, being that 11 genes encode for 30S ribosomal subunit proteins and 17 genes for 50S subunit, which indicates an impressive reduction in ribosomal production and activity (Table S2). Bacterial chemotaxis also seems to be altered upon *B. cenocepacia* K56-2 early contacts with host-cell membranes. Despite some genes that appear to be up regulated, 32.5% of the genes involved in chemotaxis pathways were found to be down regulated, including 8 genes belonging to the chemotaxis gene cluster—*BCAL0126* (*motA*), *BCAL0129-0135* (*cheA*, *cheW*, *tar*, *cheR*, *cheD*, *cheB1* and *cheY*). Aside from that, 4 methyl-accepting chemotaxis proteins were also repressed—*BCAL0762*, *BCAM1503*, *BCAM1572* and *BCAM1804*, and *aer* (*BCAM2564*) a putative aerotaxis receptor that is known to sense environmental oxygen levels. Apart from that, expression of genes related to protein export systems, namely the SEC dependent pathway, was also repressed. Both post- and co-translational translocation seems to be affected. In the first category are included genes that encode for translocation channel and related proteins, like *BCAL0254* (*secY*), *BCAL0742* (*secB*), *BCAL3307* (*secF*) and *BCAL3433* (*secA*). In the second one, *BCAL3453* (*ffh*) that encodes for a signal recognition particle protein involved in targeting and insertion of nascent membrane proteins into the cytoplasmic membrane (Table S2).

### *B. cenocepacia* K56-2 modulates adhesion and invasion factors expression upon GPMV-adhesion

**Figure 7:**
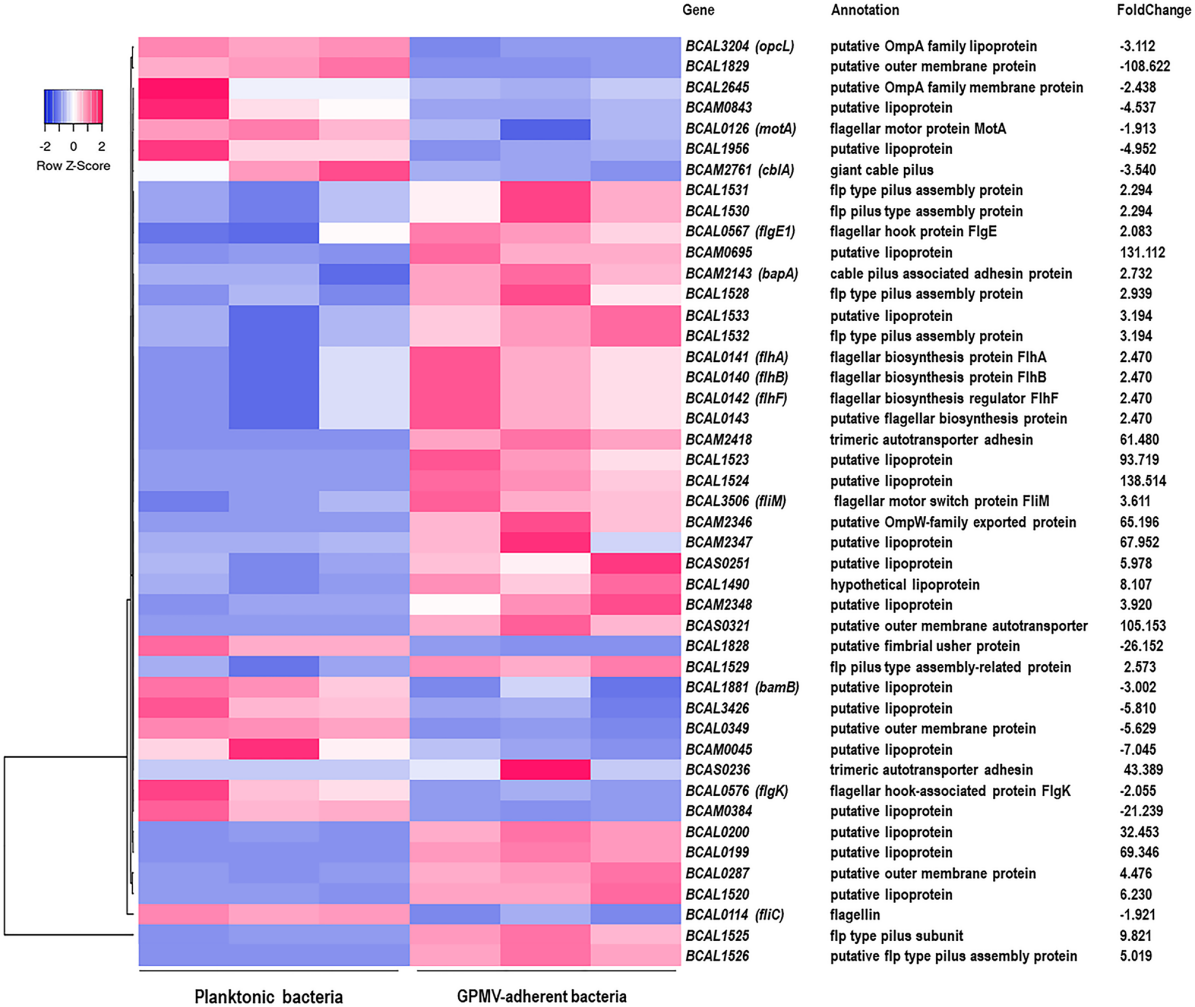
Heat map of *B. cenocepacia* adhesion and invasion related genes expression during adhesion to 16HBE14o- GPMVs. Colors from white to pink indicate upregulated cellular genes; colors from white to blue indicate downregulated cellular genes. Heat maps were created using Heatmapper online platform (http://www.heatmapper.ca)^70^. Several genes that promote *B. cenocepacia* K56-2 interaction with the host cell, including genes that encode for adhesins, outer membrane proteins, lipoproteins and proteins involved in pilus and flagella assembly were differentially regulated upon GPMV-adhesion.

Several genes that promote *B. cenocepacia* K56-2 interaction with the host cell, including the ones that encode for adhesins, outer membrane proteins, lipoproteins and proteins involved in pilus and flagella assembly and function were differentially regulated upon GPMV-adhesion (Fig. 7). The altered expression of these genes may represent a prompt response to the sensing of the host-membrane surface with the consequent increase of bacterial adhesion. Data shown numerous pilus associated genes with an enhanced expression after 30 min of GPMV-adhesion, namely flp type pilus subunit *BCAL1525* and flp type pilus assembly protein coding genes—*BCAL1526, BCAL1528*, *BCAL1529*, *BCAL1530*, *BCAL1531* and *BCAL1532*. Nearby putative lipoproteins *BCAL1533*, *BCAL1520*, *BCAL1523* and *BCAL1524* were also found to be highly induced. Apart from that, it is noteworthy that although *BCAM2461* (*cblA*—giant cable pilus) coding gene is repressed, *BCAM2143* (*bapA*—cable pilus associated adhesin) appears to be up-regulated, indicating a possible differential role played by both proteins during the early stages of bacteria-host cell interaction. Considering flagella related genes, nine appear to have an altered expression during *B. cenocepacia*-GPMV adhesion. Among them, flagellar biosynthesis proteins—*BCAL0141* (*flhA*), *BCAL0140* (*flhB*) and *BCAL0143* (putative), *BCAL3506* (*fliM*—flagellar motor switch) and *BCAL0567* (*flpE1*—flagellar hook protein) were found to be upregulated genes. On the other hand, *BCAL0126* (*motA*—flagellar motor protein), *BCAL0576* (*flgK*—flagellar hook-associated protein) and *BCAL0114* (*fliC*—flagellin) were found to be downregulated during adhesion.

Additionally, a set of lipoproteins (n = 17), outer membrane proteins (n = 6) and several adhesins coding genes were differentially expressed throughout adhesion. Aside from cable pilus associated adhesin, trimeric autotransporter adhesins—*BCAM2418* and *BCAS0236*, and *BCAS0321*, a putative outer membrane autotransporter, were highly overexpressed with foldchange values of 61.48, 43.389 and 105.153, respectively. Moreover, despite belonging to different autotransporter classes (type Vc and Va), BCAM2418, BCAS0236 and BCAS0321 share some common features like the presence of β-barrel membrane anchor domains and an elevated number of amino acid residues—2750, 1496 and 4234, respectively. Nonetheless, *BCAM1881* that encodes for a putative BamB lipoprotein, that is known to be part of the BAM (β-Barrel Assembly Machinery) complex which is involved in the assembly and insertion of β-barrel proteins into the outer membrane, appears to be repressed upon adhesion^29^. The size of these autotransporter adhesins may suggest that the bacterial cell must endure an energetic effort to overproduce them and that a BAM alternative outer membrane protein assembly complex, like TAM (Translocation and Assembly Module), could be involved in the translocation of these proteins across the membrane^30^.

## Discussion

The first contact between a bacterium and a host-cell surface is the crucial step for the development of an infection, leading to physiological alterations in both interacting cells^14,16^. For bacteria, these alterations allow them to change and adapt to the new environment and cause an enhanced virulence fitness that may induce further invasion of host cells and destruction of epithelial tissue^16,31^. The study of bacteria initial interactions with host cell-membranes is challenging. The exact contribution of the cell membrane physical contacts in the bacterial transcriptomic shift is hard to achieve since the surrounding environment of the host epithelium is a complex mixture of cell-derived molecules, cellular-trafficking structures, and other type of biological compounds. GPMVs derive from the cell plasma membrane and offer a close approximation to it, which make them a suitable model for a potential host membrane surrogate^32^.

In this work we produced GPMVs from a bronchial epithelial cell line and used them for the first time as target for *B. cenocepacia* K56-2 adhesion. Further studies were also performed to understand the transcriptomic alterations caused by such contacts in the bacterial adaptation during the early host membrane contacts. Vesiculation of 16HBE14o- cell line was chemically induced and the resulting GPMVs shown to be constituted not only by plasmatic membrane structures but also by host transmembrane proteins. Western Blot assays demonstrated the presence of the same proteins in both native-cells and vesicles, indicating that, as shown by others, GPMVs share functional and structural similarities with the cells that they are originated from^32,33^. Nonetheless, it is important no notice that this model also presents some limitations like the covalent modifications that the vesiculation agents (PFA and DTT) can induce on the membrane and proteins of GPMVs^23,32^. Despite being the most approximated model of cell membranes, GPMVs also lack some of the native bilayer asymmetry, phase separation and have higher levels of cholesterol and lower amounts of polyunsaturated fatty acids, as compared with cell membranes^34,35^. Moreover, GPMVs are an inactive model, meaning that the bilayer membrane is in a state of thermodynamic equilibrium while the biological plasma membrane is highly dynamic and out-of-equilibrium. The cellular plasma membrane is also constantly being modified by vesicle trafficking and interactions with the cytoskeleton molecules, and these exchanges do not occur in the formed plasma vesicles^32,34,36^.

In recent years, several findings have been made concerning the binding, interaction and penetration of viral proteins and bacterial toxins using GPMVs as membrane models^37–40^. To the best of our knowledge, this is the first work to introduce the usage of giant vesicles in the study of host-membrane interactions with microorganisms as a whole. It was showed that *B. cenocepacia* can adhere, in a similar manner, to both GPMVs and cells. Such interaction appears to occur in a dynamic and sequential way, suggesting that processes like invasion and membrane penetration might be starting to be established. The adaptation of *B. cenocepacia* to those stages of contact may have an impact in the transcriptomic profiles obtained for adherent bacteria when compared to planktonic ones. Several transcriptional studies designed to examine gene expression of *B. cenocepacia* in different environments helped the identification of new genes that proved to be important in virulence^22,41,42^. In this work we completed a transcriptional profiling at the whole genome level of *B. cenocepacia* K56-2 upon the early contacts with the host-cell membrane, using GPMVs as cellular surrogate, and compared to planktonic laboratory-grown bacteria.

Notably, we observed that the early interaction of *B. cenocepacia* K56-2 with the bronchial cell membrane led to an alteration of the bacterial metabolic pathways, like the downregulation of the central metabolism as a way to adapt to the new host environment. These include oxidative phosphorylation, carbon metabolism, TCA cycle and glycolysis and gluconeogenesis. In contrast, genes involved in sulfur and nitrogen metabolism, glyoxylate metabolism, CoA biosynthesis and fatty acid catabolism are upregulated during bacterial adhesion to GPMVs. The interaction with the host seems to initiate a modulation of key bacterial systems that supports a progressive adaptation in order to exploit the nutrients available in the host during the infection cycle. A representation of the altered metabolic regime due to the evolving substrate availability is the induction of a large number of genes encoding for transport machineries, including those involved in the uptake of sugars, amino acids, potassium, sulfate and nitrogen. The adaptation to a different lifestyle by shifting major metabolic pathways was reported in other pathogenic bacteria^21,22,43^. The transcriptional response of intracellular *B. cenocepacia* was also studied elsewhere, and obtained results support the assumption that a metabolic change to the new niche plays a major role in the bacterial survival^44^. Nevertheless, the occurrence of this type of adjustment in the early stages of bacteria-host interaction is still poorly documented. It is possible that *B. cenocepacia* adaptation to the host environment occurs after the first physical contacts to the host surface rather than being a consequence of a chronical infection state^20,22^. The induction of sulfur-metabolism is one example of *B. cenocepacia* adaptation to the host. The link between sulfur metabolism and virulence has been reported for several bacterial pathogens^45^ and as a long-term adaptation during *B. cenocepacia* host colonization^20^. The overexpression of sulfate starvation-induced genes involved in the cysteine biosynthesis pathway like the *cysTWA* (sulfate transporter operon), *cysI* and *cysH* (sulfate activation and reduction operon), *ssuDCB* (transport and desulfonation of aliphatic sulfonates) and three taurine deoxygenases (*tauD2a*, *tauD2b* and *tauD3*), responsible for the desulfonation of taurine, was reported in this work. The assimilation of sulfur from inorganic sulfate and other alternative sources seems to be a rapid response of *B. cenocepacia* to the early interactions with the host^45,46^. Interestingly, despite taurine being a non-essential amino acid commonly found in humans as source of sulfur, many sulfonates are known to be present in mucins and in the surface of epithelial lung cells, which could explain the prompt metabolic shift^45–47^.

Limited oxygen conditions are a typical feature of the lungs of CF patients, and it is also found to be a characteristic of the host-cells interior^4,48,49^. The decrease in the expression of genes related to oxidative phosphorylation was registered upon *B. cenocepacia* adhesion to GPMVs, namely genes encoding for several complexes of the electron transport chain—NADH-dehydrogenase I (NDH-1), cytochrome O, cytochrome D, cytochrome C oxidase and ATP synthase. On the other hand, NADH dehydrogenase II (NDH-2) and cytochrome C reductase complex subunits were found to be upregulated. The downregulation of genes encoding several cytochrome subunits was also observed as a long-term adaptation to chronic infection in CF airways, indicating that *B. cenocepacia* is able to alter its metabolism to survive under microaerophilic conditions or even temporary anoxia^20^. Schwab and colleagues (2014) suggested that in the context of CF hypoxic environment, *B. cenocepacia* gain energy by fermentative processes rather than anaerobic respiration^50^. Opposite to that, other *Burkholderia* species like *B. thailandensis* and *B. pseudomallei* were found to be able to adopt an anaerobic metabolism through the increase of anaerobic nitrate respiration under conditions that mimic in vivo infections^51^. The upregulation of *nirB* (putative nitrite reductase) and several genes involved in nitrate uptake obtained in GPMV-adherence condition may suggest that anaerobic nitrate respiration could also be used by *B. cenocepacia* K56-2. Although deeper studies are in need to enlighten this hypothesis, the repression of genes involved in aerobic electron transfer suggest that *B. cenocepacia* may adjust its ATP generation processes soon after the first interactions with the membrane of host cells^49^.

*B. cenocepacia* K56-2 cellular processes were found to be highly disturbed during bacterial adhesion to host membranes. The repression of genes encoding proteins related to transcription (RNA-polymerase subunits *rpoA*, *rpoB* and *rpoC*), translation (aminoacyl-tRNA synthetases *aspS*, *glnS*, *valS*, *pheT*, *hisS* and *trpS* and 30S and 50S ribosomal subunit genes) and protein export (sec dependent pathway *secY*, *secB*, *secF*, *secA* and *ffh*) was reported. The repression of these major cellular processes could be occurring as a preparation for the bacterial intracellular lifestyle, since *B. cenocepacia* can survive intracellularly with minimal or no replication in order to evade host defenses and to establish chronic infections^52,53^. *B. cenocepacia* ability to invade and persist inside host-cells has been well documented, and several studies have indicated that engulfed bacteria undergo intracellular replication at reduced levels over 24-48 h post-infection^54–56^. Although in a state similar to stationary phase, intracellular *B. cenocepacia* were found to remain metabolically active^53,54,57^. The obtained results reinforce the idea that the sensing and early contacts with the host-cell surface may induce transcriptional alterations that favor the intracellular regime of *B. cenocepacia*.

The bacteria capacity to move towards or away of a specific environmental signal is known as chemotaxis and is based on the action of several chemosensory pathways. The che pathway is required for flagellum-mediated chemotaxis and it is initiated through the recognition of a signal that created a stimulus responsible for modulating the phosphorylation of the response regulator that ultimately binds to the flagellar motor causing the flagellum rotation^58–60^. The majority of the genes encoding for proteins involved in the che chemotaxis signaling pathway were found to be suppressed, including *cheA* (two-component sensor kinase), *cheW* (adaptor protein), *tar* (methyl-accepting protein), *cheR* (methyltransferase), *cheD* (chemoreceptor glutamine deamidase), *cheB1* (chemotaxis-specific methylesterase) and *cheY* (response regulator). Apart from that, *fliC* (flagellin), *flgK* (flagellar hook-associated protein) *motA* that encodes for a flagellar motor protein and four different methyl-accepting chemotaxis proteins, responsible for sensing the environmental stimuli, were also down-regulated upon adhesion. Nevertheless, several genes encoding for flagellar biosynthesis and assembly proteins were induced in GPMV-adherent bacteria—*flhB*, *flhA*, *fliH*, *fliG*, *fliF*, *flgF*, *flgE1* and *fliM*, which may be seen as conflicting results. Flagella and motility represent important virulence features since the loss of motility caused reduced invasiveness of epithelial cells^5^. Nevertheless, previous works indicated that expression of flagellar- and chemotaxis-associated genes and motility were reduced in *B. cenocepacia* strains isolated from CF patients (ET12 lineage)^61^. The obtained data imply that interaction with host-cell membranes in the early stages of *B. cenocepacia* infection may lead to a disruption of the bacteria movement in response to a chemical gradient (chemotaxis), but to an increase in the assembly of the hook and basal structures of the flagella. In *Salmonella*, the flagellar, motility and chemotaxis genes are organized in a regulon and they are arranged into three hierarchical classes. The early operon is constituted by *flhDC* genes that control the transcription of more than 30 middle genes (class 2) that are required for the structure and assembly of the hook and basal body, including the genes induced in this work. Finally, class 3 genes encode for proteins like flagellin, hook-associated proteins and chemotaxis systems (che pathway)^60,62^. It is possible that the contact between *B. cenocepacia* K56-2 and the surface of epithelial cells could trigger this type of targeted transcription that ultimately leads to the full expression of flagellar and chemotaxis genes. This time-dependent sequential gene expression could limit the effectiveness of a motile-flagella and target its formation when an active invasion of host-cells is required.

Despite flagella, other membrane appendages, known for their role in bacterial adherence and virulence, were found to be overexpressed during *B. cenocepacia* adhesion to 16HBE14o- derived GPMVs. Genes belonging to the genomic locus *BCAL1520-1537* that encodes components of Flp type pilus, *bapA* cable pilus associated adhesin, *BCAS0321* outer membrane autotransporter adhesin, and two trimeric autotransporter adhesins (TAAs) —*BCAM2418* and *BCAS0236* are examples. The expression of pilus structures has been extensively associated with bacterial adherence, motility, and host-cell invasion^63,64^. Nevertheless, the lack of Flp pilus expression seems to be a predominant characteristic in outbreak isolates and during an established *B. cenocepacia* infection^22,61^. The obtained data suggests that despite its absence during chronical infections, Flp pilus appear to be important in the early stages of bacteria-host confrontations. On the other hand, the expression of *cblA* (major subunit of giant cable pilus) is repressed during *B. cenocepacia*-GPMVs adhesion, indicating non-essential role for this type of pilus structure. Moreover, BapA cable pilus associated adhesin encoding gene was found to be induced, revealing that both cable pilus and its associated adhesin may play different roles during *B. cenocepacia* adhesion to host cells. Several studies shown that both Cbl pili and BapA are necessary for the optimal binding to cytokeratin 13, a receptor on the membrane of the host-cell^8,63^. Nevertheless, when Cbl pili is absent *B. cenocepacia* remains able to adhere, indicating the importance of BapA in that process. Also, adhesin-mediated binding to cytokeratin 13 seems to be absolutely necessary for subsequent invasion and transmigration across the epithelium^10,63,65^. *B. cenocepacia* TAAs have been studied in detail during the past years, and are known to be multifunctional proteins involved in many virulence related traits like biofilm formation, motility, adhesion and invasion of host cells^9,11,13,66,67^. The expression of BCAM2418 and BCAS0236 TAA encoding genes during the early stages of *B. cenocepacia* infection was demonstrated in a recent work^12^. The overexpression of *BCAM2418* transcripts shown to be variable over time, reaching a maximum after 30 min of *B. cenocepacia* adhesion to bronchial epithelial cells. Also, the bacterial interaction with specific host-cell receptors, namely O-linked glycosylated proteins, was reported to be the trigger for the increased expression of BCAM2418 TAA^12^. The obtained data in this work seems to support those observations as BCAM2418 TAA expression increased after bacterial interaction with the host-cell membranes. Those results not only suggest an important role played by this TAA in the early stages of infection, but also support the experimental applicability of GPMVs as a host-cell surrogates in the study of the initial crosstalk between bacterial pathogens and their hosts.

**Figure 8:**
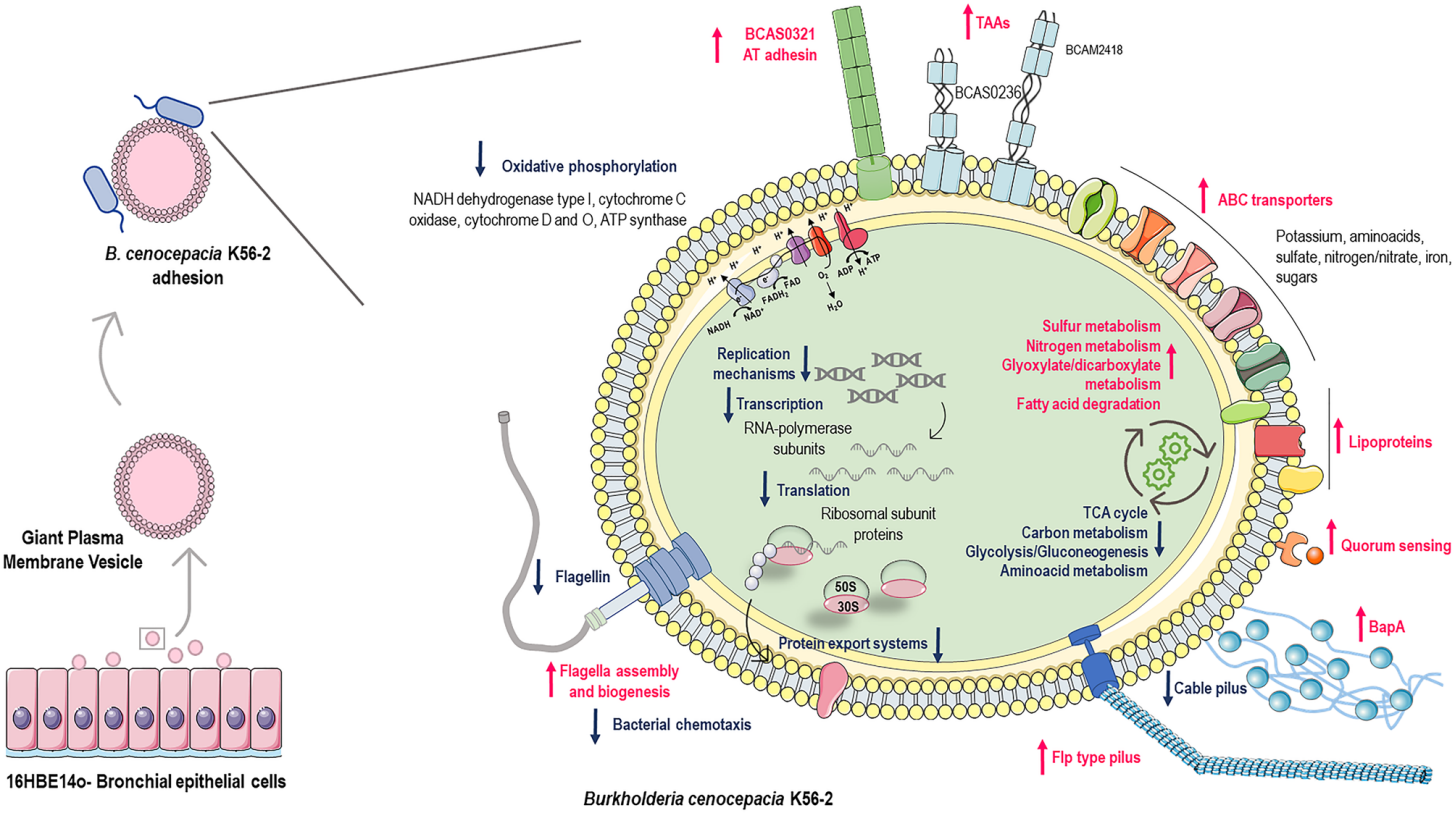
*B. cenocepacia* K56-2 transcriptomic adaptation upon contact with the surface of the host cell. 16HBE14o- bronchial epithelial cell line was used to produce GPMVs. Adhesion between *B. cenocepacia* K56-2 and GPMVs was performed during 30 min, RNA from adherent bacteria was recovered and RNAseq was performed. Major alterations in several important pathways were registered, such as central metabolism, oxidative phosphorylation, replication, transcription, and translation; and in the expression of adhesive structures—flagella, pili, lipoproteins and adhesins. Processes and structures upregulated are represented in pink and downregulated in blue. The figure was created using materials from SMART- Servier medical art (https://smart.servier.com/).

In summary, in this work we produced GPMVs from a bronchial epithelial cell line and used them for the first time as a cell-like alternative to explore *B. cenocepacia* interaction with host-cell surface (Fig. 8). The perceiving of the host membranes by the pathogenic bacteria leads a transcriptional shift that cause a cascade of metabolic and physiological adaptations to the host specific environment. Our results demonstrated that almost 1000 genes had their transcription changed after *B. cenocepacia* physical contact with cell membranes. Many of these genes encode proteins related with central metabolic pathways, transport systems, cellular processes, and virulence traits (Fig. 8). The understanding of the changes in gene expression that occur in the early steps of an infection cycle could uncover the first mechanisms that a pathogenic bacterium uses to invade and subvert the host cell, providing new strategies to limit *B. cenocepacia* lung infections. Further research including construction of mutant strains, is necessary to identify potential novel virulence-associated genes essential for the pathogenesis of *B. cenocepacia* during host cell initial crosstalk.

## Materials and methods

### Bacterial strain and growth conditions

*Burkholderia cenocepacia* clinical isolate K56-2 was kindly provided by J. J. LiPuma (University of Michigan). Bacteria were grown in supplied Luria–Bertani (LB) broth (NZYTech) at 37 °C with orbital agitation (250 rpm). For functional studies, bacteria from a fresh overnight culture were grown (initial OD_640_ 0.1) at 37 °C with orbital agitation at 250 rpm, for 6 h until reaching a mid-exponential phase of growth.

### Cell line and cell culture

16HBE14o—a human bronchial epithelial cell line was used (made available by Dr. Dieter Gruenert, Pacific Medical Center Research Institute, San Francisco, CA)^68^. During standard procedures, cells were maintained in a humidified atmosphere at 37 °C with 5% CO_2_, in minimum essential medium with Earle’s salt (MEM) supplemented with 10% fetal bovine serum (FBS), 0.292 g/L L-glutamine, and penicillin–streptomycin (100 U/mL).

### Giant plasma membrane vesicles (GPMVs) production

Giant Plasma Membrane Vesicles (GPMVs) production and isolation protocol was performed as previously described^32^ with some alterations. 16HBE14o- cells were seeded and grown until reaching a confluence of more than 70%. Cells were washed twice with 100 µL/cm^2^ of GPMV buffer (10 mM HEPES, 150 mM NaCl, 2 mM CaCl_2_, pH7.4). GPMV reagent containing the vesiculation agents (25 mM paraformaldehyde (PFA), 2 mM DTT in GPMV buffer) was applied to the cells in the same 100 µL/cm^2^ ratio. The cells were incubated at 37 °C with low agitation (100 rpm), during 1 h. The GPMV-enriched supernatant was transferred to a centrifuge tube by decantation. To pelleting the cellular debris, the GPMV suspension was centrifuged 10 min at 100×g. The resulting supernatant was carefully collected by pipetting. To perform adhesion assays the GPMVs were concentrated and the vesiculation agents in the suspension were removed by ultrafiltration using an Amicon Ultra-15 Centrifugal Filter Unit with a 100 kDa molecular cut-off. The GPMV suspension were applied to the filter unit (15 mL at a time), and centrifuged 15 min, at 3500×g leaving at least 3 mL of final volume in the tube. Ten volumes of GPMV buffer were applied to wash the vesicles and remove PFA and DTT from the suspension. The dialyzed vesicles were recovered from the filter unit and added to a culture plate coated with a solution of 0.1% (w/v) of poly-L-lysine (Sigma-Aldrich). The plates were then centrifuged 5 min at 750×*g* and incubated overnight at 37 °C, with 5% CO_2,_ to promote GPMVs adhesion to the coated surface. The starting cell culture should be prepared in a culture area 8 × higher than the surface where the purified GPMVs will be applied, in order to achieve a confluent layer of vesicles.

### Western blot analysis

For Western blot analysis, GMPV and 16HBE14o- cellular extracts were prepared. Samples were washed twice with PBS (phosphate buffer saline) and a mixture of CLB buffer (1% (v/v) Triton X-100, 1% (v/v) NP-40, in PBS pH 7.4) and protease inhibitors was added to the samples and let to incubate for 10 min at 4 °C. GPMV and cellular monolayer were scrapped, vortexed 10 s three times and centrifuged at 14 000 rpm, 10 min at 4 °C. The supernatant was recovered and quantified. A volume of extract corresponding to 30 µg of the total protein was mixed with Laemmli buffer with 5% (v/v) 2-β-mercaptoethanol and 5% (v/v) bromophenol blue and stored at − 20 °C. Both samples were boiled for 10 min for protein denaturation and separated by 10% SDS-PAGE. Proteins were transferred onto nitrocellulose membranes by using a Trans-Blot Turbo transfer system (Bio-Rad). Membranes were blocked with 5% (w/v) nonfat dry milk in PBS containing 0.5% (v/v) Tween 20 (PBS-T) for 1 h, incubated with anti-caveolin-1 (diluted 1:1000), anti-flotolin-1 (diluted 1:500), anti-FGFR-1 (diluted 1:250) and anti-TNFR (diluted 1:1000) antibodies, overnight at 4 °C. Membranes were washed three times for 5 min with PBS-T and were then incubated with a secondary antibody (diluted 1:2000), conjugated to horseradish peroxidase (Santa Cruz), for 1 h. Proteins were detected by the addition of ECL reagent as a substrate and chemiluminescence was captured by Fusion Solo (Viber Lourmat) equipment and exposure time was optimized for each target, ranging from 1 to 10 min.

### *B. cenocepacia *adhesion to 16HBE14o- cells and derived GPMVs

Adhesion assays were performed in polystyrene 24-well plates either on GPMVs derived from 16HBE14o cells and living 16HBE14o- cells as described previously^11^. Cells (5 × 10^5^ cells/well) were seeded one day preceding the infection in supplemented medium. Before adhesion, cells were washed with PBS and maintained in simple MEM medium without supplements. *B. cenocepacia* inoculum was used to infect host cells at a multiplicity of infection (MOI) of 50:1. For GPMVs adhesion, a confluent monolayer was prepared in poly-L-lysine coated 24-well plates in GPMV buffer, on day prior to adhesion. GPMVs monolayers were washed twice with GPMV buffer and maintained in the same buffer. Adhesion was performed with 5 × 10^7^ CFU per well of confluent GPMVs. The plates were then centrifuged 5 min at 750×g and incubated at 37 °C in 5% CO_2_ for 30 min to allow bacterial adherence. Cells were then washed three times with PBS and GPMVs with GPMV buffer and disrupted with lysis buffer (10 mM EDTA, 0.25% Triton X-100) for 30 min at room temperature. The adhered bacteria were quantified by plating of the produced lysates.

### Scanning electron microscopy (SEM) imaging

Samples of *B. cenocepacia* K56-2, 16HBE14o- cells and GPMVs were visualized by SEM. Confluent monolayers of epithelial cells and GPMVs were prepared on a glass coverslip coated with poly-l-lysine on a 24-well plate a day before the assay, as previously described. *B. cenocepacia* overnight inoculum was used. A bacterial suspension of 5 × 10^7^ CFUs was added to a coated coverslip and to cellular and GPMVs monolayers. The plates were then centrifuged 5 min at 750×g and incubated at 37 °C in 5% CO_2_ for 30 min to allow bacterial adherence. Samples were washed three times with GPMV buffer or PBS and fixed with a 2% (v/v) PFA, 2.5% (v/v) glutaraldehyde solution for 30 min at 25 °C. Samples were dehydrated with 70% (v/v) ethanol for 10 min, 95% (v/v) ethanol for 10 min, and finally absolute ethanol for 20 min. After complete air-drying, samples were mounted on a carbon conductive adhesive tape followed by gold–palladium coating (Polaron E-5100). Scanning electron microscopy (SEM) images were taken using a field-emission-gun scanning electron microscopy (FEG-SEM) Hitachi S4100 microscope operating at 20 kV with a sample-to-objective working distance of 15 mm.

### Total RNA extraction

Total RNA was extracted from GPMV-adherent bacteria after 30 min of primary contact. Purified GPMVs were deposited on a coated culture plate (Ø 100 mm) 24 h prior the adhesion experiment. Each experiment was performed with 1.5 × 10^9^ CFU of *B. cenocepacia* K56-2 per plate of 100% confluent GPMVs. Following the bacterial inoculation, the plates were subjected to centrifugation (5 min at 750×g) and then incubated at 37 °C in 5% CO_2_ for 30 min. After finishing this period of time, the supernatant was carefully removed and the GPMVs monolayer was subjected to three consecutive washing steps with GPMV buffer. GPMVs and adherent bacteria were then recovered with a cell scraper, centrifuged at 9000 rpm for 3 min and the resulting pellet resuspended in TE buffer. Lysozyme and proteinase K (Qiagen) were used to obtain bacterial lysates. Total RNA was purified from bacterial lysate using RNeasy mini kit (Qiagen), following manufacturer’s instructions. RNA was treated with RNase-free DNA digestion kit (Qiagen) in column for 1 h at room temperature, to prevent DNA contamination in RT-PCR. Whenever necessary, a second step of RNA purification was performed (RNase-free DNA digestion kit) using 1 µL DNase for 1.5 µg of RNA to be treated, overnight at 37 °C, followed by inactivation for 5 min at 65 °C.

### Reverse transcription PCR and real-time PCR

cDNA was produced from total RNA using Taqman Kit (Applied Biosystems) and then analyzed with Power SYBR Green Master Mix (Applied Biosystems). Expression of *BCAM02418*, *BCAM0729*, *BCAL1829*, *BCAL293*, *BCAL3098*, *BCAM1570*, *BCAM2531* genes were analyzed and *sigA* gene was used as an internal control (Table 1). The amount of mRNA detected normalized to control *sigA* mRNA values. Relative quantification of expression was calculated by ΔΔCT^69^.

**Table 1:** List of RT-PCR primers used in this study.

Gene	Primer	Sequence
*sigA*	Forward	5′-GCCGATGCGTTTCGGTAT-3′
	Reverse	5′-GCGTGACGTCGAACTGCTT-3′
*BCAM2418*	Forward	5′-CGCCAATACCTTCGTTCCA-3′
	Reverse	5′-CGGGATAGGCATTGGTGTTG-3′
*BCAM0729*	Forward	5′-GATCTCGGCTACGTCGAGTTTT-3′
	Reverse	5′-GTATTCACGACGAATTGCGTG-3′
*BCAL1829*	Forward	5′-CGTGTCGATCAACAAGAGCA-3′
	Reverse	5′-GACGTGCAGTTGACGAAGAA-3′
*BCAL2933*	Forward	5′-TCAAGTGGATGTTCGAAAAGCA-3′
	Reverse	5′-AATGGATGTATCAGATGCTGCG-3′
*BCAL3098*	Forward	5′-GCGGTGAAGGAGCAGTTG-3′
	Reverse	5′-CGGATTTCGACGAAGGACTG-3′
*BCAM1570*	Forward	5′-TATTCGGTGAACGGCAGCTA-3′
	Reverse	5′-TCACGGTCTACAAGGGCATT-3′
*BCAM2531*	Forward	5′-CGACGTGAAGATCGTCGAAT-3′
	Reverse	5′-CGAACGATCCGGTCAATGG-3′

### RNA sequencing

RNA sequencing was conducted as a service provided by Admera Health Biopharma Services (South Plainfield, NJ, USA), using a validated Transcriptomic Analysis Pipeline. The RNA quality was assessed and samples that had an RNA integrity number (RIN) value > 6.5 were used for further analysis. Three replicates of planktonic bacteria (control) and GPMV-adherent bacteria (sample) RNA samples were used to perform mRNA paired-end library construction with a TruSeq Stranded RNA with rRNA Depletion (Illumina, San Diego, CA, USA). Control bacteria were obtained from a 6 h fresh grown culture; after reaching mid-exponential phase, 1.5 × 10^9^ CFU of *B. cenocepacia* K56-2 were incubated in GPMV buffer at 37 °C in 5% CO_2_ for 30 min to mimic GPMV-adhesion.

Before alignment of sequence reads, quality check (FastQC), removal of adapter content (used during sequencing) and quality thresholding [remove any bad quality reads (Phred Score < 30)], were performed. The RNA-Seq reads were mapped against the genome and annotations of *B. cenocepacia* J2315 (obtained from Ensembl) to identify transcripts. Quantification of differential expressed transcripts was evaluated to estimate the relative abundances between groups (sample vs control). Normalization of the expression values [Log_2__FPKM (Fragments per Kilobase per Million)] was performed and the significantly up-regulated and down-regulated genes were identified. The significance threshold was *P*-value < 0.01 (FDR-adjusted *P*-value) and fold-change ≥ 1.5.

### Bioinformatic analysis

Heat maps of *B. cenocepacia* genes expression during adhesion to 16HBE14o- GPMVs were created using Heatmapper online platform (http://www.heatmapper.ca)^70^. To evaluate putative functions of the differentially expressed transcripts the identified genes were analyzed using Clusters of Orthologous Groups (COG) database. Gene annotation or predicted protein function were retrieved from the *B. cenocepacia* J2315 genome at Burkholderia Genome Database (http://www.burkholderia.com)^71^. Vononoi tessellations were created using Voronto mapper web service^27^. Genes were associated in Gene Ontology of KEGG pathway database^25,26^ obtained in ShinyGO v0.61 software^28^. Enrichment analysis were based on hypergeometric distribution followed by FDR correction^28^.

### Statistical analysis

Data are expressed as mean values of a minimum of three independent experiments ± standard error (SE). Statistical analysis was carried out by using GraphPad Prism 8.0.1 software. Relative comparisons were done among corrected values with ANOVA test for significance. Fisher exact test was used to identify significantly expressed COG. A *P*-value of < 0.05 was considered statistically significant in all analysis.

## Supplementary Information

Supplementary Information

## Data Availability

The sequencing data discussed in this publication have been deposited in NCBI's Gene Expression Omnibus^72^ and are accessible through GEO Series accession number GSE155982 (https://www.ncbi.nlm.nih.gov/geo/query/acc.cgi?acc=GSE155982). All processed data are available in the paper or supplementary information.
