# Supplementary material for: Burkholderia cenocepacia transcriptome during the early contacts with giant plasma membrane vesicles derived from live bronchial epithelial cells
Source: Sci Rep. 2021 Mar 11;11:5624. doi: 10.1038/s41598-021-85222-5 (PMC7970998; doi:10.1038/s41598-021-85222-5)
Supplement: Supplementary file 1 — Supplementary Information [file 41598_2021_85222_MOESM1_ESM.docx]

**Supplementary Materials:**

***Burkholderia cenocepacia* transcriptome during the early contacts with giant plasma membrane vesicles derived from live bronchial epithelial cells.**

Andreia I. Pimenta, Nuno Bernardes, Marta M. Alves, Dalila Mil-Homens, Arsenio M. Fialho


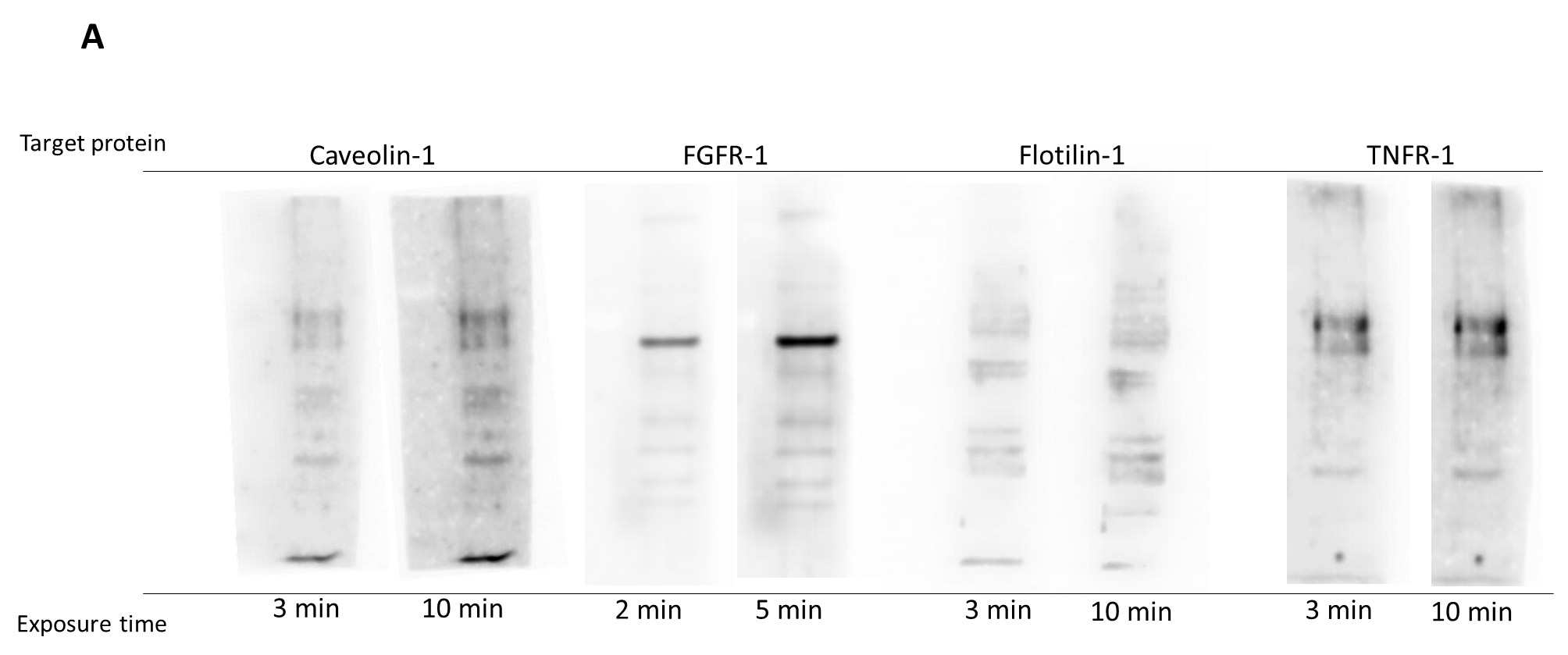


**
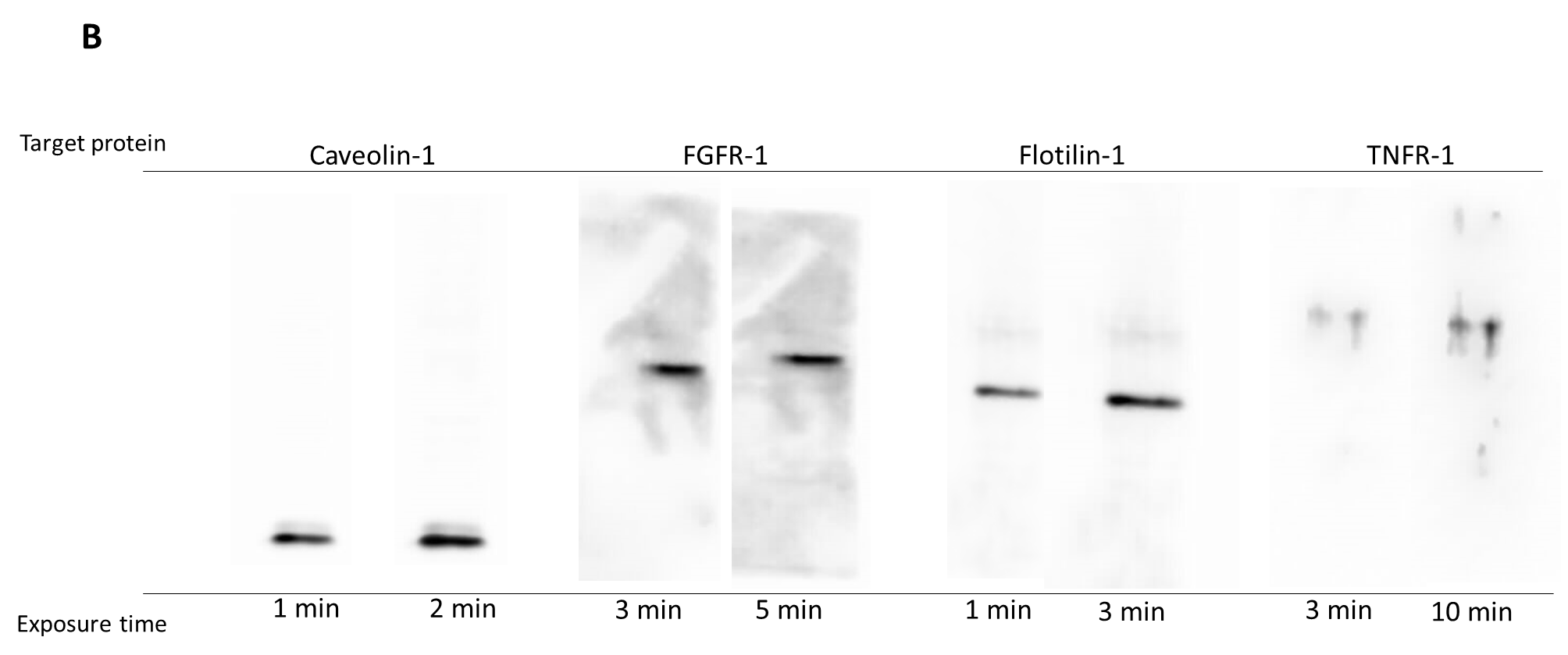
Supplementary Figure 1**. Western Blots of 16HBE14o- cellular (**A**) and vesicular (**B**) protein extracts. The presence of Caveolin-1, Flotillin-1, FGFR-1 and TNFR-1 was analyzed using specific antibodies. The exposure times was optimized for each target, ranging from 1min to 10min. Two different exposure times were acquired for each analysis. The chemiluminescence acquisition of the target proteins were performed using Fusion Solo (Viber Lourmat) equipment.

**Table S1**. List of genes up-regulated upon *B. cenocepacia* K56-2 adhesion (37ºC, 30min) to 16HBE14o- derived GPMVs. Genes were associated in Gene Ontology of KEGG pathway database obtained in ShinyGO v0.61 software. Enrichment analysis based on hypergeometric distribution followed by FDR correction^25,26,28^.

| **GENE** | **ANNOTATION OR PREDICTED FUNTION** | | | **FOLD CHANGE** | |
| --- | --- | --- | --- | --- | --- |
| **ABC TRANSPORTERS** | | | | | |
| **P-value 1.644e-64** | **Count 81/264** | | | **30.7%** | |
| *BCAL0015* | Putative branched-chain amino acid ABC transporter ATP-binding protein | | | 9.245 | |
| *BCAL0016* | Putative branched-chain amino acid ABC transporter ATP-binding protein | | | 9.245 | |
| *BCAL0017* | Putative branched-chain amino acid ABC transporter periplasmic protein | | | 26.628 | |
| *BCAL0018* | Putative branched-chain amino acid ABC transporter permease | | | 23.277 | |
| *BCAL0019* | Putative branched-chain amino acid ABC transporter permease | | | 23.277 | |
| *BCAL0151* | Extracellular ligand binding protein | | | 27.890 | |
| *BCAL0334* | Periplasmic solute-binding protein | | | 5.586 | |
| *BCAL0544* | Putative periplasmic dipeptide transport protein | | | 2.910 | |
| *BCAL0598* | Putrescine ABC transporter binding exported protein | | | 11.197 | |
| *BCAL0645* | Sulfate-binding protein | | | 8.995 | |
| *BCAL0675* | Extracellular solute-binding protein | | | 1.909 | |
| *BCAL1045* | Periplasmic ligand binding lipoprotein | | | 6.538 | |
| *BCAL1270* | *pstS* - Phosphate transport system substrate-binding exported periplasmic protein | | | 2.983 | |
| *BCAL1431* | Putative ribose ABC transporter substrate-binding exported protein | | | 34.592 | |
| *BCAL1432* | Putative sugar ABC transporter ATP-binding protein | | | 17.196 | |
| *BCAL1433* | Putative sugar transport system permease | | | 43.608 | |
| *BCAL1620* | *ssuC* - Aliphatic sulfonates transport permease | | | 116.169 | |
| *BCAL1621* | *ssuB* - Aliphatic sulfonates ABC transporter ATP-binding protein | | | 116.169 | |
| *BCAL1652* | Sulfate-binding protein | | | 71.467 | |
| *BCAL1653* | *cysT* - Sulfate transport system permease | | | 19.512 | |
| *BCAL1654* | *cysW* - Sulfate transport system permease | | | 19.512 | |
| *BCAL1655* | *cysA* - Sulfate ABC transporter ATP-binding protein | | | 8.557 | |
| *BCAL1657* | Putative ribose transport system | | | 2.021 | |
| *BCAL1691* | *orbC* - Putative iron transport-related ATP-binding protein | | | 8.639 | |
| *BCAL1692* | *orbD* - Iron-hydroxamate transporter permease subunit | | | 8.639 | |
| *BCAL2705* | ABC transporter ATP-binding protein | | | 4.189 | |
| *BCAL2706* | ABC transporter ATP-binding protein | | | 4.189 | |
| *BCAL2707* | Putative transport system permease | | | 5.043 | |
| *BCAL2708* | Putative amino-acid transport permease | | | 13.070 | |
| *BCAL2806* | Putative ABC transporter permease | | | 17.159 | |
| *BCAL2807* | Putative transmembrane ABC transporter protein | | | 17.159 | |
| *BCAL3038* | ABC transporter ATP-binding protein | | | 6.378 | |
| *BCAL3039* | ABC transporter permease | | | 13.731 | |
| *BCAL3040* | ABC transporter permease | | | 13.731 | |
| *BCAL3041* | *malE* - Maltose-binding protein | | | 10.453 | |
| *BCAL3098* | Putative branched-chain amino acid transporter substrate-binding protein | | | 293.494 | |
| *BCAL3099* | Putative branched-chain amino acid transporter permease | | | 21.606 | |
| *BCAL3102* | ABC transporter ATP-binding protein | | | 46.045 | |
| *BCAL3356* | *gltK* - Glutamate/aspartate transport system permease | | | 2.691 | |
| *BCAL3357* | *gltJ* - Glutamate/aspartate transport system permease | | | 2.691 | |
| *BCAL3358* | Periplasmic glutamate/aspartate-binding protein | | | 2.998 | |
| *BCAM0508* | Putative periplasmic binding protein | | | 8.604 | |
| *BCAM0610* | Putative ligand binding protein | | | 49.703 | |
| *BCAM0611* | Putative branched-chain amino acid transporter | | | 53.972 | |
| *BCAM0613* | ABC transporter ATP-binding protein | | | 12.402 | |
| *BCAM0760* | Histidine transport system permease | | | 7.509 | |
| *BCAM0761* | Histidine transport system permease | | | 7.509 | |
| *BCAM0766* | D-ribose-binding periplasmic protein precursor | | | 3.226 | |
| *BCAM0874* | Periplasmic solute-binding protein | | | 28.680 | |
| *BCAM1118* | Putative periplasmic solute-binding protein | | | 41.252 | |
| *BCAM1126* | Nitrate ABC transporter substrate-binding protein | | | 134.335 | |
| *BCAM1377* | ABC transporter ATP-binding protein | | | 76.572 | |
| *BCAM1378* | ABC transporter permease | | | 76.572 | |
| *BCAM1379* | ABC transporter substrate-binding protein | | | 38.129 | |
| *BCAM1743* | Periplasmic solute-binding protein | | | 8.064 | |
| *BCAM2251* | Putative amino acid ABC transporter solute binding protein | | | 2.702 | |
| *BCAM2312* | Putative glycine betaine transporter substrate binding protein | | | 6.268 | |
| *BCAM2317* | Glycine/betaine ABC transporter substrate-binding protein | | | 11.661 | |
| *BCAM2381* | Putative ABC transport exported protein | | | 2.198 | |
| *BCAM2382* | Putative ABC transporter system permease | | | 2.198 | |
| *BCAM2383* | ABC transporter ATP-binding protein | | | 2.198 | |
| *BCAM2384* | Putative ABC transporter system permease | | | 2.198 | |
| *BCAM2407* | Putative glycine betaine/L-proline ABC transporter substrate-binding protein | | | 11.112 | |
| *BCAM2409* | Putative glycine-betaine ABC transporter permease | | | 20.419 | |
| *BCAM2410* | Putative glycine betaine/L-proline ABC transporter ATP-binding protein | | | 20.419 | |
| *BCAM2528* | Putative ABC transporter permease | | | 63.054 | |
| *BCAM2529* | ABC transporter ATP-binding protein (complex MetNIQ - methionine import) | | | 63.054 | |
| *BCAM2531* | Putative ABC transporter solute-binding protein | | | 223.872 | |
| *BCAM2618* | Putative periplasmic lysine-arginine-ornithine-binding protein | | | 11.084 | |
| *BCAM2620* | Putative lipoprotein | | | 26.653 | |
| *BCAM2728* | *oppA* - Putative periplasmic oligopeptide-binding protein precursor | | | 22.729 | |
| *BCAS0110* | Periplasmic solute-binding protein | | | 14.969 | |
| *BCAS0111* | Putative binding-protein-dependent transport system protein | | | 55.369 | |
| *BCAS0112* | Putative binding-protein-dependent transport system protein | | | 22.214 | |
| *BCAS0113* | ABC transporter ATP-binding protein | | | 26.650 | |
| *BCAS0140* | Putative branched-chain amino acid ABC transporter substrate-binding protein | | | 8.003 | |
| *BCAS0141* | Putative transport system protein | | | 5.443 | |
| *BCAS0142* | Putative amino-acid transport system protein | | | 5.443 | |
| *BCAS0143* | ABC transporter ATP-binding protein | | | 5.443 | |
| *BCAS0144* | ABC transporter ATP-binding protein | | | 5.443 | |
| *BCAS0240* | Periplasmic solute-binding protein | | | 4.315 | |
| **METABOLIC PATHWAYS** | | | | | |
| **P-value 8.780e-16** | **Count 70/922** | | | **7.6%** | |
| *BCAL0010* | *phhA* - Phenylalanine 4-monooxygenase | | | 20.439 | |
| *BCAL0039* | *pheC* - Periplasmic cyclohexadienyl dehydratase | | | 3.444 | |
| *BCAL0059* | eutB - Ethanolamine ammonia-lyase heavy chain | | | 5.045 | |
| *BCAL0060* | eutC - Ethanolamine ammonia-lyase small subunit | | | 5.045 | |
| *BCAL0290* | *glt2* - Glutamate synthase subunit beta | | | 7.476 | |
| *BCAL0328* | *petA* - Ubiquinol-cytochrome C reductase iron-sulfur subunit | | | 2.350 | |
| *BCAL0329* | *petB* - Cytochrome B; Component of the ubiquinol-cytochrome C reductase complex | | | 2.350 | |
| *BCAL0600* | Putative glutamine synthetase | | | 8.838 | |
| *BCAL0603* | *puuC* - Gamma-glutamyl-gamma-aminobutyraldehyde dehydrogenase | | | 3.457 | |
| *BCAL0714* | Threonine peptidase, family T3 | | | 5.003 | |
| *BCAL0795* | *coaD* - Phosphopantetheine adenylyltransferase | | | 2.068 | |
| *BCAL0885* | Putative 3-hydroxyacyl-CoA dehydrogenase oxidoreductase | | | 2.019 | |
| *BCAL0886* | Acetyl-CoA acetyltransferase | | | 2.365 | |
| *BCAL0925* | *glpK* - Glycerol kinase | | | 10.076 | |
| *BCAL1183* | Aldehyde dehydrogenase family protein | | | 2.969 | |
| *BCAL1427* | Myo-inositol catabolism protein | | | 11.695 | |
| *BCAL1428* | Putative amine catabolism-related protein | | | 13.589 | |
| *BCAL1429* | Putative TPP-binding acetolactate synthase | | | 30.290 | |
| *BCAL1435* | *idh* - Inositol 2-dehydrogenase | | | 15.127 | |
| *BCAL1541* | Putative acyl-CoA synthetase | | | 7.385 | |
| *BCAL1862* | *phbA* - Acetyl-CoA acetyltransferase | | | 1.852 | |
| *BCAL1979* | Putative fatty acid degradation protein | | | 2.770 | |
| *BCAL2118* | *aceA* - Isocitrate lyase | | | 4.940 | |
| *BCAL2121* | Putative dehalogenase | | | 35.004 | |
| *BCAL2122* | *aceB* - Malate synthase | | | 7.437 | |
| *BCAL2198* | Cysteine desulfurase | | | 12.614 | |
| *BCAL2224* | *glnA* - Glutamine synthetase | | | 29.409 | |
| *BCAL2284* | *acoE* - Acetyl-CoA synthetase | | | 2.617 | |
| *BCAL2304* | 3-hydroxybutyrate dehydrogenase | | | 4.560 | |
| *BCAL2357* | *ilvC* - Ketol-acid reductoisomerase | | | 1.814 | |
| *BCAL2358* | *ilvH* - Acetolactate synthase 3 regulatory subunit | | | 2.692 | |
| *BCAL2683* | *cysH* - Phosphoadenosine phosphosulfate reductase | | | 6.753 | |
| *BCAL2685* | *cysI* - Putative sulfite reductase | | | 17.298 | |
| *BCAL2735* | Isocitrate dehydrogenase | | | 1.996 | |
| *BCAL3043* | *pgl* - 6-phosphogluconolactonase | | | 3.027 | |
| *BCAL3044* | *glk* - Bifunctional glucokinase/RpiR family transcriptional regulator | | | 3.027 | |
| *BCAL3104* | *ureA* - Urease subunit gamma | | | 35.232 | |
| *BCAL3106* | *ureC* - Urease subunit alpha | | | 40.304 | |
| *BCAL3191* | Putative glutaryl-CoA dehydrogenase | | | 3.150 | |
| *BCAL3282* | Phospho-2-dehydro-3-deoxyheptonate aldolase | | | 3.730 | |
| *BCAL3325* | *pntB* - NAD(P) transhydrogenase subunit beta | | | 4.404 | |
| *BCAL3326* | *pntAB* - NAD(P) transhydrogenase subunit alpha | | | 4.404 | |
| *BCAL3366* | *eda* - KHG/KDPG aldolase | | | 3.340 | |
| *BCAL3474* | *fadD* - Long-chain-fatty-acid--CoA ligase | | | 3.132 | |
| *BCAM0018* | N-acetyl-gamma-glutamyl-phosphate reductase | | | 60.237 | |
| *BCAM0187* | 2-isopropylmalate synthase | | | 8.786 | |
| *BCAM0540* | Putative serine acetyltransferase | | | 79.962 | |
| *BCAM0543* | Putative aminotransferase | | | 48.664 | |
| *BCAM0544* | Putative acetylglutamate kinase | | | 14.310 | |
| *BCAM0721* | O-acetylhomoserine (thiol)-lyase | | | 10.513 | |
| *BCAM0949* | *lip* - exported lipase LipA | | | 7.761 | |
| *BCAM0983* | *leuC1* - Isopropylmalate isomerase large subunit | | | 2.395 | |
| *BCAM0984* | *leuD1* - Isopropylmalate isomerase small subunit | | | 3.423 | |
| *BCAM1309* | *ggt2* - Gamma-glutamyltransferase precursor 2 | | | 6.604 | |
| *BCAM1488* | Putative proline racemase | | | 17.521 | |
| *BCAM1588* | Isocitrate lyase | | | 5.194 | |
| *BCAM1822* | Putative NAD-dependent glutamate dehydrogenase | | | 2.501 | |
| *BCAM1833* | *acnB* - Bifunctional aconitate hydratase 2/2-methylisocitrate dehydratase | | | 2.952 | |
| *BCAM1955* | *ggt1* - Gamma-glutamyltransferase precursor 1 | | | 3.460 | |
| *BCAM2094* | *puuA* - putative gamma-glutamylputrescine synthetase | | | 12.352 | |
| *BCAM2326* | Serine hydroxymethyltransferase | | | 23.996 | |
| *BCAM2368* | Putative quinoprotein ethanol dehydrogenase precursor | | | 259.175 | |
| *BCAM2372* | *acsA* - Acetyl-CoA synthetase | | | 19.709 | |
| *BCAM2501* | Shikimate 5-dehydrogenase | | | 11.496 | |
| *BCAM2502* | *aroQ* - 3-dehydroquinate dehydratase | | | 11.496 | |
| *BCAM2561* | Putative 4-aminobutyrate aminotransferase | | | 2.388 | |
| *BCAS0731* | *dhT* - Phenylhydantoinase | | | 24.674 | |
| *BCAS0733* | Dihydropyrimidine dehydrogenase | | | 29.277 | |
| *BCAS0734* | Putative oxidoreductase | | | 28.410 | |
| *BCAS0751* | Putative gamma-glutamyltransferase precursor | | | 7.275 | |
| **SULFUR METABOLISM** | | | | | |
| **P-value 4.363e-12** | **Count 16/56** | | | **28.6%** | |
| *BCAL0645* | Sulfate-binding protein | | | 8.995 | |
| *BCAL1619* | *ssuD* - Alkanesulfonate monooxygenase | | | 188.767 | |
| *BCAL1620* | *ssuC* - Aliphatic sulfonates transport permease | | | 116.169 | |
| *BCAL1621* | *ssuB* - Aliphatic sulfonates ABC transporter ATP-binding protein | | | 116.169 | |
| *BCAL1652* | Sulfate-binding protein | | | 71.467 | |
| *BCAL1653* | *cysT* - Sulfate transport system permease | | | 19.512 | |
| *BCAL1654* | *cysW* - Sulfate transport system permease | | | 19.512 | |
| *BCAL1655* | *cysA* - Sulfate ABC transporter ATP-binding protein | | | 8.557 | |
| *BCAL2683* | *cysH* - Phosphoadenosine phosphosulfate reductase | | | 6.753 | |
| *BCAL2685* | *cysI* - Putative sulfite reductase | | | 17.298 | |
| *BCAM0540* | Putative serine acetyltransferase | | | 79.962 | |
| *BCAM1118* | Putative periplasmic solute-binding protein | | | 41.252 | |
| *BCAM1121* | Putative taurine dioxygenase | | | 20.202 | |
| *BCAM1122* | Putative taurine dioxygenase | | | 21.175 | |
| *BCAM1126* | Nitrate ABC transporter substrate-binding protein | | | 134.335 | |
| *BCAS0426* | Putative taurine dioxygenase | | | 252.922 | |
| **MICROBIAL METABOLISM IN DIVERSE ENVIRONMENTS** | | | | | |
| **P-value 9.188e-12** | **Count 38/393** | | | **9.7%** | |
| *BCAL0064* | acoD - Acetaldehyde dehydrogenase | | | 16.817 | |
| *BCAL0289* | glt1 - Glutamate synthase large subunit | | | 8.139 | |
| *BCAL0290* | *glt2* - Glutamate synthase subunit beta | | | 7.476 | |
| *BCAL0600* | Putative glutamine synthetase | | | 8.838 | |
| *BCAL0885* | Putative 3-hydroxyacyl-CoA dehydrogenase oxidoreductase | | | 2.019 | |
| *BCAL0886* | Acetyl-CoA acetyltransferase | | | 2.365 | |
| *BCAL1183* | Aldehyde dehydrogenase family protein | | | 2.969 | |
| *BCAL1427* | Myo-inositol catabolism protein | | | 11.695 | |
| *BCAL1428* | Putative amine catabolism-related protein | | | 13.589 | |
| *BCAL1429* | Putative TPP-binding acetolactate synthase | | | 30.290 | |
| *BCAL1435* | *idh* - Inositol 2-dehydrogenase | | | 15.127 | |
| *BCAL1861* | *phaC* - Acetyacetyl-CoA reductase | | | 2.482 | |
| *BCAL1862* | *phbA* - Acetyl-CoA acetyltransferase | | | 1.852 | |
| *BCAL1979* | Putative fatty acid degradation protein | | | 2.770 | |
| *BCAL2118* | *aceA* - Isocitrate lyase | | | 4.940 | |
| *BCAL2121* | Putative dehalogenase | | | 35.004 | |
| *BCAL2122* | *aceB* - Malate synthase | | | 7.437 | |
| *BCAL2224* | *glnA* - Glutamine synthetase | | | 29.409 | |
| *BCAL2284* | *acoE* - Acetyl-CoA synthetase | | | 2.617 | |
| *BCAL2683* | *cysH* - Phosphoadenosine phosphosulfate reductase | | | 6.753 | |
| *BCAL2685* | *cysI* - Putative sulfite reductase | | | 17.298 | |
| *BCAL2735* | Isocitrate dehydrogenase | | | 1.996 | |
| *BCAL3043* | *pgl* - 6-phosphogluconolactonase | | | 3.027 | |
| *BCAL3044* | *glk* - Bifunctional glucokinase/RpiR family transcriptional regulator | | | 3.027 | |
| *BCAL3104* | *ureA* - Urease subunit gamma | | | 35.232 | |
| *BCAL3106* | *ureC* - Urease subunit alpha | | | 40.304 | |
| *BCAL3191* | Putative glutaryl-CoA dehydrogenase | | | 3.150 | |
| *BCAL3366* | *eda* - KHG/KDPG aldolase | | | 3.340 | |
| *BCAM0131* | *hchA* - Chaperone protein HchA | | | 4.096 | |
| *BCAM0540* | Putative serine acetyltransferase | | | 79.962 | |
| *BCAM1588* | Isocitrate lyase | | | 5.194 | |
| *BCAM1685* | *nirB* - Putative nitrite reductase | | | 108.386 | |
| *BCAM1833* | *acnB* - Bifunctional aconitate hydratase 2/2-methylisocitrate dehydratase | | | 2.952 | |
| *BCAM2094* | *puuA* - putative gamma-glutamylputrescine synthetase | | | 12.352 | |
| *BCAM2326* | serine hydroxymethyltransferase | | | 23.996 | |
| *BCAM2368* | Putative quinoprotein ethanol dehydrogenase precursor | | | 259.175 | |
| *BCAM2372* | *acsA* - Acetyl-CoA synthetase | | | 19.709 | |
| *BCAS0734* | Putative oxidoreductase | | | 28.410 | |
| **BIOSYNTHESIS OF AMINO ACIDS** | | | | | |
| **P-value 1.227e-09** | **Count 21/149** | | | **14.1%** | |
| *BCAL0010* | *phhA* - Phenylalanine 4-monooxygenase | | | 20.439 | |
| *BCAL0039* | *pheC* - Periplasmic cyclohexadienyl dehydratase | | | 3.444 | |
| *BCAL0290* | *glt2* - Glutamate synthase subunit beta | | | 7.476 | |
| *BCAL0600* | Putative glutamine synthetase | | | 8.838 | |
| *BCAL2224* | *glnA* - Glutamine synthetase | | | 29.409 | |
| *BCAL2357* | *ilvC* - Ketol-acid reductoisomerase | | | 1.814 | |
| *BCAL2358* | *ilvH* - Acetolactate synthase 3 regulatory subunit | | | 2.692 | |
| *BCAL2735* | Isocitrate dehydrogenase | | | 1.996 | |
| *BCAL3282* | Phospho-2-dehydro-3-deoxyheptonate aldolase | | | 3.730 | |
| *BCAM0018* | N-acetyl-gamma-glutamyl-phosphate reductase | | | 60.237 | |
| *BCAM0187* | 2-isopropylmalate synthase | | | 8.786 | |
| *BCAM0540* | Putative serine acetyltransferase | | | 79.962 | |
| *BCAM0544* | Putative acetylglutamate kinase | | | 14.310 | |
| *BCAM0983* | *leuC1* - Isopropylmalate isomerase large subunit | | | 2.395 | |
| *BCAM0984* | *leuD1* - Isopropylmalate isomerase small subunit | | | 3.423 | |
| *BCAM1833* | *acnB* - Bifunctional aconitate hydratase 2/2-methylisocitrate dehydratase | | | 2.952 | |
| *BCAM2094* | *puuA* - putative gamma-glutamylputrescine synthetase | | | 12.352 | |
| *BCAM2326* | serine hydroxymethyltransferase | | | 23.996 | |
| *BCAM2501* | Shikimate 5-dehydrogenase | | | 11.496 | |
| *BCAM2502* | *aroQ* - 3-dehydroquinate dehydratase | | | 11.496 | |
| *BCAS0734* | Putative oxidoreductase | | | 28.410 | |
| **TWO-COMPONENT SYSTEM** | | | | | |
| **P-value 5.501e-09** | **Count 19/132** | | | **14.4%** | |
| *BCAL0328* | *petA* - Ubiquinol-cytochrome C reductase iron-sulfur subunit | | | 2.350 | |
| *BCAL0329* | *petB* - Cytochrome B; Component of the ubiquinol-cytochrome C reductase complex | | | 2.350 | |
| *BCAL0600* | Putative glutamine synthetase | | | 8.838 | |
| *BCAL1270* | *pstS* - Phosphate transport system substrate-binding exported periplasmic protein | | | 2.983 | |
| *BCAL1862* | *phbA* - Acetyl-CoA acetyltransferase | | | 1.852 | |
| *BCAL2222* | *glnG* - nitrogen regulation protein NR(I) | | | 13.697 | |
| *BCAL2223* | *glnL* - Putative nitrogen regulation protein NR(II) | | | 20.857 | |
| *BCAL2224* | *glnA* - Glutamine synthetase | | | 29.409 | |
| *BCAL2379* | *kdpE* - two-component regulatory system, response regulator protein | | | 6.253 | |
| *BCAL2380* | *kdpD* - two-component regulatory system, sensor kinase protein | | | 6.253 | |
| *BCAL2381* | *kdpC* - Potassium-transporting ATPase subunit C | | | 20.226 | |
| *BCAL2382* | *kdpB* - Potassium-transporting ATPase subunit B | | | 9.614 | |
| *BCAL2383* | *kdpA* - potassium-transporting ATPase subunit A | | | 5.524 | |
| *BCAL3356* | *gltK* - Glutamate/aspartate transport system permease | | | 2.691 | |
| *BCAL3357* | *gltJ* - Glutamate/aspartate transport system permease | | | 2.691 | |
| *BCAL3358* | Periplasmic glutamate/aspartate-binding protein | | | 2.998 | |
| *BCAM1743* | Periplasmic solute-binding protein | | | 8.064 | |
| *BCAM2094* | *puuA* - putative gamma-glutamylputrescine synthetase | | | 12.352 | |
| *BCAS0240* | Periplasmic solute-binding protein | | | 4.315 | |
| **TAURINE AND HYPOTAURINE METABOLISM** | | | | | |
| **P-value 5.508e-09** | **Count 8/15** | | | **53.3%** | |
| *BCAL0714* | Threonine peptidase, family T3 | | | 5.003 | |
| *BCAM1121* | Putative taurine dioxygenase | | | 20.202 | |
| *BCAM1122* | Putative taurine dioxygenase | | | 21.175 | |
| *BCAM1309* | *ggt2* - gamma-glutamyltransferase precursor 2 | | | 6.604 | |
| *BCAM1822* | Putative NAD-dependent glutamate dehydrogenase | | | 2.501 | |
| *BCAM1955* | *ggt1* - gamma-glutamyltransferase precursor 1 | | | 3.460 | |
| *BCAS0426* | Putative taurine dioxygenase | | | 252.922 | |
| *BCAS0751* | Putative gamma-glutamyltransferase precursor | | | 7.275 | |
| **NITROGEN METABOLISM** | | | | | |
| **P-value 1.986e-08** | **Count 9/24** | | | **37.5%** | |
| *BCAL0289* | glt1 - Glutamate synthase large subunit | | | 8.139 | |
| *BCAL0290* | *glt2* - Glutamate synthase subunit beta | | | 7.476 | |
| *BCAL0600* | Putative glutamine synthetase | | | 8.838 | |
| *BCAL2224* | *glnA* - Glutamine synthetase | | | 29.409 | |
| *BCAM1685* | *nirB* - Putative nitrite reductase | | | 108.386 | |
| *BCAM1686* | Putative nitrate transporter | | | 103.320 | |
| *BCAM1822* | Putative NAD-dependent glutamate dehydrogenase | | | 2.501 | |
| *BCAM2094* | *puuA* - putative gamma-glutamylputrescine synthetase | | | 12.352 | |
| *BCAS0734* | Putative oxidoreductase | | | 28.410 | |
| **GLYOXYLATE AND DICARBOXYLATE METABOLISM** | | | | | |
| **P-value 5.478e-07** | **Count 12/68** | | | **17.7%** | |
| *BCAL0289* | *glt1* - Glutamate synthase large subunit | | | 8.139 | |
| *BCAL0600* | Putative glutamine synthetase | | | 8.838 | |
| *BCAL1861* | *phaC* - Acetyacetyl-CoA reductase | | | 2.482 | |
| *BCAL1862* | *phbA* - Acetyl-CoA acetyltransferase | | | 1.852 | |
| *BCAL2118* | *aceA* - Isocitrate lyase | | | 4.940 | |
| *BCAL2122* | *aceB* - Malate synthase | | | 7.437 | |
| *BCAL2224* | *glnA* - Glutamine synthetase | | | 29.409 | |
| *BCAL3366* | *eda* - KHG/KDPG aldolase | | | 3.340 | |
| *BCAM1588* | Isocitrate lyase | | | 5.194 | |
| *BCAM1833* | *acnB* - Bifunctional aconitate hydratase 2/2-methylisocitrate dehydratase | | | 2.952 | |
| *BCAM2094* | *puuA* - putative gamma-glutamylputrescine synthetase | | | 12.352 | |
| *BCAM2326* | Serine hydroxymethyltransferase | | | 23.996 | |
| **ARGININE AND PROLINE METABOLISM** | | | | | |
| **P-value 9.514e-07** | **Count 12/72** | | | **16.7%** | |
| *BCAL0599* | Putative aminotransferase | | | 8.504 | |
| *BCAL0600* | Putative glutamine synthetase | | | 8.838 | |
| *BCAL0603* | *puuC* - Gamma-glutamyl-gamma-aminobutyraldehyde dehydrogenase | | | 3.457 | |
| *BCAL2224* | *glnA* - Glutamine synthetase | | | 29.409 | |
| *BCAL3104* | *ureA* - Urease subunit gamma | | | 35.232 | |
| *BCAL3106* | *ureC* - Urease subunit alpha | | | 40.304 | |
| *BCAM0018* | N-acetyl-gamma-glutamyl-phosphate reductase | | | 60.237 | |
| *BCAM0544* | Putative acetylglutamate kinase | | | 14.310 | |
| *BCAM1488* | Putative proline racemase | | | 17.521 | |
| *BCAM1822* | Putative NAD-dependent glutamate dehydrogenase | | | 2.501 | |
| *BCAM2094* | *puuA* - putative gamma-glutamylputrescine synthetase | | | 12.352 | |
| *BCAM2366* | Putative proline iminopeptidase | | | 25.964 | |
| **2-OXOCARBOXYLIC ACID METABOLISM** | | | | | |
| **P-value 1.685e-06** | **Count 9/39** | | | **23.1%** | |
| *BCAL2357* | *ilvC* - Ketol-acid reductoisomerase | | | 1.814 | |
| *BCAL2358* | *ilvH* - Acetolactate synthase 3 regulatory subunit | | | 2.692 | |
| *BCAL2735* | Isocitrate dehydrogenase | | | 1.996 | |
| *BCAM0018* | N-acetyl-gamma-glutamyl-phosphate reductase | | | 60.237 | |
| *BCAM0187* | 2-isopropylmalate synthase | | | 8.786 | |
| *BCAM0544* | Putative acetylglutamate kinase | | | 14.310 | |
| *BCAM0983* | *leuC1* - Isopropylmalate isomerase large subunit | | | 2.395 | |
| *BCAM0984* | *leuD1* - isopropylmalate isomerase small subunit | | | 3.423 | |
| *BCAM1833* | *acnB* - Bifunctional aconitate hydratase 2/2-methylisocitrate dehydratase | | | 2.952 | |
| **BUTANOATE METABOLISM** | | | | | |
| **P-value 5.674e-06** | **Count 10/58** | | | **17.2%** | |
| *BCAL1183* | Aldehyde dehydrogenase family protein | | | 2.969 | |
| *BCAL1472* | *scoA* - Succinyl-CoA:3-ketoacid-coenzyme A transferase subunit A | | | 6.643 | |
| *BCAL1473* | *scoB* - Succinyl-CoA:3-ketoacid-coenzyme A transferase subunit B | | | 7.024 | |
| *BCAL1861* | *phaC* - Acetyacetyl-CoA reductase | | | 2.482 | |
| *BCAL1862* | *phbA* - Acetyl-CoA acetyltransferase | | | 1.852 | |
| *BCAL1863* | *phbC* - Poly-beta-hydroxybutyrate polymerase | | | 3.192 | |
| *BCAL2304* | 3-hydroxybutyrate dehydrogenase | | | 4.560 | |
| *BCAL2358* | *ilvH* - Acetolactate synthase 3 regulatory subunit | | | 2.692 | |
| *BCAL2420* | Putative depolymerase/histone-like protein | | | 1.840 | |
| *BCAM2561* | Putative 4-aminobutyrate aminotransferase | | | 2.388 | |
| **CARBON METABOLISM** | | | | | |
| **P-value 5.674e-06** | **Count 15/136** | | | **11.0%** | |
| *BCAL0885* | Putative 3-hydroxyacyl-CoA dehydrogenase oxidoreductase | | | 2.019 | |
| *BCAL1862* | *phbA* - Acetyl-CoA acetyltransferase | | | 1.852 | |
| *BCAL1979* | Putative fatty acid degradation protein | | | 2.770 | |
| *BCAL2118* | *aceA* - Isocitrate lyase | | | 4.940 | |
| *BCAL2122* | *aceB* - Malate synthase | | | 7.437 | |
| *BCAL2284* | *acoE* - Acetyl-CoA synthetase | | | 2.617 | |
| *BCAL2735* | Isocitrate dehydrogenase | | | 1.996 | |
| *BCAL3043* | *pgl* - 6-phosphogluconolactonase | | | 3.027 | |
| *BCAL3044* | *glk* - Bifunctional glucokinase/RpiR family transcriptional regulator | | | 3.027 | |
| *BCAL3366* | *eda* - KHG/KDPG aldolase | | | 3.340 | |
| *BCAM0540* | Putative serine acetyltransferase | | | 79.962 | |
| *BCAM1588* | Isocitrate lyase | | | 5.194 | |
| *BCAM1833* | *acnB* - Bifunctional aconitate hydratase 2/2-methylisocitrate dehydratase | | | 2.952 | |
| *BCAM2326* | Serine hydroxymethyltransferase | | | 23.996 | |
| *BCAM2372* | *acsA* - Acetyl-CoA synthetase | | | 19.709 | |
| **FATTY ACID DEGRADATION** | | | | | |
| **P-value 6.530e-06** | **Count 8/35** | | | **22.9%** | |
| *BCAL0885* | Putative 3-hydroxyacyl-CoA dehydrogenase oxidoreductase | | | 2.019 | |
| *BCAL0886* | Acetyl-CoA acetyltransferase | | | 2.365 | |
| *BCAL1541* | Putative acyl-CoA synthetase | | | 7.385 | |
| *BCAL1979* | Putative fatty acid degradation protein | | | 2.770 | |
| *BCAL3029* | Putative alkane monooxygenase | | | 7.469 | |
| *BCAL3191* | Putative glutaryl-CoA dehydrogenase | | | 3.150 | |
| *BCAL3474* | *fadD* - Long-chain-fatty-acid-CoA ligase | | | 3.132 | |
| **ALANINE, ASPARTATE AND GLUTAMATE METABOLISM** | | | | | |
| **P-value 9.575e-06** | **Count 8/37** | | | **21.6%** | |
| *BCAL0290* | *glt2* - Glutamate synthase subunit beta | | | 7.476 | |
| *BCAL0600* | Putative glutamine synthetase | | | 8.838 | |
| *BCAL1183* | Aldehyde dehydrogenase family protein | | | 2.969 | |
| *BCAL2224* | *glnA* - Glutamine synthetase | | | 29.409 | |
| *BCAM1822* | Putative NAD-dependent glutamate dehydrogenase | | | 2.501 | |
| *BCAM2094* | *puuA* - putative gamma-glutamylputrescine synthetase | | | 12.352 | |
| *BCAM2561* | Putative 4-aminobutyrate aminotransferase | | | 2.388 | |
| *BCAS0734* | Putative oxidoreductase | | | 28.410 | |
| **BIOSYNTHESIS OF SECONDARY METABOLITES** | | | | | |
| **P-value 1.150e-05** | **Count 25/359** | | | **7.0%** | |
| *BCAL0039* | *pheC* - Periplasmic cyclohexadienyl dehydratase | | | 3.444 | |
| *BCAL0290* | *glt2* - Glutamate synthase subunit beta | | | 7.476 | |
| *BCAL0884* | Putative acyl-CoA dehydrogenase oxidoreductase protein | | | 1.704 | |
| *BCAL0886* | Acetyl-CoA acetyltransferase | | | 2.365 | |
| *BCAL1435* | *idh* - Inositol 2-dehydrogenase | | | 15.127 | |
| *BCAL1862* | *phbA* - Acetyl-CoA acetyltransferase | | | 1.852 | |
| *BCAL2284* | *acoE* - Acetyl-CoA synthetase | | | 2.617 | |
| *BCAL2357* | *ilvC* - Ketol-acid reductoisomerase | | | 1.814 | |
| *BCAL2358* | *ilvH* - Acetolactate synthase 3 regulatory subunit | | | 2.692 | |
| *BCAL2735* | Isocitrate dehydrogenase | | | 1.996 | |
| *BCAL3043* | *pgl* - 6-phosphogluconolactonase | | | 3.027 | |
| *BCAL3044* | *glk* - Bifunctional glucokinase/RpiR family transcriptional regulator | | | 3.027 | |
| *BCAL3282* | Phospho-2-dehydro-3-deoxyheptonate aldolase | | | 3.730 | |
| *BCAM0018* | N-acetyl-gamma-glutamyl-phosphate reductase | | | 60.237 | |
| *BCAM0187* | 2-isopropylmalate synthase | | | 8.786 | |
| *BCAM0544* | Putative acetylglutamate kinase | | | 14.310 | |
| *BCAM0983* | *leuC1* - Isopropylmalate isomerase large subunit | | | 2.395 | |
| *BCAM0984* | *leuD1* - Isopropylmalate isomerase small subunit | | | 3.423 | |
| *BCAM1833* | *acnB* - Bifunctional aconitate hydratase 2/2-methylisocitrate dehydratase | | | 2.952 | |
| *BCAM2326* | Serine hydroxymethyltransferase | | | 23.996 | |
| *BCAM2368* | Putative quinoprotein ethanol dehydrogenase precursor | | | 259.175 | |
| *BCAM2372* | *acsA* - Acetyl-CoA synthetase | | | 19.709 | |
| *BCAM2501* | Shikimate 5-dehydrogenase | | | 11.496 | |
| *BCAM2502* | *aroQ* - 3-dehydroquinate dehydratase | | | 11.496 | |
| *BCAS0734* | Putative oxidoreductase | | | 28.410 | |
| **ARACHIDONIC ACID METABOLISM** | | | | | |
| **P-value 1.688e-05** | **Count 4/6** | | | **66.7%** | |
| *BCAL0714* | Threonine peptidase, family T3 | | | 5.003 | |
| *BCAM1309* | *ggt2* - Gamma-glutamyltransferase precursor 2 | | | 6.604 | |
| *BCAM1955* | *ggt1* - Gamma-glutamyltransferase precursor 1 | | | 3.460 | |
| *BCAS0751* | Putative gamma-glutamyltransferase precursor | | | 7.275 | |
| **CYANOAMINO ACID METABOLISM** | | | | | |
| **P-value 1.852e-05** | **Count 5/12** | | | **41.7%** | |
| *BCAL0714* | Threonine peptidase, family T3 | | | 5.003 | |
| *BCAM1309* | *ggt2* - Gamma-glutamyltransferase precursor 2 | | | 6.604 | |
| *BCAM1955* | *ggt1* - Gamma-glutamyltransferase precursor 1 | | | 3.460 | |
| *BCAM2326* | Serine hydroxymethyltransferase | | | 23.996 | |
| *BCAS0751* | Putative gamma-glutamyltransferase precursor | | | 7.275 | |
| **FLAGELLAR ASSEMBLY** | | | | | |
| **P-value 0.00014** | **Count 7/40** | | | **17.5%** | |
| *BCAL0140* | *flhB* - Flagellar biosynthesis protein FlhB | | | 2.470 | |
| *BCAL0141* | *flhA* - Flagellar biosynthesis protein FlhA | | | 2.470 | |
| *BCAL0523* | *fliH* - Flagellar assembly protein H | | | 2.099 | |
| *BCAL0524* | *fliG* - Flagellar motor switch protein G | | | 2.099 | |
| *BCAL0525* | *fliF* - Flagellar MS-ring protein | | | 2.099 | |
| *BCAL0567* | *flgE1* - Flagellar hook protein FlgE | | | 2.083 | |
| *BCAL3506* | *fliM* - Flagellar motor switch protein FliM | | | 3.611 | |
| **VALINE, LEUCINE, AND ISOLEUCINE BIOSYNTHESIS** | | | | | |
| **P-value 0.00086** | **Count 5/25** | | | **20.0%** | |
| *BCAL2357* | *ilvC* - Ketol-acid reductoisomerase | | | 1.814 | |
| *BCAL2358* | *ilvH* - Acetolactate synthase 3 regulatory subunit | | | 2.692 | |
| *BCAM0187* | 2-isopropylmalate synthase | | | 8.786 | |
| *BCAM0983* | *leuC1* - Isopropylmalate isomerase large subunit | | | 2.395 | |
| *BCAM0984* | *leuD1* - Isopropylmalate isomerase small subunit | | | 3.423 | |
| **SYNTHESIS AND DEGRADATION OF KETONE BODIES** | | | | | |
| **P-value 0.00097** | **Count 4/15** | | | **26.7%** | |
| *BCAL1472* | *scoA* - Succinyl-CoA:3-ketoacid-coenzyme A transferase subunit A | | | 6.643 | |
| *BCAL1473* | *scoB* - Succinyl-CoA:3-ketoacid-coenzyme A transferase subunit B | | | 7.024 | |
| *BCAL1862* | *phbA* - Acetyl-CoA acetyltransferase | | | 1.852 | |
| *BCAL2304* | 3-hydroxybutyrate dehydrogenase | | | 4.560 | |
| **GLUTATHIONE METABOLISM** | | | | | |
| **P-value 0.00097** | **Count 6/40** | | | **15.0%** | |
| *BCAL0714* | Threonine peptidase, family T3 | | | 5.003 | |
| *BCAL2321* | Putative glutathione S-transferase | | | 2.074 | |
| *BCAL2735* | Isocitrate dehydrogenase | | | 1.996 | |
| *BCAM1309* | *ggt2* - Gamma-glutamyltransferase precursor 2 | | | 6.604 | |
| *BCAM1955* | *ggt1* - Gamma-glutamyltransferase precursor 1 | | | 3.460 | |
| *BCAS0751* | Putative gamma-glutamyltransferase precursor | | | 7.275 | |
| **BACTERIAL CHEMOTAXIS** | | | | | |
| **P-value 0.00097** | **Count 6/40** | | | **15.0%** | |
| *BCAL0524* | *fliG* - Flagellar motor switch protein G | | | 2.099 | |
| *BCAL0544* | Putative periplasmic dipeptide transport protein | | | 2.910 | |
| *BCAL0675* | Extracellular solute-binding protein | | | 1.909 | |
| *BCAL1657* | Putative ribose transport system | | | 2.021 | |
| *BCAL3506* | *fliM* - Flagellar motor switch protein FliM | | | 3.611 | |
| *BCAM0766* | D-ribose-binding periplasmic protein precursor | | | 3.226 | |
| **PHENYLALANINE, TYROSINE AND TRYPTOPHAN BIOSYNTHESIS** | | | | | |
| **P-value 0.00104** | **Count 5/27** | | | **18.5%** | |
| *BCAL0010* | *phhA* - Phenylalanine 4-monooxygenase | | | 20.439 | |
| *BCAL0039* | *pheC* - Periplasmic cyclohexadienyl dehydratase | | | 3.444 | |
| *BCAL3282* | Phospho-2-dehydro-3-deoxyheptonate aldolase | | | 3.730 | |
| *BCAM2501* | Shikimate 5-dehydrogenase | | | 11.496 | |
| *BCAM2502* | *aroQ* - 3-dehydroquinate dehydratase | | | 11.496 | |
| **PANTOTHENATE AND CoA BIOSYNTHESIS** | | | | | |
| **P-value 0.00166** | **Count 5/30** | | | **16.7%** | |
| *BCAL0795* | *coaD* - Phosphopantetheine adenylyltransferase | | | 2.068 | |
| *BCAL2357* | *ilvC* - Ketol-acid reductoisomerase | | | 1.814 | |
| *BCAL2358* | *ilvH* - Acetolactate synthase 3 regulatory subunit | | | 2.692 | |
| *BCAS0731* | *dhT* - Phenylhydantoinase | | | 24.674 | |
| *BCAS0733* | Dihydropyrimidine dehydrogenase | | | 29.277 | |
| **PROPANOATE METABOLISM** | | | | | |
| **P-value 0.00259** | **Count 6/49** | | | **12.2%** | |
| *BCAL1862* | *phbA* - Acetyl-CoA acetyltransferase | | | 1.852 | |
| *BCAL2284* | *acoE* - Acetyl-CoA synthetase | | | 2.617 | |
| *BCAM1833* | *acnB* - Bifunctional aconitate hydratase 2/2-methylisocitrate dehydratase | | | 2.952 | |
| *BCAM2368* | Putative quinoprotein ethanol dehydrogenase precursor | | | 259.175 | |
| *BCAM2372* | *acsA* - Acetyl-CoA synthetase | | | 19.709 | |
| *BCAM2561* | Putative 4-aminobutyrate aminotransferase | | | 2.388 | |
| **PYRUVATE METABOLISM** | | | | | |
| **P-value 0.00283** | **Count 7/68** | | | **10.3%** | |
| *BCAL0064* | *acoD* - Acetaldehyde dehydrogenase | | | 16.817 | |
| *BCAL1862* | *phbA* - Acetyl-CoA acetyltransferase | | | 1.852 | |
| *BCAL2122* | *aceB* - Malate synthase | | | 7.437 | |
| *BCAL2284* | *acoE* - Acetyl-CoA synthetase | | | 2.617 | |
| *BCAM0131* | *hchA* - Chaperone protein HchA | | | 4.096 | |
| *BCAM0187* | 2-isopropylmalate synthase | | | 8.786 | |
| *BCAM2372* | *acsA* - Acetyl-CoA synthetase | | | 19.709 | |
| **INOSITOL PHOSPHATE METABOLISM** | | | | | |
| **P-value 0.00294** | **Count 4/21** | | | **19.1%** | |
| *BCAL1427* | Myo-inositol catabolism protein | | | 11.695 | |
| *BCAL1428* | Putative amine catabolism-related protein | | | 13.589 | |
| *BCAL1429* | Putative TPP-binding acetolactate synthase | | | 30.290 | |
| *BCAL1435* | *idh* - Inositol 2-dehydrogenase | | | 15.127 | |
| **FATTY ACID METABOLISM** | | | | | |
| **P-value 0.002944** | **Count 6/51** | | | **11.8%** | |
| *BCAL0885* | Putative 3-hydroxyacyl-CoA dehydrogenase oxidoreductase | | | 2.019 | |
| *BCAL0886* | Acetyl-CoA acetyltransferase | | | 2.365 | |
| *BCAL1541* | Putative acyl-CoA synthetase | | | 7.385 | |
| *BCAL1862* | *phbA* - Acetyl-CoA acetyltransferase | | | 1.852 | |
| *BCAL1979* | Putative fatty acid degradation protein | | | 2.770 | |
| *BCAL3474* | *fadD* - Long-chain-fatty-acid-CoA ligase | | | 3.132 | |
| **GLYCOLYSIS / GLUCONEOGENESIS** | | | | | |
| **P-value 0.00584** | **Count 5/41** | | | **12.2%** | |
| *BCAL0064* | *acoD* - Acetaldehyde dehydrogenase | | | 16.817 | |
| *BCAL2284* | *acoE* - Acetyl-CoA synthetase | | | 2.617 | |
| *BCAL3044* | *glk* - Bifunctional glucokinase/RpiR family transcriptional regulator | | | 3.027 | |
| *BCAM2368* | Putative quinoprotein ethanol dehydrogenase precursor | | | 259.175 | |
| *BCAM2372* | *acsA* - Acetyl-CoA synthetase | | | 19.709 | |
| **C5-BRANCHED DIBASIC ACID METABOLISM** | | | | | |
| **P-value 0.00625** | **Count 3/13** | | | **23.1%** | |
| *BCAL2358* | *ilvH* - Acetolactate synthase 3 regulatory subunit | | | 2.692 | |
| *BCAM0983* | *leuC1* - Isopropylmalate isomerase large subunit | | | 2.395 | |
| *BCAM0984* | *leuD1* - Isopropylmalate isomerase small subunit | | | 3.423 | |
| **PHOSPHOTRANSFERASE SYSTEM (PTS)** | | | | | |
| **P-value 0.00988** | **Count 2/5** | | | **40.0%** | |
| *BCAL0781* | PTS system transporter subunit IIBC | | | 3.932 | |
| *BCAM0545* | Putative PTS transport system | | | 12.430 | |
| **ATRAZINE DEGRADATION** | | | | | |
| **P-value 0.01413** | **Count 2/6** | | **33.3%** | | |
| *BCAL3104* | *ureA* - Urease subunit gamma | | | 35.232 | |
| *BCAL3106* | *ureC* - Urease subunit alpha | | | 40.304 | |
| **BETA-ALANINE METABOLISM** | | | | | |
| **P-value 0.03684** | **Count 3/25** | | **12.0%** | | |
| *BCAM2561* | Putative 4-aminobutyrate aminotransferase | | | 2.388 | |
| *BCAS0731* | *dhT* - Phenylhydantoinase | | | 24.674 | |
| *BCAS0733* | Dihydropyrimidine dehydrogenase | | | 29.277 | |
| **GERANIOL DEGRADATION** | | | | | |
| **P-value 0.03747** | | **Count 2/10** | | | **20.0%** |
| *BCAL0884* | Putative acyl-CoA dehydrogenase oxidoreductase protein | | | 1.704 | |
| *BCAL0886* | Acetyl-CoA acetyltransferase | | | 2.365 | |
| **VALINE, LEUCINE, AND ISOLEUCINE DEGRADATION** | | | | | |
| **P-value 0.03999** | | **Count 4/46** | **8.7%** | | |
| *BCAL0886* | Acetyl-CoA acetyltransferase | | | 2.365 | |
| *BCAL1472* | *scoA* - Succinyl-CoA:3-ketoacid-coenzyme A transferase subunit A | | | 6.643 | |
| *BCAL1473* | *scoB* - Succinyl-CoA:3-ketoacid-coenzyme A transferase subunit B | | | 7.024 | |
| *BCAL1862* | *phbA* - Acetyl-CoA acetyltransferase | | | 1.852 | |
| **STREPTOMYCIN BIOSYNTHESIS** | | | | | |
| **P-value 0.04263** | **Cont 2/11** | | | **18.2%** | |
| *BCAL1435* | *idh* - Inositol 2-dehydrogenase | | | 15.127 | |
| *BCAL3044* | *glk* - Bifunctional glucokinase/RpiR family transcriptional regulator | | | 3.027 | |
| **GLYCEROPHOSPHOLIPID METABOLISM** | | | | | |
| **P-value 0.04874** | **Count 3/29** | | | **10.3%** | |
| *BCAL0059* | *eutB* - Ethanolamine ammonia-lyase heavy chain | | | 5.045 | |
| *BCAL0060* | *eutC* - Ethanolamine ammonia-lyase small subunit | | | 5.045 | |
| *BCAL0926* | *glpD* - Glycerol-3-phosphate dehydrogenase | | | 6.127 | |
| **BETA-LACTAM RESISTANCE** | | | | | |
| **P-value 0.07058** | **Count 3/34** | | | **8.8%** | |
| *BCAM1316* | Transport system outer membrane protein | | | 6.044 | |
| *BCAM2311* | Putative outer membrane porin protein | | | 8.904 | |
| *BCAM2728* | *oppA* - Putative periplasmic oligopeptide-binding protein precursor | | | 22.729 | |
| **GLYCEROLIPID METABOLISM** | | | | | |
| **P-value 0.07058** | **Count 2/15** | | | **13.3%** | |
| *BCAL0925* | *glpK* - Glycerol kinase | | | 10.076 | |
| *BCAM0949* | *lip* - exported lipase LipA | | | 7.761 | |
| **CHLOROALKANE AND CHLOROALKENE DEGRADATION** | | | | | |
| **P-value 0.08432** | **Count 2/17** | | | **11.8%** | |
| *BCAL2121* | Putative dehalogenase | | | 35.004 | |
| *BCAM2368* | Putative quinoprotein ethanol dehydrogenase precursor | | | 259.175 | |
| **RNA DEGRADATION** | | | | | |
| **P-value 0.08432** | **Count 2/17** | | | **11.8%** | |
| *BCAL1538* | Hfq protein | | | 2.523 | |
| *BCAM0548* | Chaperonin GroEL | | | 16.666 | |
| **METHANE METABOLISM** | | | | | |
| **P-value 0.08473** | **Count 3/38** | | | **7.9%** | |
| *BCAL2284* | *acoE* - Acetyl-CoA synthetase | | | 2.617 | |
| *BCAM2326* | Serine hydroxymethyltransferase | | | 23.996 | |
| *BCAM2372* | *acsA* - Acetyl-CoA synthetase | | | 19.709 | |
| **LYSINE DEGRADATION** | | | | | |
| **P-value 0.13614** | **Count 2/23** | | | **8.7%** | |
| *BCAL1862* | *phbA* - Acetyl-CoA acetyltransferase | | | 1.852 | |
| *BCAL3191* | Putative glutaryl-CoA dehydrogenase | | | 3.150 | |
| **OXIDATIVE PHOSPHORYLATION** | | | | | |
| **P-value 0.15917** | **Count 3/51** | | | **5.9%** | |
| *BCAL0328* | *petA* - Ubiquinol-cytochrome C reductase iron-sulfur subunit | | | 2.350 | |
| *BCAL0329* | *petB* - Cytochrome B; Component of the ubiquinol-cytochrome C reductase complex | | | 2.350 | |
| *BCAM0166* | NADH dehydrogenase | | | 6.810 | |
| **PENTOSE PHOSPHATE PATHWAY** | | | | | |
| **P-value 0.17608** | **Count 2/28** | | | **7.1%** | |
| *BCAL3043* | *pgl* - 6-phosphogluconolactonase | | | 3.027 | |
| *BCAL3366* | *eda* - KHG/KDPG aldolase | | | 3.340 | |
| **NICOTINATE AND NICOTINAMIDE METABOLISM** | | | | | |
| **P-value 0.17608** | **Count 2/28** | | | **7.1%** | |
| *BCAL3325* | *pntB* - NAD(P) transhydrogenase subunit beta | | | 4.404 | |
| *BCAL3326* | *pntAB* - NAD(P) transhydrogenase subunit alpha | | | 4.404 | |
| **CYSTEINE AND METHIONINE METABOLISM** | | | | | |
| **P-value 0.18232** | **Count 2/29** | | | **6.9%** | |
| *BCAM0540* | Putative serine acetyltransferase | | | 79.962 | |
| *BCAM0721* | O-acetylhomoserine (thiol)-lyase | | | 10.513 | |
| **CITRATE CYCLE (TCA CYCLE)** | | | | | |
| **P-value 0.20829** | **Count 2/32** | | | **6.3%** | |
| *BCAL2735* | Isocitrate dehydrogenase | | | 1.996 | |
| *BCAM1833* | *acnB* - Bifunctional aconitate hydratase 2/2-methylisocitrate dehydratase | | | 2.952 | |
| **TRYPTOPHAN METABOLISM** | | | | | |
| **P-value 0.25874** | **Count 2/38** | | | **5.3%** | |
| *BCAL1862* | *phbA* - Acetyl-CoA acetyltransferase | | | 1.852 | |
| *BCAL3191* | Putative glutaryl-CoA dehydrogenase | | | 3.150 | |
| **AMINO SUGAR AND NUCLEOTIDE SUGAR METABOLISM** | | | | | |
| **P-value 0.25874** | **Count 2/38** | | | **5.3%** | |
| *BCAL0781* | PTS system transporter subunit IIBC | | | 3.932 | |
| *BCAL3044* | *glk* - Bifunctional glucokinase/RpiR family transcriptional regulator | | | 3.027 | |
| **BENZOATE DEGRADATION** | | | | | |
| **P-value 0.33122** | **Count 2/49** | | | **4.1%** | |
| *BCAL0886* | Acetyl-CoA acetyltransferase | | | 2.365 | |
| *BCAL1862* | *phbA* - Acetyl-CoA acetyltransferase | | | 1.852 | |
| **PYRIMIDINE METABOLISM** | | | | | |
| **P-value 0.34380** | **Count 2/48** | | | **4.2%** | |
| *BCAS0731* | *dhT* - Phenylhydantoinase | | | 24.674 | |
| *BCAS0733* | Dihydropyrimidine dehydrogenase | | | 29.277 | |
| **BACTERIAL SECRETION SYSTEM** | | | | | |
| **P-value 0.36488** | **Count 2/51** | | | **3.9%** | |
| *BCAL3527* | *gspD* - Type II secretion system protein D | | | 2.103 | |
| *BCAM1316* | Transport system outer membrane protein | | | 6.044 | |
| **PURINE METABOLISM** | | | | | |
| **P-value 0.60920** | **Count 2/83** | | | **2.4%** | |
| *BCAL3104* | *ureA* - Urease subunit gamma | | | 35.232 | |
| *BCAL3106* | *ureC* - Urease subunit alpha | | | 40.304 | |

**Table S2**. List of genes down-regulated upon *B. cenocepacia* K56-2 adhesion (37ºC, 30min) to 16HBE14o- derived GPMVs. Genes were associated in Gene Ontology of KEGG pathway database obtained in ShinyGO v0.61 software. Enrichment analysis based on hypergeometric distribution followed by FDR correction^25,26,28^.

| **GENE** | **ANNOTATION OR PREDICTED FUNTION** | **FOLD CHANGE** |
| --- | --- | --- |
| **METABOLIC PATHWAYS** | | |
| **P-value 6.766e-59** | **Count 119/922** | **12.9%** |
| *BCAL0032* | *atpF* - F0F1 ATP synthase subunit B | -3.452 |
| *BCAL0033* | *atpH* - F0F1 ATP synthase subunit delta | -2.724 |
| *BCAL0034* | *atpA* - F0F1 ATP synthase subunit alpha | -3.732 |
| *BCAL0035* | *atpG* - F0F1 ATP synthase subunit gamma | -4.750 |
| *BCAL0036* | *atpD* - F0F1 ATP synthase subunit bet | -5.251 |
| *BCAL0037* | *atpC* - F0F1 ATP synthase subunit epsilon | -5.644 |
| *BCAL0073* | *gcvP* - Glycine dehydrogenase | -5.789 |
| *BCAL0075* | *gcvT* - Glycine cleavage system aminomethyltransferase T | -18.026 |
| *BCAL0145* | *ahcY* - S-adenosyl-L-homocysteine hydrolase | -3.820 |
| *BCAL0147* | *metF* - 5,10-methylenetetrahydrofolate reductase | -4.955 |
| *BCAL0226* | *rpoB* - DNA-directed RNA polymerase subunit beta | -3.774 |
| *BCAL0227* | *rpoC* - DNA-directed RNA polymerase subunit beta' | -8.193 |
| *BCAL0260* | *rpoA* - DNA-directed RNA polymerase subunit alpha | -2.506 |
| *BCAL0264* | *hemB* - Delta-aminolevulinic acid dehydratase | -2.457 |
| *BCAL0409* | *paaF* - Enoyl-CoA hydratase | -2.357 |
| *BCAL0422* | *dnaN* - DNA polymerase III subunit beta | -1.970 |
| *BCAL0433* | *speG* - spermidine N(1)-acetyltransferase | -4.072 |
| *BCAL0611* | *glmS1* - glucosamine--fructose-6-phosphate aminotransferase | -3.240 |
| *BCAL0705* | Putative D-amino acid aminotransferase | -2.748 |
| *BCAL0750* | *ctaD* - Cytochrome C oxidase polypeptide I | -2.084 |
| *BCAL0752* | Cytochrome C oxidase assembly protein | -3.376 |
| *BCAL0754* | Putative cytochrome C oxidase subunit III | -2.373 |
| *BCAL0784* | *cydB* - Cytochrome D ubiquinol oxidase subunit II | -14.401 |
| *BCAL0785* | *cydA* - Cytochrome D ubiquinol oxidase subunit I | -15.160 |
| *BCAL1059* | *argM* - Bifunctional N-succinyldiaminopimelate-aminotransferase/acetylornithine transaminase protein | -9.857 |
| *BCAL1212* | *bkdA1* - 2-oxoisovalerate dehydrogenase subunit alpha | -23.249 |
| *BCAL1213* | *bkdA2* - 2-oxoisovalerate dehydrogenase subunit beta | -27.571 |
| *BCAL1214* | *bkdB* - Branched-chain alpha-keto acid dehydrogenase subunit E2 | -37.308 |
| *BCAL1215* | *lpdV* - Dihydrolipoamide dehydrogenase | -20.612 |
| *BCAL1262* | *carB* - Carbamoyl phosphate synthase large subunit | -2.322 |
| *BCAL1413* | *glnS* - Glutaminyl-tRNA synthetase | -2.101 |
| *BCAL1467* | *aroC* - chorismate synthase | -3.171 |
| *BCAL1515* | *sucA* - 2-oxoglutarate dehydrogenase E1 component | -2.403 |
| *BCAL1516* | *sucB* - Dihydrolipoamide succinyltransferase | -3.499 |
| *BCAL1517* | *odhL* - Dihydrolipoamide dehydrogenase | -3.768 |
| *BCAL1711* | *cobN* - Cobaltochelatase subunit | -9.733 |
| *BCAL1712* | Putative magnesium chelatase protein | -9.733 |
| *BCAL1884* | *ispG* - 4-hydroxy-3-methylbut-2-en-1-yl diphosphate synthase | -2.679 |
| *BCAL1899* | *dnaX* - DNA polymerase III subunits gamma and tau | -2.607 |
| *BCAL1987* | *purL* - phosphoribosylformylglycinamidine synthase | -4.133 |
| *BCAL2061* | *guaA* - GMP synthase | -2.124 |
| *BCAL2063* | *guaB* - inosine 5'-monophosphate dehydrogenase | -2.454 |
| *BCAL2079* | *lpxA* - UDP-N-acetylglucosamine acyltransferase | -2.924 |
| *BCAL2080* | *fabZ* - (3R)-hydroxymyristoyl-ACP dehydratase | -3.301 |
| *BCAL2141* | *cyoD* - Cytochrome O ubiquinol oxidase protein | -15.188 |
| *BCAL2142* | *cyoC* - cytochrome o ubiquinol oxidase subunit III | -57.917 |
| *BCAL2143* | *cyoB* - ubiquinol oxidase polypeptide I | -57.917 |
| *BCAL2144* | *cyoA* - ubiquinol oxidase polypeptide II | -32.937 |
| *BCAL2207* | Putative dihydrolipoamide dehydrogenase | -6.063 |
| *BCAL2208* | *pdhB* - Dihydrolipoamide acetyltransferase | -7.683 |
| *BCAL2209* | *aceE* - Pyruvate dehydrogenase subunit E1 | -4.026 |
| *BCAL2244* | *hutU* - urocanate hydratase | -5.320 |
| *BCAL2303* | Aromatic amino acid aminotransferase | -2.049 |
| *BCAL2331* | *nuoN* - NADH dehydrogenase subunit N | -3.858 |
| *BCAL2332* | *nuoM* - NADH dehydrogenase subunit M | -3.858 |
| *BCAL2333* | *nuoL* - NADH dehydrogenase subunit L | -4.212 |
| *BCAL2334* | *nuoK* - NADH dehydrogenase subunit K | -3.903 |
| *BCAL2335* | *nuoJ* - NADH dehydrogenase subunit J | -3.648 |
| *BCAL2336* | *nuoI* - NADH dehydrogenase subunit I | -6.127 |
| *BCAL2337* | *nuoH* - NADH dehydrogenase subunit H | -5.679 |
| *BCAL2338* | NADH dehydrogenase subunit G | -6.929 |
| *BCAL2339* | *nuoF* - NADH dehydrogenase I chain F | -5.358 |
| *BCAL2340* | NADH dehydrogenase subunit E | -5.358 |
| *BCAL2341* | *nuoD* - NADH dehydrogenase subunit D | -4.833 |
| *BCAL2342* | *nuoC* - NADH dehydrogenase subunit C | -3.220 |
| *BCAL2343* | *nuoB* - NADH dehydrogenase subunit B | -4.355 |
| *BCAL2344* | *nuoA* - NADH dehydrogenase subunit A | -2.184 |
| *BCAL2433* | *tal* - Transaldolase B | -1.687 |
| *BCAL2638* | *argH* - Argininosuccinate lyase | -3.021 |
| *BCAL2782* | *pdxH* - Pyridoxamine 5'-phosphate oxidase | -4.034 |
| *BCAL2791* | *kynU* - Putative kynureninase | -14.681 |
| *BCAL2792* | *kynA* - Putative tryptophan 2,3-dioxygenase | -9.006 |
| *BCAL2908* | *fumC* - Fumarate hydratase | -4.369 |
| *BCAL2952* | *aroA* - 3-phosphoshikimate 1-carboxyvinyltransferase | -3.902 |
| *BCAL2955* | *serC* - Phosphoserine aminotransferase | -2.204 |
| *BCAL3094* | *hemN* - Coproporphyrinogen III oxidase | -36.590 |
| *BCAL3216* | *cysC* - Adenylyl-sulfate kinase | -2.024 |
| *BCAL3261* | *purM* - Phosphoribosylaminoimidazole synthetase | -2.290 |
| *BCAL3299* | *katG* - Peroxidase/catalase KatB | -4.895 |
| *BCAL3336* | purH - Bifunctional phosphoribosylaminoimidazolecarboxamide formyltransferase/IMP cyclohydrolase | -3.030 |
| *BCAL3359* | Putative glutamate dehydrogenase | -9.125 |
| *BCAL3361* | *purB* - Adenylosuccinate lyase | -2.545 |
| *BCAL3388* | *gapA* - Glyceraldehyde 3-phosphate dehydrogenase 1 | -4.547 |
| *BCAL3389* | *tktA* - Transketolase | -3.302 |
| *BCAL3425* | Putative sugar kinase | -3.336 |
| *BCAL3472* | *coq7* - 2-nonaprenyl-3-methyl-6-methoxy-1,4-benzoquinol hydroxylase | -9.819 |
| *BCAM0286* | Putative alcohol dehydrogenase | -17.616 |
| *BCAM0293* | *ackA* - Putative acetate kinase | -59.243 |
| *BCAM0298* | Putative phosphate acetyl/butyryl transferase | -76.193 |
| *BCAM0310* | Ribonucleotide reductase-like protein | -51.056 |
| *BCAM0311* | Putative 6-phosphofructokinase | -65.666 |
| *BCAM0911* | *dxs* - 1-deoxy-D-xylulose-5-phosphate synthase | -1.770 |
| *BCAM0961* | Aconitate hydratase | -2.465 |
| *BCAM0969* | *sdhA* - Succinate dehydrogenase flavoprotein subunit | -2.639 |
| *BCAM0970* | *sdhB* - Succinate dehydrogenase iron-sulfur subunit | -2.610 |
| *BCAM0972* | *gltA* - Type II citrate synthase | -2.215 |
| *BCAM0998* | *purF* - Amidophosphoribosyltransferase | -2.629 |
| *BCAM1111* | *speF* - Ornithine decarboxylase | -37.980 |
| *BCAM1112* | *adiA* - biodegradative arginine decarboxylase | -13.932 |
| *BCAM1204* | Alanine racemase | -15.392 |
| *BCAM1243* | Putative aminotransferase | -2.093 |
| *BCAM1245* | Putative phosphoenolpyruvate phosphomutase/sugar nucleotidyltransferase | -2.093 |
| *BCAM1250* | Putative acetyl-CoA hydrolase/transferase | -1.882 |
| *BCAM1570* | Alcohol dehydrogenase | -139.172 |
| *BCAM1573* | Alpha,alpha-trehalose-phosphate synthase | -2.273 |
| *BCAM1710* | Putative enoyl-CoA hydratase/isomerase | -11.823 |
| *BCAM2076* | *lysA* - Diaminopimelate decarboxylase | -2.864 |
| *BCAM2193* | Putative 3-hydroxyisobutyrate dehydrogenase | -6.309 |
| *BCAM2194* | *mmsA* - Methylmalonate-semialdehyde dehydrogenase | -4.876 |
| *BCAM2195* | Putative AMP-binding protein | -3.995 |
| *BCAM2430* | Putative biotin carboxylase | -15.417 |
| *BCAM2431* | Enoyl-CoA hydratase | -49.561 |
| *BCAM2432* | Putative biotin-dependent carboxyl transferase | -8.712 |
| *BCAM2433* | Putative acyl-CoA dehydrogenase | -5.801 |
| *BCAM2817* | Glycolate oxidase subunit GlcD | -9.100 |
| *BCAM2818* | Glycolate oxidase FAD binding subunit | -9.100 |
| *BCAS0737* | Putative acetyl-CoA acetyltransferase | -7.386 |
| *BCAS0739* | Putative acetyl-CoA synthetase | -5.646 |
| *BCAS0771* | Putative adenylosuccinate synthetase | -7.151 |
| **OXIDATIVE PHOSPHORYLATION** | | |
| **P-value 6.705e-38** | **Count 31/51** | **60.8%** |
| *BCAL0032* | *atpF* - F0F1 ATP synthase subunit B | -3.452 |
| *BCAL0033* | *atpH* - F0F1 ATP synthase subunit delta | -2.724 |
| *BCAL0034* | *atpA* - F0F1 ATP synthase subunit alpha | -3.732 |
| *BCAL0035* | *atpG* - F0F1 ATP synthase subunit gamma | -4.750 |
| *BCAL0036* | *atpD* - F0F1 ATP synthase subunit beta | -5.251 |
| *BCAL0037* | *atpC* - F0F1 ATP synthase subunit epsilon | -5.644 |
| *BCAL0750* | *ctaD* - Cytochrome C oxidase polypeptide I | -2.084 |
| *BCAL0752* | Cytochrome C oxidase assembly protein | -3.376 |
| *BCAL0754* | Putative cytochrome C oxidase subunit III | -2.373 |
| *BCAL0784* | *cydB* - Cytochrome D ubiquinol oxidase subunit II | -14.401 |
| *BCAL0785* | *cydA* - Cytochrome D ubiquinol oxidase subunit I | -15.160 |
| *BCAL2141* | *cyoD* - Cytochrome O ubiquinol oxidase protein | -15.188 |
| *BCAL2142* | *cyoC* - cytochrome O ubiquinol oxidase subunit III | -57.917 |
| *BCAL2143* | *cyoB* - Ubiquinol oxidase polypeptide I | -57.917 |
| *BCAL2144* | *cyoA* - Ubiquinol oxidase polypeptide II | -32.937 |
| *BCAL2331* | *nuoN* - NADH dehydrogenase subunit N | -3.858 |
| *BCAL2332* | *nuoM* - NADH dehydrogenase subunit M | -3.858 |
| *BCAL2333* | *nuoL* - NADH dehydrogenase subunit L | -4.212 |
| *BCAL2334* | *nuoK* - NADH dehydrogenase subunit K | -3.903 |
| *BCAL2335* | *nuoJ* - NADH dehydrogenase subunit J | -3.648 |
| *BCAL2336* | *nuoI* - NADH dehydrogenase subunit I | -6.127 |
| *BCAL2337* | *nuoH* - NADH dehydrogenase subunit H | -5.679 |
| *BCAL2338* | NADH dehydrogenase subunit G | -6.929 |
| *BCAL2339* | *nuoF* - NADH dehydrogenase I chain F | -5.358 |
| *BCAL2340* | NADH dehydrogenase subunit E | -5.358 |
| *BCAL2341* | *nuoD* - NADH dehydrogenase subunit D | -4.833 |
| *BCAL2342* | *nuoC* - NADH dehydrogenase subunit C | -3.220 |
| *BCAL2343* | *nuoB* - NADH dehydrogenase subunit B | -4.355 |
| *BCAL2344* | *nuoA* - NADH dehydrogenase subunit A | -2.184 |
| *BCAM0969* | *sdhA* - Succinate dehydrogenase flavoprotein subunit | -2.639 |
| *BCAM0970* | *sdhB* - Succinate dehydrogenase iron-sulfur subunit | -2.610 |
| **RIBOSOME** | | |
| **P-value 9.473e-31** | **Count 28/57** | **49.1%** |
| *BCAL0222* | *rplK* - 50S ribosomal protein L11 | -4.197 |
| *BCAL0223* | *rplA* - 50S ribosomal protein L1 | -3.883 |
| *BCAL0224* | *rplJ* - 50S ribosomal protein L10 | -5.670 |
| *BCAL0225* | *rplL* - 50S ribosomal protein L7/L12 | -5.924 |
| *BCAL0233* | *rpsJ* - 30S ribosomal protein S10 | -3.575 |
| *BCAL0234* | *rplC* - 50S ribosomal protein L3 | -9.099 |
| *BCAL0236* | *rplW* - 50S ribosomal protein L23 | -9.099 |
| *BCAL0237* | *rplB* - 50S ribosomal protein L2 | -14.352 |
| *BCAL0238* | *rpsS* - 30S ribosomal protein S19 | -16.178 |
| *BCAL0240* | *rpsC* - 30S ribosomal protein S3 | -21.137 |
| *BCAL0241* | *rplP* - 50S ribosomal protein L16 | -16.565 |
| *BCAL0242* | *rpmC* - 50S ribosomal protein L29 | -19.046 |
| *BCAL0243* | *rpsQ* - 30S ribosomal protein S17 | -19.046 |
| *BCAL0244* | *rplN* - 50S ribosomal protein L14 | -2.264 |
| *BCAL0246* | *rplE* - 50S ribosomal protein L5 | -2.706 |
| *BCAL0247* | *rpsN* - 30S ribosomal protein S14 | -3.100 |
| *BCAL0248* | *rpsH* - 30S ribosomal protein S8 | -3.343 |
| *BCAL0249* | *rplF* - 50S ribosomal protein L6 | -3.773 |
| *BCAL0250* | *rplR* - 50S ribosomal protein L18 | -4.580 |
| *BCAL0251* | *rpsE* - 30S ribosomal protein S5 | -4.181 |
| *BCAL0253* | *rplO* - 50S ribosomal protein L15 | -5.721 |
| *BCAL0257* | *rpsM* - 30S ribosomal protein S13 | -2.065 |
| *BCAL0258* | *rpsK* - 30S ribosomal protein S11 | -2.706 |
| *BCAL0259* | *rpsD* - 30S ribosomal protein S4 | -2.421 |
| *BCAL0799* | *rplY* - 50S ribosomal protein L25/general stress protein Ctc | -5.281 |
| *BCAL2925* | *rplS* - 50S ribosomal protein L19 | -4.795 |
| *BCAL2950* | *rpsA* - 30S ribosomal protein S1 | -1.848 |
| *BCAL3348* | *rplM* - 50S ribosomal protein L13 | -1.841 |
| **BIOSYNTHESIS OF SECONDARY METABOLITES** | | |
| **P-value 3.326e-27** | **Cout 52/359** | **14.5%** |
| *BCAL0264* | *hemB* - Delta-aminolevulinic acid dehydratase | -2.457 |
| *BCAL0409* | *paaF* - Enoyl-CoA hydratase | -2.357 |
| *BCAL0611* | *glmS1* - glucosamine--fructose-6-phosphate aminotransferase | -3.240 |
| *BCAL1059* | *argM* - Bifunctional N-succinyldiaminopimelate-aminotransferase/acetylornithine transaminase protein | -9.857 |
| *BCAL1212* | *bkdA1* - 2-oxoisovalerate dehydrogenase subunit alpha | -23.249 |
| *BCAL1213* | *bkdA2* - 2-oxoisovalerate dehydrogenase subunit beta | -27.571 |
| *BCAL1214* | *bkdB* - Branched-chain alpha-keto acid dehydrogenase subunit E2 | -37.308 |
| *BCAL1215* | *lpdV* - Dihydrolipoamide dehydrogenase | -20.612 |
| *BCAL1467* | *aroC* - chorismate synthase | -3.171 |
| *BCAL1515* | *sucA* - 2-oxoglutarate dehydrogenase E1 component | -2.403 |
| *BCAL1516* | *sucB* - Dihydrolipoamide succinyltransferase | -3.499 |
| *BCAL1517* | *odhL* - Dihydrolipoamide dehydrogenase | -3.768 |
| *BCAL1712* | Putative magnesium chelatase protein | -9.733 |
| *BCAL1722* | Putative exported chitinase | -9.498 |
| *BCAL1884* | *ispG* - 4-hydroxy-3-methylbut-2-en-1-yl diphosphate synthase | -2.679 |
| *BCAL1987* | *purL* - phosphoribosylformylglycinamidine synthase | -4.133 |
| *BCAL2063* | *guaB* - inosine 5'-monophosphate dehydrogenase | -2.454 |
| *BCAL2207* | Putative dihydrolipoamide dehydrogenase | -6.063 |
| *BCAL2208* | *pdhB* - Dihydrolipoamide acetyltransferase | -7.683 |
| *BCAL2209* | *aceE* - Pyruvate dehydrogenase subunit E1 | -4.026 |
| *BCAL2303* | Aromatic amino acid aminotransferase | -2.049 |
| *BCAL2433* | *tal* - Transaldolase B | -1.687 |
| *BCAL2638* | *argH* - Argininosuccinate lyase | -3.021 |
| *BCAL2908* | *fumC* - Fumarate hydratase | -4.369 |
| *BCAL2952* | *aroA* - 3-phosphoshikimate 1-carboxyvinyltransferase | -3.902 |
| *BCAL3094* | *hemN* - Coproporphyrinogen III oxidase | -36.590 |
| *BCAL3261* | *purM* - Phosphoribosylaminoimidazole synthetase | -2.290 |
| *BCAL3336* | purH - Bifunctional phosphoribosylaminoimidazolecarboxamide formyltransferase/IMP cyclohydrolase | -3.030 |
| *BCAL3361* | *purB* - Adenylosuccinate lyase | -2.545 |
| *BCAL3388* | *gapA* - Glyceraldehyde 3-phosphate dehydrogenase 1 | -4.547 |
| *BCAL3389* | *tktA* - Transketolase | -3.302 |
| *BCAL3472* | *coq7* - 2-nonaprenyl-3-methyl-6-methoxy-1,4-benzoquinol hydroxylase | -9.819 |
| *BCAM0286* | Putative alcohol dehydrogenase | -17.616 |
| *BCAM0311* | Putative 6-phosphofructokinase | -65.666 |
| *BCAM0911* | *dxs* - 1-deoxy-D-xylulose-5-phosphate synthase | -1.770 |
| *BCAM0961* | Aconitate hydratase | -2.465 |
| *BCAM0969* | *sdhA* - Succinate dehydrogenase flavoprotein subunit | -2.639 |
| *BCAM0970* | *sdhB* - Succinate dehydrogenase iron-sulfur subunit | -2.610 |
| *BCAM0972* | *gltA* - Type II citrate synthase | -2.215 |
| *BCAM0998* | *purF* - Amidophosphoribosyltransferase | -2.629 |
| *BCAM1111* | *speF* - Ornithine decarboxylase | -37.980 |
| *BCAM1245* | Putative phosphoenolpyruvate phosphomutase/sugar nucleotidyltransferase | -2.093 |
| *BCAM1250* | Putative acetyl-CoA hydrolase/transferase | -1.882 |
| *BCAM1570* | Alcohol dehydrogenase | -139.172 |
| *BCAM2076* | *lysA* - Diaminopimelate decarboxylase | -2.864 |
| *BCAM2195* | Putative AMP-binding protein | -3.995 |
| *BCAM2196* | Putative acyl-CoA dehydrogenase | -5.913 |
| *BCAM2817* | Glycolate oxidase subunit GlcD | -9.100 |
| *BCAM2818* | Glycolate oxidase FAD binding subunit | -9.100 |
| *BCAM2831* | Squlalene--hopene cyclase | -3.428 |
| *BCAS0737* | Putative acetyl-CoA acetyltransferase | -7.386 |
| *BCAS0739* | Putative acetyl-CoA synthetase | -5.646 |
| **MICROBIAL METABOLISM IN DIVERSE ENVIRONMENTS** | | |
| **P-value 7.388e-22** | **Count 48/393** | **12.2%** |
| *BCAL0147* | *metF* - 5,10-methylenetetrahydrofolate reductase | -4.955 |
| *BCAL0212* | *paaE* - Putative phenylacetic acid degradation NADH oxidoreductase PaaE | -43.665 |
| *BCAL0213* | *paaD* - Phenylacetic acid degradation protein PaaD | -44.213 |
| *BCAL0214* | *paaC* - Phenylacetic acid degradation protein PaaC | -33.710 |
| *BCAL0215* | *paaB* - Phenylacetate-CoA oxygenase subunit PaaB | -36.644 |
| *BCAL0216* | *paaA* - Phenylacetate-CoA oxygenase subunit PaaA | -34.435 |
| *BCAL0406* | *paaG* - Enoyl-CoA hydratase | -11.166 |
| *BCAL0409* | *paaF* - Enoyl-CoA hydratase | -2.357 |
| *BCAL1059* | *argM* - Bifunctional N-succinyldiaminopimelate-aminotransferase/acetylornithine transaminase protein | -9.857 |
| *BCAL1215* | *lpdV* - Dihydrolipoamide dehydrogenase | -20.612 |
| *BCAL1515* | *sucA* - 2-oxoglutarate dehydrogenase E1 component | -2.403 |
| *BCAL1516* | *sucB* - Dihydrolipoamide succinyltransferase | -3.499 |
| *BCAL1517* | *odhL* - Dihydrolipoamide dehydrogenase | -3.768 |
| *BCAL2207* | Putative dihydrolipoamide dehydrogenase | -6.063 |
| *BCAL2208* | *pdhB* - Dihydrolipoamide acetyltransferase | -7.683 |
| *BCAL2209* | *aceE* - Pyruvate dehydrogenase subunit E1 | -4.026 |
| *BCAL2433* | *tal* - Transaldolase B | -1.687 |
| *BCAL2908* | *fumC* - Fumarate hydratase | -4.369 |
| *BCAL2955* | *serC* - Phosphoserine aminotransferase | -2.204 |
| *BCAL3179* | *ldhA* - Putative D-lactate dehydrogenase | -2.995 |
| *BCAL3216* | *cysC* - Adenylyl-sulfate kinase | -2.024 |
| *BCAL3299* | *katG* - Peroxidase/catalase KatB | -4.895 |
| *BCAL3388* | *gapA* - Glyceraldehyde 3-phosphate dehydrogenase 1 | -4.547 |
| *BCAL3389* | *tktA* - Transketolase | -3.302 |
| *BCAM0286* | Putative alcohol dehydrogenase | -17.616 |
| *BCAM0293* | *ackA* - Putative acetate kinase | -59.243 |
| *BCAM0296* | Acetoacetyl-CoA reductase | -76.193 |
| *BCAM0298* | Putative phosphate acetyl/butyryl transferase | -76.193 |
| *BCAM0311* | Putative 6-phosphofructokinase | -65.666 |
| *BCAM0810* | Putative aromatic oxygenase | -10.983 |
| *BCAM0811* | Putative aromatic oxygenase | -6.831 |
| *BCAM0961* | Aconitate hydratase | -2.465 |
| *BCAM0969* | *sdhA* - Succinate dehydrogenase flavoprotein subunit | -2.639 |
| *BCAM0970* | *sdhB* - Succinate dehydrogenase iron-sulfur subunit | -2.610 |
| *BCAM0972* | *gltA* - Type II citrate synthase | -2.215 |
| *BCAM1243* | Putative aminotransferase | -2.093 |
| *BCAM1245* | Putative phosphoenolpyruvate phosphomutase/sugar nucleotidyltransferase | -2.093 |
| *BCAM1250* | Putative acetyl-CoA hydrolase/transferase | -1.882 |
| *BCAM1570* | Alcohol dehydrogenase | -139.172 |
| *BCAM1711* | Phenylacetate-coenzyme A ligase | -11.823 |
| *BCAM2076* | *lysA* - Diaminopimelate decarboxylase | -2.864 |
| *BCAM2191* | Enoyl-CoA hydratase/isomerase family | -6.309 |
| *BCAM2192* | Enoyl-CoA hydratase | -6.309 |
| *BCAM2195* | Putative AMP-binding protein | -3.995 |
| *BCAM2817* | Glycolate oxidase subunit GlcD | -9.100 |
| *BCAM2818* | Glycolate oxidase FAD binding subunit | -9.100 |
| *BCAS0737* | Putative acetyl-CoA acetyltransferase | -7.386 |
| *BCAS0739* | Putative acetyl-CoA synthetase | -5.646 |
| **CARBON METABOLISM** | | |
| **P-value 7.871e-18** | **Count 27/136** | **19.9%** |
| *BCAL0073* | *gcvP* - Glycine dehydrogenase | -5.789 |
| *BCAL0075* | *gcvT* - Glycine cleavage system aminomethyltransferase T | -18.026 |
| *BCAL0147* | *metF* - 5,10-methylenetetrahydrofolate reductase | -4.955 |
| *BCAL1215* | *lpdV* - Dihydrolipoamide dehydrogenase | -20.612 |
| *BCAL1515* | *sucA* - 2-oxoglutarate dehydrogenase E1 component | -2.403 |
| *BCAL1516* | *sucB* - Dihydrolipoamide succinyltransferase | -3.499 |
| *BCAL1517* | *odhL* - Dihydrolipoamide dehydrogenase | -3.768 |
| *BCAL2207* | Putative dihydrolipoamide dehydrogenase | -6.063 |
| *BCAL2208* | *pdhB* - Dihydrolipoamide acetyltransferase | -7.683 |
| *BCAL2209* | *aceE* - Pyruvate dehydrogenase subunit E1 | -4.026 |
| *BCAL2433* | *tal* - Transaldolase B | -1.687 |
| *BCAL2908* | *fumC* - Fumarate hydratase | -4.369 |
| *BCAL2955* | *serC* - Phosphoserine aminotransferase | -2.204 |
| *BCAL3388* | *gapA* - Glyceraldehyde 3-phosphate dehydrogenase 1 | -4.547 |
| *BCAL3389* | *tktA* - Transketolase | -3.302 |
| *BCAM0293* | *ackA* - Putative acetate kinase | -59.243 |
| *BCAM0298* | Putative phosphate acetyl/butyryl transferase | -76.193 |
| *BCAM0311* | Putative 6-phosphofructokinase | -65.666 |
| *BCAM0961* | Aconitate hydratase | -2.465 |
| *BCAM0969* | *sdhA* - Succinate dehydrogenase flavoprotein subunit | -2.639 |
| *BCAM0970* | *sdhB* - Succinate dehydrogenase iron-sulfur subunit | -2.610 |
| *BCAM0972* | *gltA* - Type II citrate synthase | -2.215 |
| *BCAM1250* | Putative acetyl-CoA hydrolase/transferase | -1.882 |
| *BCAM2194* | *mmsA* - Methylmalonate-semialdehyde dehydrogenase | -4.876 |
| *BCAM2195* | Putative AMP-binding protein | -3.995 |
| *BCAS0737* | Putative acetyl-CoA acetyltransferase | -7.386 |
| *BCAS0739* | Putative acetyl-CoA synthetase | -5.646 |
| **VALINE, LEUCINE, AND ISOLEUCINE DEGRADATION** | | |
| **P-value 1.500e-13** | **Count 15/46** | **32.6%** |
| *BCAL0409* | *paaF* - Enoyl-CoA hydratase | -2.357 |
| *BCAL1212* | *bkdA1* - 2-oxoisovalerate dehydrogenase subunit alpha | -23.249 |
| *BCAL1213* | *bkdA2* - 2-oxoisovalerate dehydrogenase subunit beta | -27.571 |
| *BCAL1214* | *bkdB* - Branched-chain alpha-keto acid dehydrogenase subunit E2 | -37.308 |
| *BCAL1215* | *lpdV* - Dihydrolipoamide dehydrogenase | -20.612 |
| *BCAL1517* | *odhL* - Dihydrolipoamide dehydrogenase | -3.768 |
| *BCAL2207* | Putative dihydrolipoamide dehydrogenase | -6.063 |
| *BCAM1710* | Putative enoyl-CoA hydratase/isomerase | -11.823 |
| *BCAM2193* | Putative 3-hydroxyisobutyrate dehydrogenase | -6.309 |
| *BCAM2194* | *mmsA* - Methylmalonate-semialdehyde dehydrogenase | -4.876 |
| *BCAM2430* | Putative biotin carboxylase | -15.417 |
| *BCAM2431* | Enoyl-CoA hydratase | -49.561 |
| *BCAM2432* | Putative biotin-dependent carboxyl transferase | -8.712 |
| *BCAM2433* | Putative acyl-CoA dehydrogenase | -5.801 |
| *BCAS0737* | Putative acetyl-CoA acetyltransferase | -7.386 |
| **CITRATE CYCLE (TCA CYCLE)** | | |
| **P-value 2.681e-13** | **Count 13/32** | **40.6%** |
| *BCAL1215* | *lpdV* - Dihydrolipoamide dehydrogenase | -20.612 |
| *BCAL1515* | *sucA* - 2-oxoglutarate dehydrogenase E1 component | -2.403 |
| *BCAL1516* | *sucB* - Dihydrolipoamide succinyltransferase | -3.499 |
| *BCAL1517* | *odhL* - Dihydrolipoamide dehydrogenase | -3.768 |
| *BCAL2207* | Putative dihydrolipoamide dehydrogenase | -6.063 |
| *BCAL2208* | *pdhB* - Dihydrolipoamide acetyltransferase | -7.683 |
| *BCAL2209* | *aceE* - Pyruvate dehydrogenase subunit E1 | -4.026 |
| *BCAL2908* | *fumC* - Fumarate hydratase | -4.369 |
| *BCAM0961* | Aconitate hydratase | -2.465 |
| *BCAM0969* | *sdhA* - Succinate dehydrogenase flavoprotein subunit | -2.639 |
| *BCAM0970* | *sdhB* - Succinate dehydrogenase iron-sulfur subunit | -2.610 |
| *BCAM0972* | *gltA* - Type II citrate synthase | -2.215 |
| *BCAM1250* | Putative acetyl-CoA hydrolase/transferase | -1.882 |
| **PURINE METABOLISM** | | |
| **P-value 7.431e-13** | **Count 18/83** | **21.7%** |
| *BCAL0226* | *rpoB* - DNA-directed RNA polymerase subunit beta | -3.774 |
| *BCAL0227* | *rpoC* - DNA-directed RNA polymerase subunit beta' | -8.193 |
| *BCAL0260* | *rpoA* - DNA-directed RNA polymerase subunit alpha | -2.506 |
| *BCAL0422* | *dnaN* - DNA polymerase III subunit beta | -1.970 |
| *BCAL1899* | *dnaX* - DNA polymerase III subunits gamma and tau | -2.607 |
| *BCAL1987* | *purL* - phosphoribosylformylglycinamidine synthase | -4.133 |
| *BCAL2061* | *guaA* - GMP synthase | -2.124 |
| *BCAL2063* | *guaB* - inosine 5'-monophosphate dehydrogenase | -2.454 |
| *BCAL2329* | NUDIX hydrolase | -3.504 |
| *BCAL2348* | *pnp* - Polynucleotide phosphorylase/polyadenylase | -1.936 |
| *BCAL3216* | *cysC* - Adenylyl-sulfate kinase | -2.024 |
| *BCAL3261* | *purM* - Phosphoribosylaminoimidazole synthetase | -2.290 |
| *BCAL3336* | purH - Bifunctional phosphoribosylaminoimidazolecarboxamide formyltransferase/IMP cyclohydrolase | -3.030 |
| *BCAL3361* | *purB* - Adenylosuccinate lyase | -2.545 |
| *BCAL3425* | Putative sugar kinase | -3.336 |
| *BCAM0310* | Ribonucleotide reductase-like protein | -51.056 |
| *BCAM0998* | *purF* - Amidophosphoribosyltransferase | -2.629 |
| *BCAS0771* | Putative adenylosuccinate synthetase | -7.151 |
| **BACTERIAL CHEMOTAXIS** | | |
| **P-value 1.414e-10** | **Count 13/40** | **32.5%** |
| *BCAL0126* | *motA* - Flagellar motor protein MotA | -1.913 |
| *BCAL0129* | *cheA* - Chemotaxis two-component sensor kinase CheA | -2.496 |
| *BCAL0130* | *cheW* - Chemotaxis protein CheW | -2.889 |
| *BCAL0131* | *tar* - Methyl-accepting chemotaxis protein | -3.261 |
| *BCAL0132* | *cheR* - Chemotaxis protein methyltransferase | -2.324 |
| *BCAL0133* | *cheD* - Chemoreceptor glutamine deamidase CheD | -2.324 |
| *BCAL0134* | *cheB1* - Chemotaxis-specific methylesterase | -2.324 |
| *BCAL0135* | Chemotaxis protein CheY | -3.744 |
| *BCAL0762* | Putative methyl-accepting chemotaxis protein | -5.099 |
| *BCAM1503* | Putative methyl-accepting chemotaxis protein | -4.184 |
| *BCAM1572* | Methyl-accepting chemotaxis protein | -2.811 |
| *BCAM1804* | Methyl-accepting chemotaxis protein | -12.383 |
| *BCAM2564* | *aer* - Putative aerotaxis receptor | -3.607 |
| **PYRUVATE METABOLISM** | | |
| **P-value 7.225e-10** | **Count 14/68** | **20.6%** |
| *BCAL1215* | *lpdV* - Dihydrolipoamide dehydrogenase | -20.612 |
| *BCAL1517* | *odhL* - Dihydrolipoamide dehydrogenase | -3.768 |
| *BCAL2207* | Putative dihydrolipoamide dehydrogenase | -6.063 |
| *BCAL2208* | *pdhB* - Dihydrolipoamide acetyltransferase | -7.683 |
| *BCAL2209* | *aceE* - Pyruvate dehydrogenase subunit E1 | -4.026 |
| *BCAL2908* | *fumC* - Fumarate hydratase | -4.369 |
| *BCAL3179* | *ldhA* - Putative D-lactate dehydrogenase | -2.995 |
| *BCAM0293* | *ackA* - Putative acetate kinase | -59.243 |
| *BCAM0298* | Putative phosphate acetyl/butyryl transferase | -76.193 |
| *BCAM0387* | *poxB*- Pyruvate dehydrogenase | -2.387 |
| *BCAM1250* | Putative acetyl-CoA hydrolase/transferase | -1.882 |
| *BCAM2195* | Putative AMP-binding protein | -3.995 |
| *BCAS0737* | Putative acetyl-CoA acetyltransferase | -7.386 |
| *BCAS0739* | Putative acetyl-CoA synthetase | -5.646 |
| **TWO-COMPONENT SYSTEM** | | |
| **P-value 1.983e-09** | **Count 18/132** | **13.6%** |
| *BCAL0114* | *fliC* - Flagellin | -1.921 |
| *BCAL0126* | *motA* - Flagellar motor protein MotA | -1.913 |
| *BCAL0129* | *cheA* - Chemotaxis two-component sensor kinase CheA | -2.496 |
| *BCAL0130* | *cheW* - Chemotaxis protein CheW | -2.889 |
| *BCAL0132* | *cheR* - Chemotaxis protein methyltransferase | -2.324 |
| *BCAL0134* | *cheB1* - Chemotaxis-specific methylesterase | -2.324 |
| *BCAL0135* | Chemotaxis protein CheY | -3.744 |
| *BCAL0499* | Two-component regulatory system, response regulator protein | -4.190 |
| *BCAL0762* | Putative methyl-accepting chemotaxis protein | -5.099 |
| *BCAL0784* | *cydB* - Cytochrome D ubiquinol oxidase subunit II | -14.401 |
| *BCAL0785* | *cydA* - Cytochrome D ubiquinol oxidase subunit I | -15.160 |
| *BCAL1674* | *amrA* - Periplasmic multidrug efflux lipoprotein | -7.862 |
| *BCAL1675* | *amrB* - Multidrug efflux protein | -6.194 |
| *BCAM1503* | Putative methyl-accepting chemotaxis protein | -4.184 |
| *BCAM1572* | Methyl-accepting chemotaxis protein | -2.811 |
| *BCAM1804* | Methyl-accepting chemotaxis protein | -12.383 |
| *BCAM2564* | *aer* - Putative aerotaxis receptor | -3.607 |
| *BCAS0737* | Putative acetyl-CoA acetyltransferase | -7.386 |
| **GLYCOLYSIS / GLUCONEOGENESIS** | | |
| **P-value 2.881e-09** | **Count 11/41** | **28.8%** |
| *BCAL1215* | *lpdV* - Dihydrolipoamide dehydrogenase | -20.612 |
| *BCAL1517* | *odhL* - Dihydrolipoamide dehydrogenase | -3.768 |
| *BCAL2207* | Putative dihydrolipoamide dehydrogenase | -6.063 |
| *BCAL2208* | *pdhB* - Dihydrolipoamide acetyltransferase | -7.683 |
| *BCAL2209* | *aceE* - Pyruvate dehydrogenase subunit E1 | -4.026 |
| *BCAL3388* | *gapA* - Glyceraldehyde 3-phosphate dehydrogenase 1 | -4.547 |
| *BCAM0286* | Putative alcohol dehydrogenase | -17.616 |
| *BCAM0311* | Putative 6-phosphofructokinase | -65.666 |
| *BCAM1570* | Alcohol dehydrogenase | -139.172 |
| *BCAM2195* | Putative AMP-binding protein | -3.995 |
| *BCAS0739* | Putative acetyl-CoA synthetase | -5.646 |
| **PHENYLALANINE METABOLISM** | | |
| **P-value 1.408e-08** | **Count 12/59** | **20.3%** |
| *BCAL0212* | *paaE* - Putative phenylacetic acid degradation NADH oxidoreductase PaaE | -43.665 |
| *BCAL0213* | *paaD* - Phenylacetic acid degradation protein PaaD | -44.213 |
| *BCAL0214* | *paaC* - Phenylacetic acid degradation protein PaaC | -33.710 |
| *BCAL0215* | *paaB* - Phenylacetate-CoA oxygenase subunit PaaB | -36.644 |
| *BCAL0216* | *paaA* - Phenylacetate-CoA oxygenase subunit PaaA | -34.435 |
| *BCAL0406* | *paaG* - Enoyl-CoA hydratase | -11.166 |
| *BCAL0409* | *paaF* - Enoyl-CoA hydratase | -2.357 |
| *BCAL0705* | Putative D-amino acid aminotransferase | -2.748 |
| *BCAL2303* | Aromatic amino acid aminotransferase | -2.049 |
| *BCAL2933* | *dada* - D-amino acid dehydrogenase small subunit | -215.050 |
| *BCAL3299* | *katG* - Peroxidase/catalase KatB | -4.895 |
| *BCAM1711* | Phenylacetate-coenzyme A ligase | -11.823 |
| **GLYCINE, SERINE AND THREONINE METABOLISM** | | |
| **P-value 1.027e-05** | **Count 9/56** | **16.1%** |
| *BCAL0073* | *gcvP* - Glycine dehydrogenase | -5.789 |
| *BCAL0075* | *gcvT* - Glycine cleavage system aminomethyltransferase T | -18.026 |
| *BCAL1215* | *lpdV* - Dihydrolipoamide dehydrogenase | -20.612 |
| *BCAL1517* | *odhL* - Dihydrolipoamide dehydrogenase | -3.768 |
| *BCAL2207* | Putative dihydrolipoamide dehydrogenase | -6.063 |
| *BCAL2955* | *serC* - Phosphoserine aminotransferase | -2.204 |
| *BCAM0010* | *kbl* - 2-amino-3-ketobutyrate coenzyme A ligase | -8.746 |
| *BCAM0011* | *tdh* - L-threonine 3-dehydrogenase | -6.223 |
| *BCAM0908* | Putative iron-sulfur protein | -3.280 |
| **PYRIMIDINE METABOLISM** | | |
| **P-value 2.654e-05** | **Count 8/48** | **16.7%** |
| *BCAL0226* | *rpoB* - DNA-directed RNA polymerase subunit beta | -3.774 |
| *BCAL0227* | *rpoC* - DNA-directed RNA polymerase subunit beta' | -8.193 |
| *BCAL0260* | *rpoA* - DNA-directed RNA polymerase subunit alpha | -2.506 |
| *BCAL0422* | *dnaN* - DNA polymerase III subunit beta | -1.970 |
| *BCAL1262* | *carB* - Carbamoyl phosphate synthase large subunit | -2.322 |
| *BCAL1899* | *dnaX* - DNA polymerase III subunits gamma and tau | -2.607 |
| *BCAL2348* | *pnp* - Polynucleotide phosphorylase/polyadenylase | -1.936 |
| *BCAM0310* | Ribonucleotide reductase-like protein | -51.056 |
| **PROPANOATE METABOLISM** | | |
| **P-value 2.930e-05** | **Count 8/49** | **16.3%** |
| *BCAL0409* | *paaF* - Enoyl-CoA hydratase | -2.357 |
| *BCAM0293* | *ackA* - Putative acetate kinase | -59.243 |
| *BCAM0298* | Putative phosphate acetyl/butyryl transferase | -76.193 |
| *BCAM0299* | Putative zinc-binding alcoholdehydrogenase | -76.193 |
| *BCAM2194* | *mmsA* - Methylmalonate-semialdehyde dehydrogenase | -4.876 |
| *BCAM2195* | Putative AMP-binding protein | -3.995 |
| *BCAS0737* | Putative acetyl-CoA acetyltransferase | -7.386 |
| *BCAS0739* | Putative acetyl-CoA synthetase | -5.646 |
| **ALANINE, ASPARTATE AND GLUTAMATE METABOLISM** | | |
| **P-value 3.751e-05** | **Count 7/37** | **18.9%** |
| *BCAL0611* | *glmS1* - glucosamine--fructose-6-phosphate aminotransferase | -3.240 |
| *BCAL1262* | *carB* - Carbamoyl phosphate synthase large subunit | -2.322 |
| *BCAL2638* | *argH* - Argininosuccinate lyase | -3.021 |
| *BCAL3359* | Putative glutamate dehydrogenase | -9.125 |
| *BCAL3361* | *purB* - Adenylosuccinate lyase | -2.545 |
| *BCAM0998* | *purF* - Amidophosphoribosyltransferase | -2.629 |
| *BCAS0771* | Putative adenylosuccinate synthetase | -7.151 |
| **BIOSYNTHESIS OF AMINO ACIDS** | | |
| **P-value 5.790e-05** | **Count 13/149** | **8.7%** |
| *BCAL1059* | *argM* - Bifunctional N-succinyldiaminopimelate-aminotransferase/acetylornithine transaminase protein | -9.857 |
| *BCAL1467* | *aroC* - chorismate synthase | -3.171 |
| *BCAL2303* | Aromatic amino acid aminotransferase | -2.049 |
| *BCAL2433* | *tal* - Transaldolase B | -1.687 |
| *BCAL2638* | *argH* - Argininosuccinate lyase | -3.021 |
| *BCAL2952* | *aroA* - 3-phosphoshikimate 1-carboxyvinyltransferase | -3.902 |
| *BCAL2955* | *serC* - Phosphoserine aminotransferase | -2.204 |
| *BCAL3388* | *gapA* - Glyceraldehyde 3-phosphate dehydrogenase 1 | -4.547 |
| *BCAL3389* | *tktA* - Transketolase | -3.302 |
| *BCAM0311* | Putative 6-phosphofructokinase | -65.666 |
| *BCAM0961* | Aconitate hydratase | -2.465 |
| *BCAM0972* | *gltA* - Type II citrate synthase | -2.215 |
| *BCAM2076* | *lysA* - Diaminopimelate decarboxylase | -2.864 |
| **ARGININE AND PROLINE METABOLISM** | | |
| **P-value 6.220e-05** | **Count 9/72** | **12.5%** |
| *BCAL0433* | *speG* - spermidine N(1)-acetyltransferase | -4.072 |
| *BCAL0705* | Putative D-amino acid aminotransferase | -2.748 |
| *BCAL1059* | *argM* - Bifunctional N-succinyldiaminopimelate-aminotransferase/acetylornithine transaminase protein | -9.857 |
| *BCAL1062* | *astD* - Succinylglutamic semialdehyde dehydrogenase | -5.393 |
| *BCAL1063* | *astB* - Succinylarginine dihydrolase | -6.067 |
| *BCAL2638* | *argH* - Argininosuccinate lyase | -3.021 |
| *BCAL3359* | Putative glutamate dehydrogenase | -9.125 |
| *BCAM1111* | *speF* - Ornithine decarboxylase | -37.980 |
| *BCAM1112* | *adiA* - biodegradative arginine decarboxylase | -13.932 |
| **PROTEIN EXPORT** | | |
| **P-value 6.220e-05** | **Count 5/17** | **29.4%** |
| *BCAL0254* | *secY* - Preprotein translocase subunit SecY | -4.083 |
| *BCAL0742* | *secB* - Preprotein translocase subunit SecB | -1.928 |
| *BCAL3307* | *secF* - Preprotein translocase subunit SecF | -5.013 |
| *BCAL3433* | *ffh* - Signal recognition particle protein | -2.793 |
| *BCAL3453* | *secA* - Preprotein translocase subunit SecA | -2.055 |
| **RNA POLYMERASE** | | |
| **P-value 0.00011** | **Count 3/4** | **75.0%** |
| *BCAL0226* | *rpoB* - DNA-directed RNA polymerase subunit beta | -3.774 |
| *BCAL0227* | *rpoC* - DNA-directed RNA polymerase subunit beta' | -8.193 |
| *BCAL0260* | *rpoA* - DNA-directed RNA polymerase subunit alpha | -2.506 |
| **AMINOACYL-tRNA BIOSYNTHESIS** | | |
| **P-value 0.00011** | **Count 6/31** | **19.4%** |
| *BCAL0880* | *aspS* - Aspartyl-tRNA synthetase | -2.328 |
| *BCAL1413* | *glnS* - Glutaminyl-tRNA synthetase | -2.101 |
| *BCAL1448* | *valS* - Valyl-tRNA synthetase | -3.825 |
| *BCAL1486* | *pheT* - Phenylalanyl-tRNA synthetase subunit beta | -2.593 |
| *BCAL1883* | *hisS* - Histidyl-tRNA synthetase | -2.380 |
| *BCAL2169* | *trpS* - Tryptophanyl-tRNA synthetase | -3.423 |
| **LYSINE DEGRADATION** | | |
| **P-value 0.00027** | **Count 5/23** | **27.7%** |
| *BCAL0409* | *paaF* - Enoyl-CoA hydratase | -2.357 |
| *BCAL0705* | Putative D-amino acid aminotransferase | -2.748 |
| *BCAL1515* | *sucA* - 2-oxoglutarate dehydrogenase E1 component | -2.403 |
| *BCAL1516* | *sucB* - Dihydrolipoamide succinyltransferase | -3.499 |
| *BCAS0737* | Putative acetyl-CoA acetyltransferase | -7.386 |
| **TRYPTOPHAN METABOLISM** | | |
| **P-value 0.00033** | **Count 6/38** | **15.8%** |
| *BCAL0409* | *paaF* - Enoyl-CoA hydratase | -2.357 |
| *BCAL1515* | *sucA* - 2-oxoglutarate dehydrogenase E1 component | -2.403 |
| *BCAL2791* | *kynU* - Putative kynureninase | -14.681 |
| *BCAL2792* | *kynA* - Putative tryptophan 2,3-dioxygenase | -9.006 |
| *BCAL3299* | *katG* - Peroxidase/catalase KatB | -4.895 |
| *BCAS0737* | Putative acetyl-CoA acetyltransferase | -7.386 |
| **METHANE METABOLISM** | | |
| **P-value 0.00033** | **Count 6/38** | **15.8%** |
| *BCAL2955* | *serC* - Phosphoserine aminotransferase | -2.204 |
| *BCAM0293* | *ackA* - Putative acetate kinase | -59.243 |
| *BCAM0298* | Putative phosphate acetyl/butyryl transferase | -76.193 |
| *BCAM0311* | Putative 6-phosphofructokinase | -65.666 |
| *BCAM2195* | Putative AMP-binding protein | -3.995 |
| *BCAS0739* | Putative acetyl-CoA synthetase | -5.646 |
| **BUTANOATE METABOLISM** | | |
| **P-value 0.00050** | **Count 7/58** | **12.1%** |
| *BCAL0409* | *paaF* - Enoyl-CoA hydratase | -2.357 |
| *BCAM0296* | Acetoacetyl-CoA reductase | -76.193 |
| *BCAM0297* | Putative poly(3-hydroxyalkanoate) polymerase | -76.193 |
| *BCAM0969* | *sdhA* - Succinate dehydrogenase flavoprotein subunit | -2.639 |
| *BCAM0970* | *sdhB* - Succinate dehydrogenase iron-sulfur subunit | -2.610 |
| *BCAM1250* | Putative acetyl-CoA hydrolase/transferase | -1.882 |
| *BCAS0737* | Putative acetyl-CoA acetyltransferase | -7.386 |
| **RNA DEGRADATION** | | |
| **P-value 0.00085** | **Count 4/17** | **23.5%** |
| *BCAL2348* | *pnp* - Polynucleotide phosphorylase/polyadenylase | -1.936 |
| *BCAL3146* | Chaperonin GroEL | -25.424 |
| *BCAL3270* | *dnaK* - Molecular chaperone DnaK | -9.979 |
| *BCAS0637* | Chaperonin GroEL | -34.865 |
| **D-ALANINE METABOLISM** | | |
| **P-value 0.00566** | **Count 2/4** | **50.0%** |
| *BCAL0705* | Putative D-amino acid aminotransferase | -2.748 |
| *BCAM1204* | Alanine racemase | -15.392 |
| **GLYOXYLATE AND DICARBOXYLATE METABOLISM** | | |
| **P-value 0.00658** | **Count 6/68** | **8.8%** |
| *BCAM0296* | Acetoacetyl-CoA reductase | -76.193 |
| *BCAM0961* | Aconitate hydratase | -2.465 |
| *BCAM0972* | *gltA* - Type II citrate synthase | -2.215 |
| *BCAM2817* | Glycolate oxidase subunit GlcD | -9.100 |
| *BCAM2818* | Glycolate oxidase FAD binding subunit | -9.100 |
| *BCAS0737* | Putative acetyl-CoA acetyltransferase | -7.386 |
| **ONE CARBON POOL BY FOLATE** | | |
| **P-value 0.00860** | **Count 3/16** | **18.8%** |
| *BCAL0075* | *gcvT* - Glycine cleavage system aminomethyltransferase T | -18.026 |
| *BCAL0147* | *metF* - 5,10-methylenetetrahydrofolate reductase | -4.955 |
| *BCAL3336* | purH - Bifunctional phosphoribosylaminoimidazolecarboxamide formyltransferase/IMP cyclohydrolase | -3.030 |
| **BACTERIAL SECRETION SYSTEM** | | |
| **P-value 0.00860** | **Count 5/51** | **9.8%** |
| *BCAL0254* | *secY* - Preprotein translocase subunit SecY | -4.083 |
| *BCAL0742* | *secB* - Preprotein translocase subunit SecB | -1.928 |
| *BCAL3307* | *secF* - Preprotein translocase subunit SecF | -5.013 |
| *BCAL3433* | *ffh* - Signal recognition particle protein | -2.793 |
| *BCAL3453* | *secA* - Preprotein translocase subunit SecA | -2.055 |
| **FATTY ACID DEGRADATION** | | |
| **P-value 0.01172** | **Count 4/35** | **11.4%** |
| *BCAL0409* | *paaF* - Enoyl-CoA hydratase | -2.357 |
| *BCAM0286* | Putative alcohol dehydrogenase | -17.616 |
| *BCAM1570* | Alcohol dehydrogenase | -139.172 |
| *BCAS0737* | Putative acetyl-CoA acetyltransferase | -7.386 |
| **NAPHTHALENE DEGRADATION** | | |
| **P-value 0.01172** | **Count 2/6** | **33.3%** |
| *BCAM0286* | Putative alcohol dehydrogenase | -17.616 |
| *BCAM1570* | Alcohol dehydrogenase | -139.172 |
| **TERPENOID BACKBONE BIOSYNTHESIS** | | |
| **P-value 0.01475** | **Count 3/20** | **15.0%** |
| *BCAL1884* | *ispG* - 4-hydroxy-3-methylbut-2-en-1-yl diphosphate synthase | -2.679 |
| *BCAM0911* | *dxs* - 1-deoxy-D-xylulose-5-phosphate synthase | -1.770 |
| *BCAS0737* | Putative acetyl-CoA acetyltransferase | -7.386 |
| **NUCLEOTIDE EXCISION REPAIR** | | |
| **P-value 0.01529** | **Count 2/7** | **28.6%** |
| *BCAL0825* | *uvrA* - excinuclease ABC subunit A | -3.135 |
| *BCAL2017* | *mfd* - transcription-repair coupling factor | -2.758 |
| **VITAMIN B6 METABOLISM** | | |
| **P-value 0.01955** | **Count 2/8** | **25.0%** |
| *BCAL2782* | *pdxH* - Pyridoxamine 5'-phosphate oxidase | -4.034 |
| *BCAL2955* | *serC* - Phosphoserine aminotransferase | -2.204 |
| **PORPHYRIN AND CHLOROPHYLL METABOLISM** | | |
| **P-value 0.02107** | **Count 4/43** | **9.3%** |
| *BCAL0264* | *hemB* - Delta-aminolevulinic acid dehydratase | -2.457 |
| *BCAL1711* | *cobN* - Cobaltochelatase subunit | -9.733 |
| *BCAL1712* | Putative magnesium chelatase protein | -9.733 |
| *BCAL3094* | *hemN* - Coproporphyrinogen III oxidase | -36.590 |
| **NITROGEN METABOLISM** | | |
| **P-value 0.02208** | **Count 3/24** | **12.5%** |
| *BCAL1830* | Putative 2-nitropropane dioxygenase | -53.392 |
| *BCAL3359* | Putative glutamate dehydrogenase | -9.125 |
| *BCAM2815* | Putative 2-nitropropane dioxygenase | -11.758 |
| **AMINOBENZOATE DEGRADATION** | | |
| **P-value 0.02409** | **Count 3/25** | **12.0%** |
| *BCAL0409* | *paaF* - Enoyl-CoA hydratase | -2.357 |
| *BCAM0810* | Putative aromatic oxygenase | -10.983 |
| *BCAM0811* | Putative aromatic oxygenase | -6.831 |
| **GERANIOL DEGRADATION** | | |
| **P-value 0.02758** | **Count 2/10** | **20.0%** |
| *BCAL0409* | *paaF* - Enoyl-CoA hydratase | -2.357 |
| *BCAM2196* | Putative acyl-CoA dehydrogenase | -5.913 |
| **PHENYLALANINE, TYROSINE AND TRYPTOPHAN BIOSYNTHESIS** | | |
| **P-value 0.02829** | **Count 3/27** | **11.1%** |
| *BCAL1467* | *aroC* - chorismate synthase | -3.171 |
| *BCAL2303* | Aromatic amino acid aminotransferase | -2.049 |
| *BCAL2952* | *aroA* - 3-phosphoshikimate 1-carboxyvinyltransferase | -3.902 |
| **PENTOSE PHOSPHATE PATHWAY** | | |
| **P-value 0.03047** | **Count 3/28** | **10.7%** |
| *BCAL2433* | *tal* - Transaldolase B | -1.687 |
| *BCAL3389* | *tktA* - Transketolase | -3.302 |
| *BCAM0311* | Putative 6-phosphofructokinase | -65.666 |
| **PHOSPHONATE AND PHOSPHINATE METABOLISM** | | |
| **P-value 0.03097** | **Count 2/11** | **18.2%** |
| *BCAM1243* | Putative aminotransferase | -2.093 |
| *BCAM1245* | Putative phosphoenolpyruvate phosphomutase/sugar nucleotidyltransferase | -2.093 |
| **FATTY ACID METABOLISM** | | |
| **P-value 0.03160** | **Count 4/51** | **7.8%** |
| *BCAL0409* | *paaF* - Enoyl-CoA hydratase | -2.357 |
| *BCAL2080* | *fabZ* - (3R)-hydroxymyristoyl-ACP dehydratase | -3.301 |
| *BCAL2719* | Putative transmembrane fatty acid desaturase | -2.523 |
| *BCAS0737* | Putative acetyl-CoA acetyltransferase | -7.386 |
| **CARBON FIXATION IN PHOTOSYNTHETIC ORGANISMS** | | |
| **P-value 0.04087** | **Count 2/13** | **15.4%** |
| *BCAL3388* | *gapA* - Glyceraldehyde 3-phosphate dehydrogenase 1 | -4.547 |
| *BCAL3389* | *tktA* - Transketolase | -3.302 |
| **BETA-LACTAM RESISTANCE** | | |
| **P-value 0.04547** | **Count 3/34** | **8.8%** |
| *BCAL1674* | *amrA* - Periplasmic multidrug efflux lipoprotein | -7.862 |
| *BCAL1675* | *amrB* - Multidrug efflux protein | -6.194 |
| *BCAL2820* | *oprM* - Efflux system outer membrane protein | -4.056 |
| **BETA-LACTAM RESISTANCE** | | |
| **P-value 0.04547** | **Count 3/34** | **8.8%** |
| *BCAL2303* | Aromatic amino acid aminotransferase | -2.049 |
| *BCAM0286* | Putative alcohol dehydrogenase | -17.616 |
| *BCAM1570* | Alcohol dehydrogenase | -139.172 |
| **TAURINE AND HYPOTAURINE METABOLISM** | | |
| **P-value 0.05023** | **Count 2/15** | **13.3%** |
| *BCAM0293* | *ackA* - Putative acetate kinase | -59.243 |
| *BCAM0298* | Putative phosphate acetyl/butyryl transferase | -76.193 |
| **DNA REPLICATION** | | |
| **P-value 0.05549** | **Count 2/16** | **12.5%** |
| *BCAL0422* | *dnaN* - DNA polymerase III subunit beta | -1.970 |
| *BCAL1899* | *dnaX* - DNA polymerase III subunits gamma and tau | -2.607 |
| **CHLOROALKANE AND CHLOROALKENE DEGRADATION** | | |
| **P-value 0.05964** | **Count 2/17** | **11.8%** |
| *BCAM0286* | Putative alcohol dehydrogenase | -17.616 |
| *BCAM1570* | Alcohol dehydrogenase | -139.172 |
| **2-OXOCARBOXYLIC ACID METABOLISM** | | |
| **P-value 0.05964** | **Count 3/39** | **7.7%** |
| *BCAL1059* | *argM* - Bifunctional N-succinyldiaminopimelate-aminotransferase/acetylornithine transaminase protein | -9.857 |
| *BCAM0961* | Aconitate hydratase | -2.465 |
| *BCAM0972* | *gltA* - Type II citrate synthase | -2.215 |
| **FLAGELLAR ASSEMBLY** | | |
| **P-value 0.06202** | **Count 3/40** | **7.5%** |
| *BCAL0114* | *fliC* - Flagellin | -1.921 |
| *BCAL0126* | *motA* - Flagellar motor protein MotA | -1.913 |
| *BCAL0576* | *flgK* - Flagellar hook-associated protein FlgK | -2.055 |
| **MISMATCH REPAIR** | | |
| **P-value 0.06373** | **Count 2/18** | **11.1%** |
| *BCAL0422* | *dnaN* - DNA polymerase III subunit beta | -1.970 |
| *BCAL1899* | *dnaX* - DNA polymerase III subunits gamma and tau | -2.607 |
| **LYSINE BIOSYNTHESIS** | | |
| **P-value 0.08943** | **Count 2/22** | **9.1%** |
| *BCAL1059* | *argM* - Bifunctional N-succinyldiaminopimelate-aminotransferase/acetylornithine transaminase protein | -9.857 |
| *BCAM2076* | *lysA* - Diaminopimelate decarboxylase | -2.864 |
| **BETA-ALANINE METABOLISM** | | |
| **P-value 0.10760** | **Count 2/25** | **8.0%** |
| *BCAL0409* | *paaF* - Enoyl-CoA hydratase | -2.357 |
| *BCAM2194* | *mmsA* - Methylmalonate-semialdehyde dehydrogenase | -4.876 |
| **HOMOLOGOUS RECOMBINATION** | | |
| **P-value 0.10760** | **Count 2/25** | **8.0%** |
| *BCAL0422* | *dnaN* - DNA polymerase III subunit beta | -1.970 |
| *BCAL1899* | *dnaX* - DNA polymerase III subunits gamma and tau | -2.607 |
| **CYSTEINE AND METHIONINE METABOLISM** | | |
| **P-value 0.13558** | **Count 2/29** | **6.9%** |
| *BCAL0145* | *ahcY* - S-adenosyl-L-homocysteine hydrolase | -3.820 |
| *BCAL2303* | Aromatic amino acid aminotransferase | -2.049 |
| **AMINO SUGAR AND NUCLEOTIDE SUGAR METABOLISM** | | |
| **P-value 0.20468** | **Count 2/38** | **5.3%** |
| *BCAL0611* | *glmS1* - glucosamine--fructose-6-phosphate aminotransferase | -3.240 |
| *BCAL1722* | Putative exported chitinase | -9.498 |
| **BENZOATE DEGRADATION** | | |
| **P-value 0.26684** | **Count 2/46** | **4.4%** |
| *BCAL0409* | *paaF* - Enoyl-CoA hydratase | -2.357 |
| *BCAS0737* | Putative acetyl-CoA acetyltransferase | -7.386 |
| **DEGRADATION OF AROMATIC COMPOUNDS** | | |
| **P-value 0.33540** | **Count 2/55** | **3.6%** |
| *BCAM0286* | Putative alcohol dehydrogenase | -17.616 |
| *BCAM1570* | Alcohol dehydrogenase | -139.172 |
| **SULFUR METABOLISM** | | |
| **P-value 0.33788** | **Count 2/56** | **3.6%** |
| *BCAL1819* | NAD(FAD)-dependent dehydrogenase | -13.704 |
| *BCAL3216* | *cysC* - Adenylyl-sulfate kinase | -2.024 |
| **ABC TRANSPORTERS** | | |
| **P-value 0.65953** | **Count 5/264** | **1.9%** |
| *BCAL0043* | Putative extracellular ligand-binding protein | -4.156 |
| *BCAL1055* | Histidine transport system permease | -3.966 |
| *BCAL1056* | Histidine transport system permease | -3.966 |
| *BCAL1057* | Histidine ABC transporter ATP-binding protein | -3.759 |
| *BCAL1065* | Periplasmic solute-binding protein | -6.270 |
